# Kansas City Brain Builders: associations between a multisectoral community language nutrition intervention and change in practices and growth in child language over two years

**DOI:** 10.3389/fpubh.2026.1986929

**Published:** 2026-10-28

**Authors:** Charles R. Greenwood, Gabriela Guerrero, Alana G. Schnitz, Sandra Cintora, Christine Giancola, Judith J. Carta

**Affiliations:** Juniper Gardens Children’s Project, University of Kansas, Lawrence, KS, United States

**Keywords:** home language environment, language acquisition, language nutrition, multisector community partnership, parent/caregiver-child interaction, public health, word gap

## Abstract

**Background and objectives:**

Health literacy in adulthood plays a major role in an individual’s ability to use health information to benefit their health. Social determinants in early childhood initiate the development of health literacy through newborn brain development and oral language acquisition. Both of these processes are promoted by stimulation from everyday interactions with parents/caregivers at home and in the community. Unequal access to language-learning opportunities during early childhood represents a modifiable developmental and social determinant of later educational and health outcomes. There are inequities in these time-sensitive interactions—specifically language-learning opportunities—across families in the population, which are social determinants of early literacy, achievement in school, and later-life education, health, and economic and social well-being across the life span. This gap is most pronounced among children who grow up in poverty in the United States and internationally. The healthcare system in the United States effectively reaches to nearly all infants; however, language-learning opportunities are not a focus during well-child visits. The term “Language Nutrition” (LN) was coined in prior research to refer to these developmentally rich interactions known to support brain and language growth in children younger than preschool age. We developed Kansas City Brain Builders (KCBB) to address this inequity by promoting LN broadly among families and children in a community so that all children have a more equitable home language environment. The purpose of this study is to report longitudinal empirical findings on families and children related to the implementation of KCBB, a public health approach to community change, over 2 years.

**Methods:**

The evaluation involved two parallel longitudinal studies: the Community study and the Family study. The Community study assessed the implementation of a KCBB multisectoral community partnership to promote LN-friendly changes in local programs, practices, and policies. The Family study involved longitudinal assessments of a sample of 83 English- and Spanish-speaking families with children younger than 36 months of age, evaluating the KCBB impacts on parental behaviors and child growth in language skills.

**Results:**

KCBB led to a high level of documented program implementation and substantial LN promotion throughout the community. Repeated parent assessments indicated that participants had received LN messages from multiple community sectors over time, increased their knowledge of the importance of LN, and used LN through storybook reading, verbal responsiveness, and teaching behaviors during interactions with their children. Growth in children’s early language skills was significantly associated with the amount of LN they received.

**Discussion/Conclusion:**

KCBB is one of the few multisectoral interventions designed to promote community-wide change targeting LN. It is a manualized intervention that can be replicated by other communities. This study employed three levels of measurement such that change could be evaluated in a theoretical framework of influence from community engagement to parental behavior and growth in child language skills. Findings provide preliminary evidence that a multisectoral community approach can achieve broad reach and is associated with changes in caregiver practices and child language growth. Larger, controlled, and replicated studies are needed to establish causal effects and determine whether such approaches reduce disparities in language-learning opportunities.

## 1 Background

Health literacy in adulthood plays a major role in an individual’s ability to use health information to the benefit of their health. Social determinants in early childhood initiate the development of health literacy through newborn brain development and oral language acquisition. Both are promoted by the stimulation provided by everyday interactions with parents and caregivers at home and in the community. Unequal access to language-learning opportunities during early childhood represents a modifiable developmental and social determinant of later educational and health outcomes. There are inequities in these time-sensitive interactions—specifically language-learning opportunities—across families in the population that shape early literacy, achievement in school, and later educational, health, and economics and social well-being across the lifespan. This gap is greatest among children reared in poverty.

Early childhood is a period of rapid brain and language development during which everyday interactions with caregivers and other adults provide important opportunities for learning (1–7). The term, “language nutrition” (LN), was coined to describe these developmentally rich interactions, the social determinants known to support brain and language growth (2). Frequent and reciprocal interactions and verbal exchanges between young children and their parents/caregivers, are essential to early cognitive development just as nutritious food is to physical development (2, 7, 8).

Research reports indicate wide variation in the language-learning opportunities that individual infants and toddlers receive across the population (9, 10), leading to differential oral language, literacy, reading, and academic success (11–13). The probability of experiencing fewer language-learning opportunities, underachievement, and school failure is highest for individuals from low-income environments in the United States (14) and a pattern in other countries worldwide (15). Children with delays in oral language are at risk of failing to achieve proficient reading skills by grade 4, a developmental juncture when pedagogical focus shifts from learning to read to reading to learn subject matter thereafter. Although early work often described these disparities as a “word gap,” the present study adopts an opportunity-oriented framing, emphasizing unequal access to language-learning experiences and the modifiable community conditions that shape those opportunities.

While a number of evidence-based interventions and approaches exist for supporting individual children, we identified no programmatic frameworks designed to address this population-level problem (16). Given that inequities in LN are both well-documented and persistent (9), achieving meaningful progress requires moving beyond individual interventions to broader, systemic approaches. Public health offers one such evidence-based approach for preventing disease, promoting safety, educating the public, and prolonging life in entire communities and the population (see Carta et al., paper 1, this issue of *Frontiers in Public Health*).

KCBB is one of the few evidence-based multisectoral language interventions targeting the community and the population of parents and children living there. It is not an individualized child-focused intervention. KCBB responds to this challenge by engaging public systems, media platforms, multiple community settings, and aligned interventions to foster LN-rich language environments that promote LN for all children at home, beginning at birth, across sectors, neighborhoods, and demographics (16–18). Community-level interventions promoting LN in KCBB are implemented by stakeholders and organizations within a coalition of community sectors, i.e., pediatric healthcare, home visiting programs, childcare programs, libraries, laundromats, mayor’s office, etc. Thus, it fits public health, prevention science, and community psychology frameworks (19). By synthesizing evidence-based LN practices with the multilevel, multisectoral community partnership frameworks from community psychology, we set out to develop a public health intervention to promote LN (19).

Kansas City Brain Builders (KCBB) was developed to serve as this intervention (19, 20). Our goal was to translate knowledge related to LN promotion into a practical means of changing parenting behavior community-wide (21). We sought to create equal opportunities for all children to learn language across families, without necessarily expecting identical developmental outcomes (22).

### 1.1 Kansas City Brain Builders (KCBB)

We developed KCBB to address several key limitations in existing literature. First, the lack of language intervention models for achieving population-level impacts (19, 23). Recently, however, McKean & Reilly (24) proposed a public health systems approach to language promotion. Second, there is limited understanding of how to build and sustain effective community coalitions that support early language development in low-socioeconomic status (SES) communities, as well as a general lack of implementation capacity in such contexts (16). Third, prior studies have employed insufficient measurement models for capturing change and improvement in multicomponent community interventions (25–27).

We used an iterative process grounded in a public health prevention approach and ecobehavioral theory to develop KCBB (28, 29). We integrated evidence from language intervention research with a multisectoral community partnership into a strategic planning model, the Bridging the Word Gap Action Planning Guide [the Guide] (30, 31). The Guide empowered local leaders to assess community needs, organize initiatives, set goals, engage sector leaders, select feasible language interventions, and track implementation progress across sectors.

KCBB aims to elevate public understanding of children’s LN and actionable strategies to support home language environments through coordinated messaging, professional development, public events, and embedded practices across pediatric care, libraries, social media, public settings, and the home. KCBB disseminates sector-aligned evidence-based LN strategies to support caregiver–child interactions (19, 20).

Community leaders guided by the framework reviewed and tailored-specific sector LN interventions to the unique strengths and capacities of each participating community sector (32). This flexible, sector-specific selection of evidence-based language interventions supports the feasibility of implementation and thus creates language-learning equity within and across communities (19, 22, 32, 33).

We conducted a pilot test of KCBB across three sectors—pediatric health care, childcare, and research/evaluation. Two LN interventions were implemented: *Talk with Me Baby* (TWMB) (7, 17, 34, 35) in a hospital setting, training nurses to coach parents of newborns; and *LENA Grow* (36) in neighborhood infant–toddler centers, using digital technology and coaching to boost verbal interactions. The research/evaluation sector supported training, documentation, and measurement through the Community Check Box Evaluation System (25), tracking 283 actions across multiple community change categories. Primary outcomes evaluated included implementation fidelity, sector engagement, and child language outcomes (20, 37).

We conducted a full-scale longitudinal trial of KCBB in Kansas City, with two coordinated evaluations: the Community study (32) and the Family study. The Family study is the focus of this report. The Community study involved 12 sectors and 21 organizations that implemented 329 targeted changes in practices, programs, and policies across sectors, and is reported separately (32). The Family study was designed to examine whether implementation of the community-wide initiative was accompanied by changes in caregiver experiences and language practices and whether caregiver LN was associated with children’s longitudinal language development. The most active sectors were research/evaluation, childcare, home/family, healthcare, and the library. Examples included coalition leaders’ videoconference meetings for strategic planning, training medical interns and residents to implement the TWMB intervention, childcare provider training on talking tips and interaction strategies, and establishing a library infant/toddler center with language materials. Additional outreach distributed posters and tips through a local restaurant and laundromats.

Through structured outreach, messaging, training, and resources, KCBB fostered a community-wide environment supporting high-quality language interactions across settings. Evaluation indicated a substantial level of KCBB implementation and LN promotion through changes made by local leaders, multiple organizations, and sectors targeting families with children younger than 36 months of age (32).

### 1.2 Purpose

The purpose of this study is to report longitudinal empirical findings for families and children associated with the implementation of KCBB over 2 years. While the first report examined community-level outcomes—highlighting changes in practices, programs, and policies across sectors linked to KCBB implementation (32), this report examines longitudinal outcomes at the family and child levels.

Here we examined a multilevel hypothesis concerning the associations among strategic changes in (a) community environment addressing language-supportive structural policies, programs, and practices; (b) reach to parents through their reported experiences; and (c) changes in children’s home language environments, specifically language experiences with language nutrition (LN) provided by their caregivers (see Figure 1). The hypothesis was examined in a two-year longitudinal study of the experiences of parents/caregivers, changes in home language environments and their associations with repeatedly measured language skills in a sample of 83 community residents and their children.

**Figure 1:**
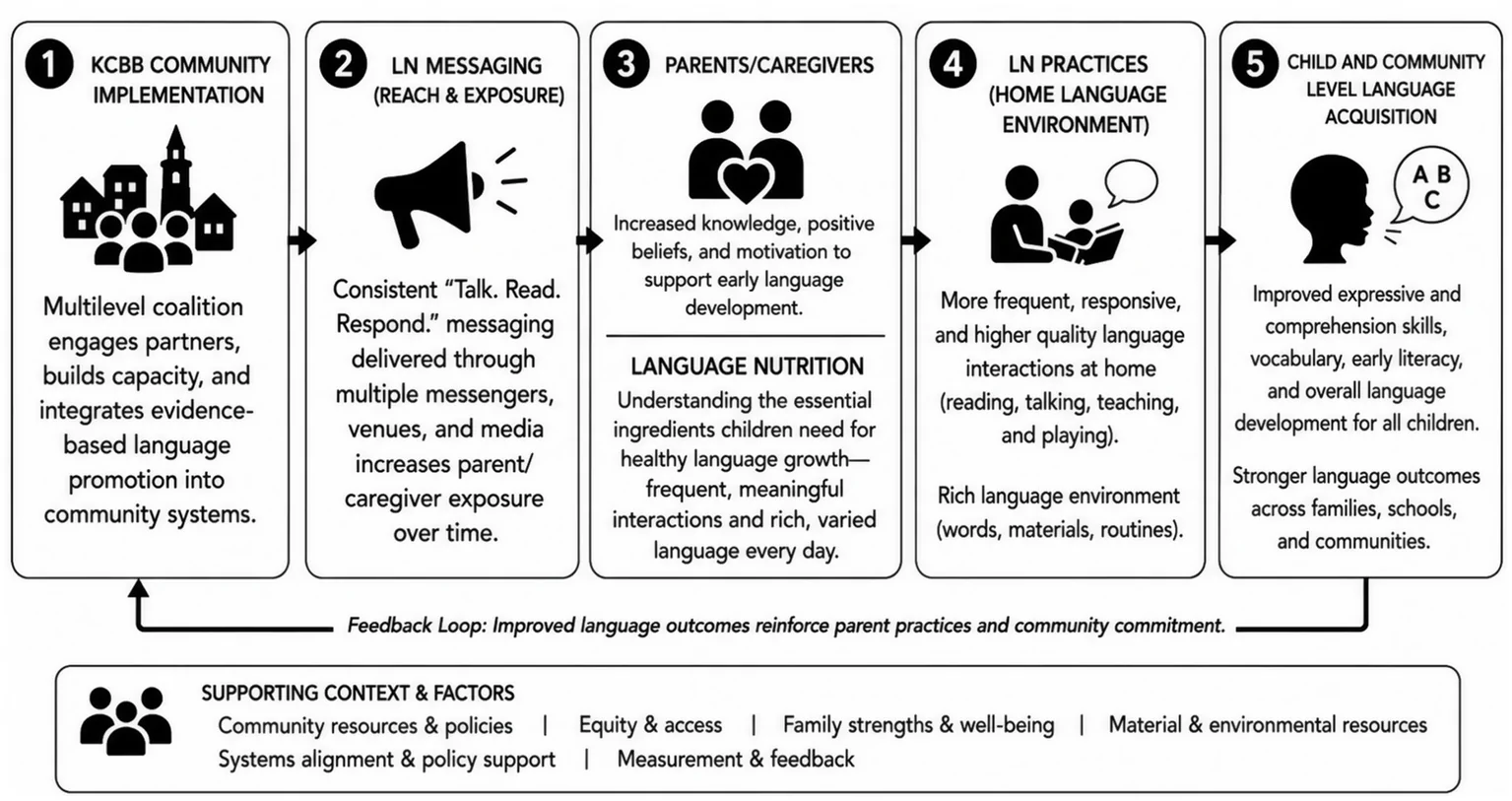
KCBB multilevel pathways to all children’s language learning.

### 1.3 Aims and specific research questions

*Aim 1*: To evaluate caregiver exposure to KCBB and association with caregiver-reported LN practices.

- *Research Question 1*: To what extent did parents and caregivers report exposure to KCBB-aligned messages across community sectors? We hypothesized that KCBB multisector strategies would be evident in families’ reported experiences.
- *Research Question 2*: We reported KCBB-related experiences associated with caregiver-reported LN practices at home as measured by the StimQ2 Cognitive Home Environment Checklist. We expected greater exposure to KCBB to be positively associated with these supportive language promotion practices.

*Aim 2*: To examine longitudinal children’s early language growth and associations with caregiver-reported LN.

- *Research Question 3*: Which child and family characteristics—including LN, were associated with children’s early language growth, as measured by the LENA Developmental Snapshot? LN was hypothesized to be a key modifiable predictor of language growth, given shifts in the surrounding community context.

## 2 Methods

### 2.1 Participants

#### 2.1.1 Communities

We recruited low-income families with young children from two neighboring inner-city communities in the Kansas City metropolitan area, located on opposite sides of the Missouri River—one in Kansas and the other in Missouri. Each community was defined by the catchment area of a low-SES high school, wherein the majority of students qualified for Title I services. To assess the resources and conditions available to families to help children thrive, we used the Child Opportunity Index (COI) (38), which rated the ZIP code COI scores for both communities as “very low” across education, health/environment, and social/economic domains.

#### 2.1.2 Parents and children

A total of 90 families were recruited for a 2-year longitudinal study of child language development, without prior knowledge of the broader community intervention. Parents received quarterly compensation and feedback on their child’s early language progress over 2 years. The eligibility criteria included the following: (a) residence in one of the two target high school catchment areas, (b) primary caregiving for a child under age 3 years, and (c) access to a digital device for completing online surveys and assessments. Recruitment sources included a hospital’s newborn registry, local childcare programs, social service agencies, and community outreach.

Eighty-three families were enrolled, with children averaging 8.5 months of age (*SD* = 6.0; range = 2–31 months). Table 1 presents child, parent, and household characteristics, including child age, gender, race, ethnicity, and health status, parent age, education, employment, income, poverty status, language preference, and household size.

**Table 1:** Participant characteristics.

Participant	Variable	Value	Sample (*N* = 83)			
			*n*	*M*	SD/(Min, Max)	%
Child	Age	Months	83	8.5	6.0/(2,31)	
	Gender	Female	45			56
		Male	35			44
	Race	Black	4			5
		Asian	2			3
		NativeAm	2			3
		White	29			36
		MultiRace	9			11
		Other	34			43
	Ethnicity	Hispanic	56			70
	Health	Good	5			6
		Very Good	17			21
		Excellent	58			73
Parent	Age	Years	62	30.0	6.7/(18,45)	
	Income	Annual $	80	41,030.85	$53,868 ($0.00,$250,000)	
		Poverty	54			65
	Parent Ed	LT High School	27			34
		High School/GED	33			41
		GT High School	20			25
	Employment	Working	34			43
		Not Work	46			58
	Survey Language Choice	English	33			41
		Spanish	47			59
Home/Neighborhood	Living at Home	#Persons	80	4.6	1.3/(2,8)	
	Side State Line	Missouri	13			17
		Kansas	66			84
	Child Opportunity Index	Combined Rating	15 Zip Codes	Very Low	(Very Low / High)	

### 2.2 Design and procedures

A longitudinal implementation evaluation was conducted using a multilevel design in which children were nested within families, and families were nested within communities (39, 40). Because launching the community-level initiative—KCBB (referred to as the Community Study)—required significant preparation, community implementation of KCBB began before the 83 families were enrolled in the related Family Study. The first documented community-level activity for KCBB took place on August 15, 2022, and continued throughout the study period. Most families were enrolled by March 15, 2023, for a 2-year commitment (coinciding with full implementation of the Community study), and 79 families remained in the study through March 15, 2025. Families began completing surveys at the time of enrollment (Occasion 1), which meant there was no true baseline measurement. The study design included several methodological strengths, including repeated assessments, English and Spanish surveys, and fully online data collection using digital tools.

### 2.3 Measures and reliability

#### 2.3.1 Community-level child opportunity

To assess the broader community context, we used the *child opportunity index* (COI) (38), a validated composite measure of neighborhood-level opportunity across three domains: (a) education, (b) health and environment, and (c) social and economic factors. The COI has been widely used to examine disparities in children’s access to developmental supports and to inform equity-focused policies and program intervention design (41). Using publicly available data, we obtained COI scores for each of the 15 zip codes where participating families resided. Possible scores ranged from 1 (very low) to 5 (very high).

#### 2.3.2 Family- and child-level measurement

Parents/caregivers completed a repeated, multimeasure protocol of surveys administered entirely online. These included the *Child/Family Survey*, *Parent Community Experiences Survey*, the *StimQ2 Cognitive Home Environment Checklist*, and the *LENA Developmental Snapshot* (42) (these measures are available from the first author). All four were administered online, the first three using the REDCap data capture software (43), and the last through the LENA Online application.

##### 2.3.2.1 Child–family sociodemographics (child/family survey)

The *Child/Family Survey* was developed by the research team to assess 32 items nested under four domains: the child, parent/caregivers, the household, and the neighborhood. We drew some of the items from the National Survey of Children’s Health (44). We administered the survey following consent and enrollment (Occasion 1). We computed the federal poverty level using annual family income and the number of children in the family per 2023 benchmarks for use in analyses.

##### 2.3.2.2 KCBB reach, intensity, and impact (parent community experiences survey)

The Parent Community Experiences Survey was a 14-item measure administered to parents/caregivers on 12 occasions spaced 2 months apart over 24 months. It was developed specifically for KCBB and designed to assess parents’/caregivers’ exposure to the KCBB messaging campaign where the construct was defined by the Guide (20). Thus, the measure had content validity referenced to the Guide.

Items were used to address three constructs: (a) reach to caregivers/parent; (b) intensity or dosage; and (c) impact on what caregivers/parents learned. Items included Yes/No, Mark All That Apply, Multiple-Response, and True/False formats with conditional branching for additional details. How the item scores for each construct were used over time graphical display; (see Figure 1) or collapsed over time in analyses is shown in Table 2.

**Table 2:** Community experiences survey scores details.

Construct	Variable	Definition	Calculation/Metric	Question(s)	Metric
Reach of KCBB’s Messaging Campaign	Did Anyone Tell You?	Learned the importance of talking to babies	Sum of Yes, I have been told	Has anyone told you about the importance of talking to babies?	Yes/No
	Who Told You?	Community sectors that provided the message	Check all that apply out of 11 sectors (e.g., health provider, library, etc.)	Which community sector(s) provided the message?	All that Apply
	How Received?	How the message was delivered	Check all that apply out of 5 mediums (e.g., social media, a conversation, etc.)	How was the message conveyed to you?	All that Apply
	Where Were You?	My location when message received	Check all that apply out of 12 community locations (e.g., at work, home, etc.)	Where in the community were you when received?	All that Apply
Dosage	Depth of Messaging	Strength and success of the campaign on the caregiver	(Sum (talktobaby + language_nutrition + wordgap + received_instruction))	Who shared this information with you? Has anyone told you about language nutrition? Has anyone told you about the word gap? Have you received instruction, coaching, or talking tips as a way to build your child’s brain and language?	All that Apply and Number Correct
Impact of Campaign on Caregiver	Talk Knowledge	What the caregiver learned about when and good times to talk	Sum (best_age to start talking + best_age to start reading + diapering + babbling + imitating)	What is the best age to start talking to your baby? What is the right age to start looking at and sharing books with your child? Is diapering with a child a good time to have a conversation? Is it better to respond to my baby’s babbles with baby talk instead of my own words? Is imitating what my child says a good way to promote his/her use of words?	Number Correct
	What I Learned	What the caregiver learned about why and how to talk	Sum (builds_brain + when_learning_starts + helps_learntotalk + starts_birth + startstalk_frequently_take_ turns + I_have_thepower)	Talking helps build my child’s brain. Real learning starts in kindergarten. Talking helps children learn to talk and use their language. Learning starts at birth Best to talk frequently and take turns each day? Best to talk only when the child talks to me. I have the power to make a difference in my child’s development	True or False
	Interaction Tips	How the caregiver learned their talking tips	Sum (brochure + coach + workshop + at_a_meeting + friend + website + literacy_center + talk_read_play + church_meeting)	From a brochure or pamphlet From a home visitor or coach At a workshop or class you attended At a meeting = An in-person or online Zoom call community meeting From a friend or family member Website, Smartphone, from an App, social media, or website While at a family literacy center or meeting From a talk-play-read- sing learning materials kit At a church meeting or from a church member	Check all that Apply

##### 2.3.2.3 Language nutrition StimQ2 cognitive home environment checklist

Caregiver-provided LN was assessed using the *StimQ2* administered on four occasions over 24 months (45). Core constructs assessed parent’s behaviors linked to *Book Reading* (READ—*Do you read books to your child that teach about simple shapes such as squares, circles, and triangles?*), *Parent Involvement in Developmental Advance—*(TEACH*—Do you teach you child about body parts by playing with them and touching parts of their body while saying the name?* (e.g.*,* “Here is baby’s nose”), and *Parent Verbal Responsivity* (PVR*—Do you play peek-aboo games with your infant such as hiding your face and revealing yourself?*). Each item captured a specific parent behavior they might engage in during home interactions (i.e., book reading, teaching, and verbal responsiveness). Adequate validity and reliability metrics are reported (45).

Both the Infant and Toddler StimQ2 versions were used, with most children completing the Infant version at Occasion 1 and progressively transitioning to the Toddler version in later waves (Occasion 1: 95.1% Infant; Occasion 4: 100% Toddler). While some items and scales varied between versions, quantity-based questions (e.g., number of books, reading days per week) and the TEACH scale remained consistent. The LN scores used in analyses were a composite of the percentage of endorsed items across each StimQ2 construct (READ, TEACH, and PVR).

Because the infant and toddler versions differed in item composition, we created an LN index developed specifically for this study to permit longitudinal comparisons. We calculated the percentage of endorsed items for each domain (100 × [items endorsed ÷ total items]) to account for item differences between versions. Additional quantitative scores (e.g., number of books read, days read to the child) and qualitative text comments were used in descriptive analyses.

##### 2.3.2.4 Children’s receptive (comprehension) and expressive (gestural and vocal) skills

###### 2.3.2.4.1 The *LENA Developmental Snapshot* (42)

The *Snapshot* assesses children’s receptive (comprehension) and expressive (vocal) language skills. It is a 52-item, parent-completed measure of infants’ and toddlers’ language skills. It was administered on four occasions over 24 months. Occasions were at start = 1, 8 months = 2, 16 months = 3, and 24 months = 4. As a group, children had a mean (SD) of 8.2, 14.1, 20.0, and 25.5 months in chronological age at Occasions 1, 2, 3, and 4, respectively.

Domains of focus included vocal behavior and preverbal communication for infants under 12 months, responsiveness to instruction, spontaneous speech production, and vocabulary development for 1-year-olds; and conceptual and grammatical development for children older than 24 months. Domains for older children included nonverbal behavior, vocabulary and grammatical development, and semantic concept formation.

Reliability (test–retest, *r* = 0.95) and criterion validity (*r* = 0.67–0.97) were previously examined in a sample of 60 parents who completed the *Snapshot* checklist monthly for their child. Children were also evaluated by speech-language pathologists (SLPs) who administered criterion standard assessments. Sensitivity and specificity for language delay detection were 87%. Developmental age scores were used to track growth over time while standard scores (*M* = 100, *SD* = 15) assessed normative performance. Both metrics were used to characterize longitudinal patterns in children’s early language acquisition.

### 2.4 Statistical methods

For Aim 1, we used graphical and tabular displays to describe KCBB community reach and the home language environment, examining Pearson correlation coefficients between reach indicators and StimQ2 Toddler scores at Occasion 4. We used Occasion 4 because all children received the Toddler level items. For Aim 2, we applied multilevel linear growth models (46) to account for repeated measures (ICC = 9.1), estimate fixed and random effects, and center time at Occasion 4. These models also accounted for missing data (46). This approach modeled growth in early language (*LENA Developmental Snapshot* scores) and enabled testing multiple models, including child characteristics (e.g., gender, poverty) and home LN on children’s language age-equivalent scores with model fit compared using information criteria.

## 3 Results

### 3.1 Trends in exposure to KCBB messaging

At the first assessment, only 20% of parents had not heard of the importance of talking to their child. This was not surprising because KCBB was already underway at the first assessment. By the final assessment, 90% had received the KCBB message at least once over the 2-year study period. Across 12 assessment points spaced 2 months apart, parents reported receiving KCBB messages from an average of four community sectors per occasion, reflecting broad multisectoral outreach aligned with coalition goals (32) (Figure 2).

**Figure 2:**
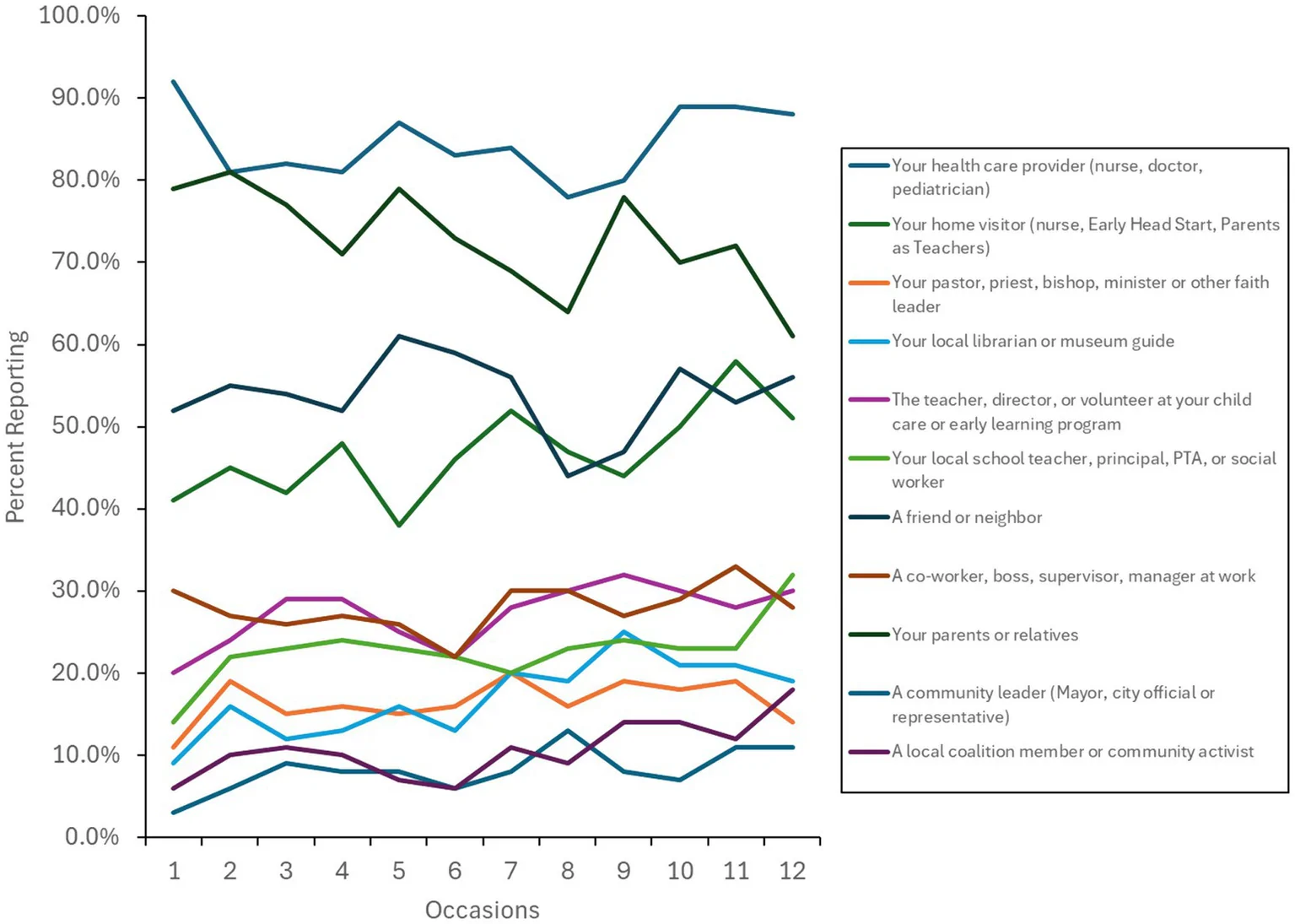
Parents’ reported messages received from KCBB community sectors regarding the importance of talking to their baby. The occasions correspond to the study calendar in months as follows: 1 = Start, 2 = 4 months, 3 = 6 months, 4 = 8 months, 5 = 10 months, 6 = 12 months, 7 = 14 months, 8 = 16 months, 9 = 18 months, 10 = 20 months, 11 = 22 months, and 12 = 24 months.

The sources of exposure reported by the greatest percentage of parents over 24 months were healthcare (*M* = 84%), followed by parents/relatives (*M* = 73%), friends/neighbors (*M* = 54%), and home visitors (*M* = 41%), with only parent/relative messaging declining over time. Messaging from other sectors (e.g., childcare, libraries, faith-based organizations) reached from *M* = 5 to 28% of families and remained stable or increased. Most parents reported receiving messages at home (*M* = 84%) or in clinical settings (*M* = 74%), conveyed to them primarily through conversations (*M* = 89%) and, to a lesser extent, social media or the Internet (*M* = 53%). The findings highlight the broad reach of the multisectoral approach to promoting early language through messaging and also suggest sectors with room for growth in the mix of KCBB’s activities.

### 3.2 Parent implementation of language nutrition practices over time

Parents reported using LN practices through Book Reading, Teaching, and Verbal Responsivity, with the greatest growth seen in Book Reading over 2 years (Figure 3).

Across the entire sample of parents/caregivers, 85–90% of parents reported reading books to their child by the final assessment point. On average, they read to their child about 4 days per week, often at bedtime or during daily routines. The availability of learning materials (books, toys, games) was higher at later assessment points than at enrollment, increasing from a mean of 57 to 76% on the StimQ2 Checklist. The average number of children’s books in the home rose from M = 17 (*SD* = 23) to M = 23 (*SD* = 26.5), ranging from 2 to 150 books per family. Because there was no true baseline, however, we interpret this increase cautiously, as it could have been due to maturation or repeated measurement.

### 3.3 Associations between KCBB community experiences and language nutrition at home

Significant positive associations were observed between parents’ participation in KCBB-aligned community experiences and the quality of their children’s home language environment as measured by the StimQ2 on Occasion 4 (see Table 3, Language Nutrition column). Of the eight community experience indicators, six showed significant positive correlations with Language Nutrition (*p* < 0.05): (a) *Who Shared*, (b) *How Received*, (c) *Did Anyone Share with You*, (d) *Talk Knowledge*, (e) *Depth of Messaging*, and (f) *Interaction Tips Guidance*.

**Table 3:** Correlations between KCBB community experiences and language nutrition reported by parents.

Variable	Language Nutrition	1	2	3	4	5	6	7
1. Who Told You	0.287*							
2. How Received	0.288*	0.785**						
3. Where Were You	0.223	0.876**	0.788**					
4. Did Anyone Tell You	0.284*	0.208	0.284*	0.114				
5. Talk Knowledge Composite	0.248	−0.01	0.033	−0.052	−0.057			
6. Depth of Messaging Composite	0.387**	0.364**	0.421**	0.269*	0.634**	0.045		
7. What I Learned Composite	0.263*	0.199	0.303**	0.11	0.958**	0.072	0.571**	
8. Interaction Tips Composite	0.323*	0.382**	0.425**	0.293**	0.397**	0.171	0.821**	0.393**

*Correlation is significant at the 0.05 level (2-tailed); **Correlation is significant at the 0.01 level (2-tailed). Language Nutrition is the composite percentage encoded Read, Teach, and Responsivity behaviors reported by parents on the StimQ2.

Qualitative written responses from the Community Experiences Survey item, *What did you learn*? supported these findings. English-speaking caregivers wrote: *Speaking and reading to your child at an early age is very good for their vocabulary development*. Spanish-speaking caregivers shared a similar insight: *El lenguaje nutritivo viene de los padres que le enseñan a los niños durante el crecimiento* (Nurturing language comes from parents teaching children as they grow up). These findings suggested that KCBB activities promoted caregivers’ greater understanding and language-nurturing practices in the home. This pattern is consistent with the hypothesis that exposure to KCBB-aligned community practices may be associated with caregiver knowledge and LN practices.

### 3.4 Predictors of growth in children’s early language development

Children’s language development showed significant gains in developmental age over the 2-year study period, increasing from an average of 7.6 months at baseline to 24.2 months by the final assessment (growth rate = 5.5 months per occasion) (Figure 4, upper panel). This was normative progress for a sample of low-SES families, English- and Spanish-speaking with children in a low-SES community.

**Figure 3:**
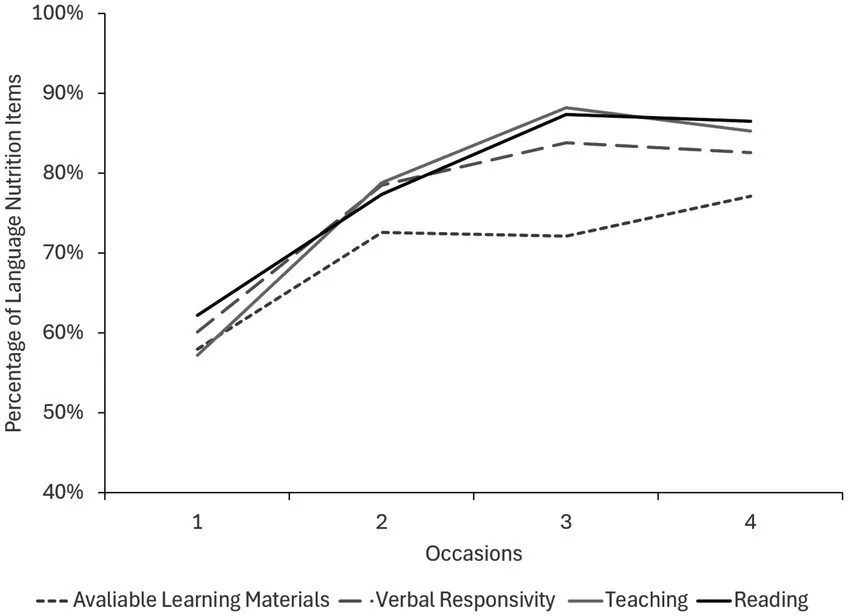
Plot of parents’ report of language nutrition over time measured by the StimQ2. The occasions correspond to the study calendar as follows: 1 = Start, 2 = 8 months, 3 = 16 months and = 24 months.

While language developmental ages improved, standard scores declined slightly (from *M* = 108.4 to 97.7; *slope* = −3.7 per occasion); see Figure 3-Lower Panel, a pattern not uncommon in early childhood research (47, 48). Because standard scores are relativistic, a child can improve in ability but still show a *lower standard score* if they improve more slowly than the norming group. Or, if a child’s development is steady, it may not be accelerating at the same pace as peers. By the end of the study, on average, children remained within normative development (*M* = 97.7) well above the −1 standard deviation.

LN was significantly associated with language growth in the final model and contributed substantially to model fit. For every 1-unit increase in LN, there was a corresponding 0.376 increase in children’s language months of age outcome. Growth parameter estimates are presented in Tables 4, 5.

**Table 4:** Growth model for early language outcome with poverty and language nutrition as predictors.

Estimates of fixed effects^a^	Estimate	SE	df	*t*	*p*	95% Confidence Interval	
						Lower bound	Upper bound
Intercept	17.082	3.590	62.771	4.759	<0.001	9.908	24.255
Linear Slope (CenterAt4)	5.800	0.328	58.674	17.656	<0.001	5.142	6.457
[Poverty2023 = 0.00]	1.968	1.213	58.017	1.622	0.110	−0.461	4.397
[Poverty2023 = 1.00]	0^b^	0					
Language Nutrition (StimQ2)^c^	0.376	0.176	58.002	2.135	<0.037	0.023	0.728

^a^Dependent Variable: Developmental Age Score in months. ^b^This parameter is set to zero because it is redundant pseudo R^2^ = 57% accounted for. ^c^For every unit increase in LN, there was a 0.367 increase in children’s language outcome.

**Table 5:** Summary of language growth models (developmental age).

Models	Information criteria/Fit		
	−2RLL	AIC	BIC
1. Intercept4 only	1712.789718	1716.789718	1724.015974
2. Intercpt4 + Slope	1682.133503	1690.133503	1704.586015
3. Intercpt4 + Slope + Gender	1680.139058	1688.139058	1702.576946
4. Intercpt4 + Slope +Poverty2023	1676.867774	1684.867774	1699.305662
5. Intercpt4 + Slope + Language Nutrition	1476.106098	1484.106098	1497.995181
6. Intercpt4 + Slope + Poverty2023 + Language Nutrition	1471.477510	1479.477510	1493.349751

Smaller information criteria = better model fit. −2RLL, −2 Restricted Log Likelihood; AIC, Akaike’s Information Criterion (AIC); BIC, Schwarz’s Bayesian Criterion (BIC).

## 4 Discussion

### 4.1 Summary of findings

The purpose of this study was to report longitudinal empirical findings for families and children associated with the implementation of KCBB over 2 years (32). Using the Family Study data, we examined the multilevel hypothesis that caregivers’/parents’ awareness, knowledge, and use of LN in interactions with their children were linked to the implementation of the KCBB intervention by the community coalition. And that children’s language acquisition was linked to caregiver’s/parent’s use of LN in the home. Findings indicated support for both hypotheses. Specific findings were:

**Figure 4:**
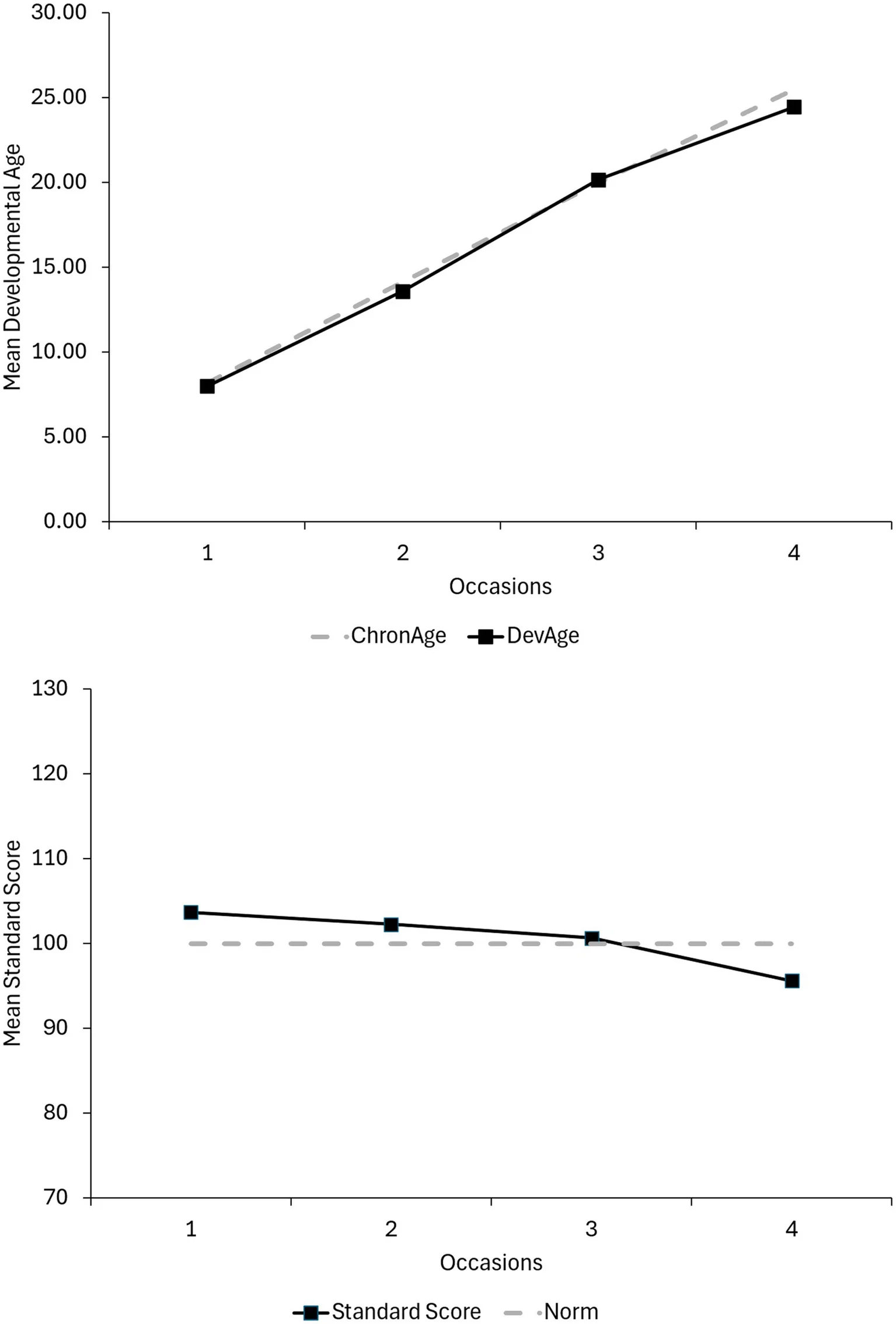
Trends in children’s early language (mean developmental age, upper panel) and (mean standard scores, lower panel). The occasions correspond to the study calendar as follows: 1 = Start (*M*chron/age = 8.2), 2 = 8 months (*M*chron/age = 14.1), 3 = 16 months (*M*chron/age = 20.0), and 4 = 24 months (*M*chron/age = 25.5).

*Finding 1*: KCBB achieved broad caregiver exposure from multiple community sectors.

*Finding 2*: Caregivers’ community experiences were associated with their LN practices at home.

*Finding 3*: LN was associated with children’s longitudinal language growth.

### 4.2 Scientific significance

These findings demonstrate through empirical associations that a multisectoral community coalition, KCBB, was able to (a) improve low-SES children’s early language experiences mediated by their caregivers/parents and (b) their language acquisition (Spanish or English) at scale in a low-SES community. Unlike traditional interventions targeting only individual children, KCBB appeared to reach the broader population through coordinated community action. Families who were generally unaware of the KCBB coalition reported receiving and applying its message-aligned guidance. This study advances language intervention research by:

- Creating a population-level intervention promoting children’s language acquisition.
- Using a manualized, theory-based approach co-created with local community partners.
- Empowering parents and community members, not outside staff, to be the drivers of change.
- Making researcher/evaluators a partnering sector within the coalition.
- Reaching a diverse, low-income population including speakers of English and Spanish with a context-specific program.
- Documenting multilevel changes through creative use of digital measures of implementation, processes, and outcomes at the community, parent, and child levels with reach and efficiency.
- Use of parent and child measures with adequate psychometric properties repeatedly over time to reduce measurement error.
- Implementing and measuring the KCBB processes and outcomes longitudinally over 2 years, not a short-term pilot.
- Building the capacity for replication of a multi-sectoral, community coalition model for achieving population-level change.

### 4.3 Limitations and future research

This study has several limitations that inform future KCBB research. First, the lack of a cluster-randomized control group limits causal inference, as available resources did not support this approach. Second, there was no true pretest of parents and children. Most families began participating after KCBB implementation was underway due to rolling family enrollment over time. Third, the sample size (*N* = 83) restricted generalizability and statistical power; larger samples would enable more complex analyses, such as latent growth modeling, to examine multiple change pathways simultaneously. The sample consisted of voluntary families interested in their child’s language, rather than a random sample.

There were several methodological considerations regarding the measures. We used online data collection to enhance efficiency and feasibility in this community-based research. A concern could be that requiring participants to have digital devices biased the sample by filtering out families without the means or technical knowledge needed. However, recent research reports that digital devices in the US are pervasive in the population (49).

Assessment relied heavily on parent report, known to introduce social desirability bias and common-method shared variance within the measurement model. We used the Community Experiences Survey, a parent/caregiver report measure, to assess the reach of the KCBB intervention’s LN messaging to residents. We used the StimQ2 to assess the depth of parent’s/caregiver’s LN application in their interactions with their children at home. We used the LENA Developmental Snapshot, a parent/caregiver-report measure, to assess their child’s language acquisition over time. This was a pragmatic solution to the challenge of multilevel repeated measurement in longitudinal research. Future studies, where feasible, should incorporate independent observations and/or direct child assessments.

Interpretation of some Community Experiences Survey scores could be strengthened in the future by specifically asking for change between now and the last assessment occasion. The LENA Developmental Snapshot provided both relativistic and idiographic growth trajectory scores for children’s language skills across the age range of interest. The StimQ2 lacked an idiographic scale of change over the age range of interest, limiting longitudinal tracking of change in the home language environment. We addressed this limitation by creating a scale based on the percentage of items endorsed in the infant and toddler forms of the measure as an intraindividual growth trajectory. Future research should employ home environment tools designed for longitudinal tracking of intraindividual change over time. Finally, while language developmental-age scores improved significantly at the same rate as typically developing children, standard scores declined slightly—not an uncommon pattern with the relativity of norm-referenced scores in young children (48). One additional limitation is that because the intervention was community-wide, families could have been exposed to language-promotion information outside of KCBB, which makes it difficult to attribute changes in LN specifically to KCBB.

This study was too short to address the central question regarding inequities in language-learning opportunities: Can improving the quality of parental interactions (LN) reduce disparities in children’s school readiness and subsequent academic achievement? Future studies with larger samples and longer follow-up surveillance can address this question and further validate the impact of KCBB across language, education, health and economic domains.

### 4.4 Contributions to public health and other outcomes

By strengthening children’s early language environments, the public health goals of prevention and public education were put in motion by the KCBB, helping to ensure individuals have the literacy capacity to thrive across contexts of life. This is because oral language is the stepping stone to many adult outcomes. Promoting newborns’ brain development, oral language, and literacy (50) advances the probability of subsequent health literacy of these children as adults (51, 52), as well as cross-over benefits in education, economic, and social outcomes.

There were several implications of the KCBB’s findings for public health. Specifically: (a) reach; it found that messages can reach families through multiple existing systems; (b) equity; it demonstrated that communities could increase children’s language-learning opportunities without requiring families to enroll in a specialized individual intervention; (c) prevention; it demonstrated that language-promotion practices can be incorporated before developmental difficulties emerge.

We argue that KCBB is particularly relevant for disadvantaged communities who are facing disparities in school readiness and limited educational opportunities and who wish to initiate their own grassroots programs. KCBB places implementation in the hands of local community leaders, tailored to local strengths and resources. Because KCBB is manualized, it may be implemented by other communities committed to improving the language and educational outcomes of their residents over time. KCBB does not require a federal system or creation of a new social entitlement program to work in communities.

The original public health framework used to guide development of KCBB targeted three levels: (a) mobilize national action, (b) engage local people in creating effective language environments, and (c) improve children’s language skills (20). The current study addressed (b) and (c), while (a) remains underutilized and its contribution to broader impact remains to be demonstrated. This study should be viewed as an early step in establishing a population health evidence base. KCBB operated at a community level, but demonstration of population-level impact requires larger samples, longer follow-up, and more robust designs. The population-level impact of KCBB will be evaluated through subsequent replications across communities, each one a building block resulting from a future national information campaign.

KCBB also appears to be an appropriate prevention intervention for use in existing universal systems of early intervention, such as those available in the United Kingdom, Australia, and most G7 countries. KCBB could be added to the mosaic of interventions envisioned within other countries’ universal systems (24).

Communities interested in replicating KCBB can access the *KCBB Community Action Planning Guide* (30), which outlines a comprehensive framework community engagement and implementation. Additional materials, including a program overview and informational video, are available on the Bridging the Word Gap Research Network website. These are available at: https://bwg.ku.edu/community-action-plan-brief/ https://youtu.be/t9yhLSQcE4Q.

## 5 Conclusion

KCBB is one of the few multisectoral interventions designed to promote community-wide change targeting LN. As such, it adds new knowledge to the literature of successful studies using this community approach to improve other public health outcomes (e.g., weight loss, youth violence reduction, etc.). An important finding for language research was preliminary evidence that this multisectoral community approach achieved broad reach to families with young children, in contrast to the individual-level approach typical of most language interventions.

A major research question in this research is whether community and parents’ LN are malleable and causal in changing children’s growth in language (see Figure 1). We were able to employ three levels of measurement of change that we could use to address this question. Within the constraints of our study design, we were able to demonstrate malleability of these variables and positive associations with child language. However, causality in this multilevel theoretical framework investigation of growth in child language skills starting from community change that influences parent/caregiver behavior change remains to be demonstrated. The KCBB approach used in future studies with stronger designs and nonparent report measures could provide the needed evidence.

### 5.1 KCBB components warranting further development

We observed uneven uptake of efforts of the 12 community sectors involved in the coalition over the course of the study. To improve dosage and strength of treatment, this implementation issue deserves additional study. Each sector completed a co-creation process in the Guide for selecting the change levers they would implement as a coalition member. The process involved ratings of importance and feasibility, leading to their final selection and implementation. This process could be made more efficient.

Encouragingly, it appears increasingly feasible to reduce disparities in language learning opportunities at the population–level, provided there is sufficient will, capacity, and resources to do it.

## Data Availability

The raw data supporting the conclusions of this article will be made available by the authors, without undue reservation.
