# Supplementary material for: Kansas City Brain Builders: associations between a multisectoral community language nutrition intervention and change in practices and growth in child language over two years
Source: Front Public Health. 2026 Oct 28;14:1986929. doi: 10.3389/fpubh.2026.1986929 (PMC13751951; doi:10.3389/fpubh.2026.1986929)
Supplement: Supplementary file 1 [file Data_Sheet_1.PDF]

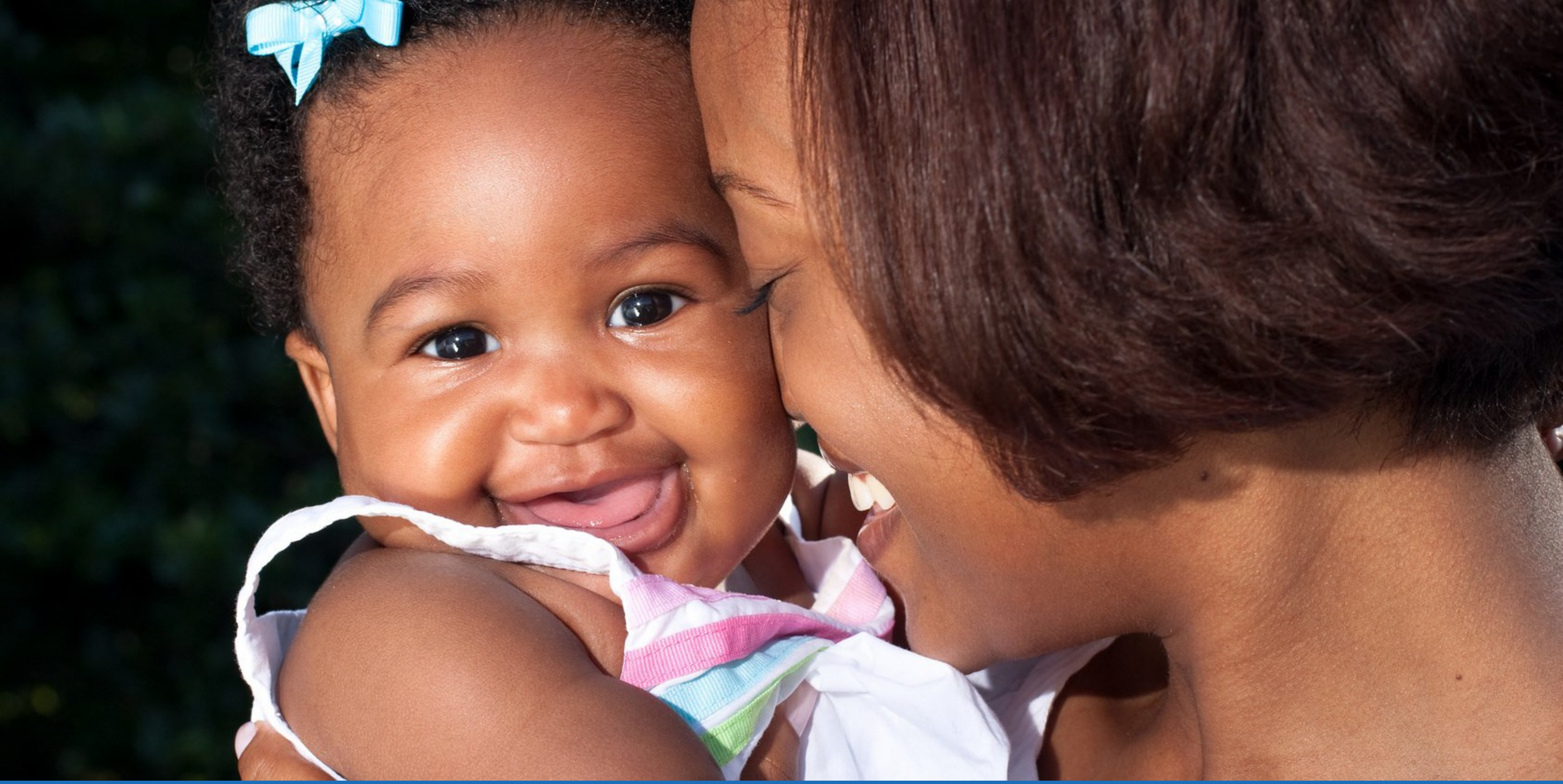

Your Community Action Planning Guide for Bridging the WORD GAP

A Resource for Community-Based Coalitions & Initiatives

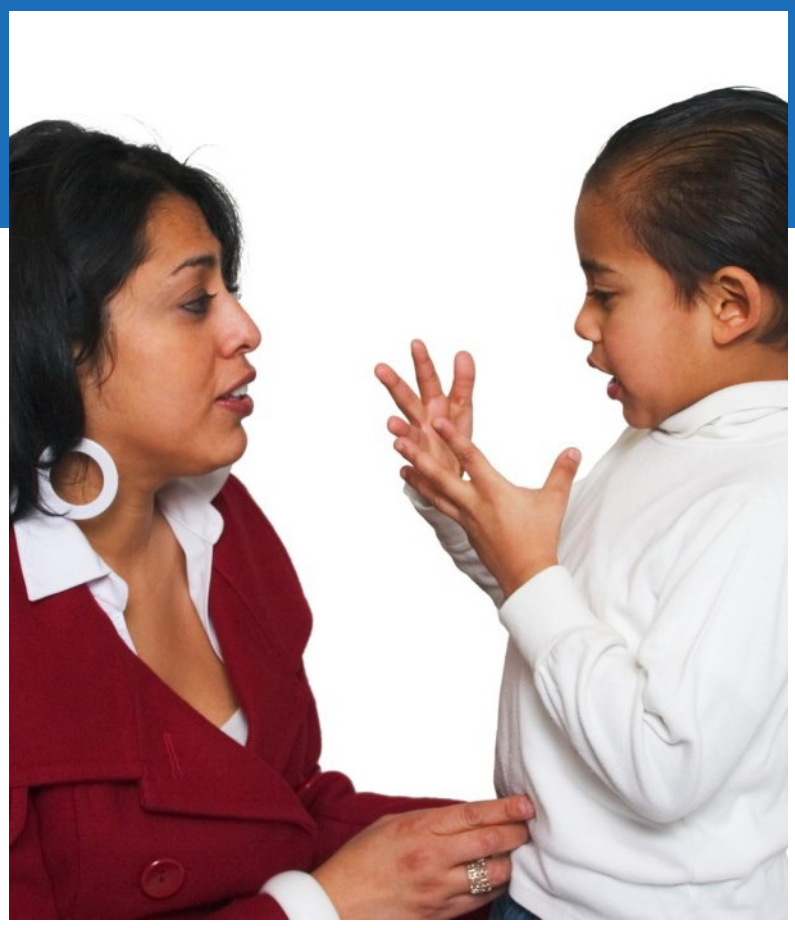

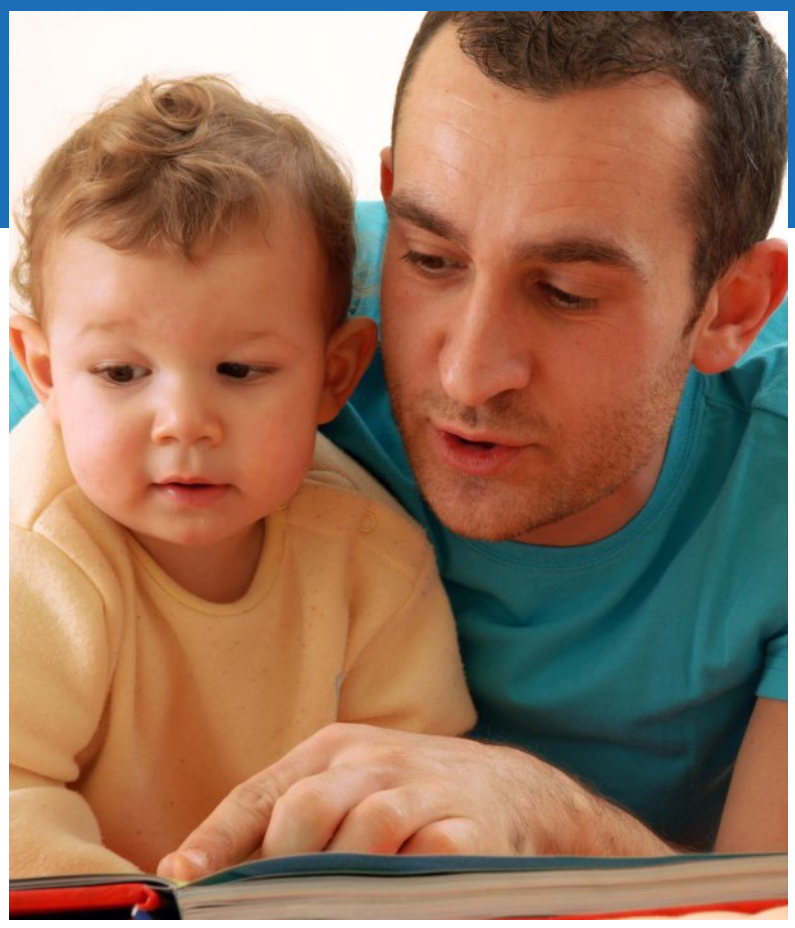

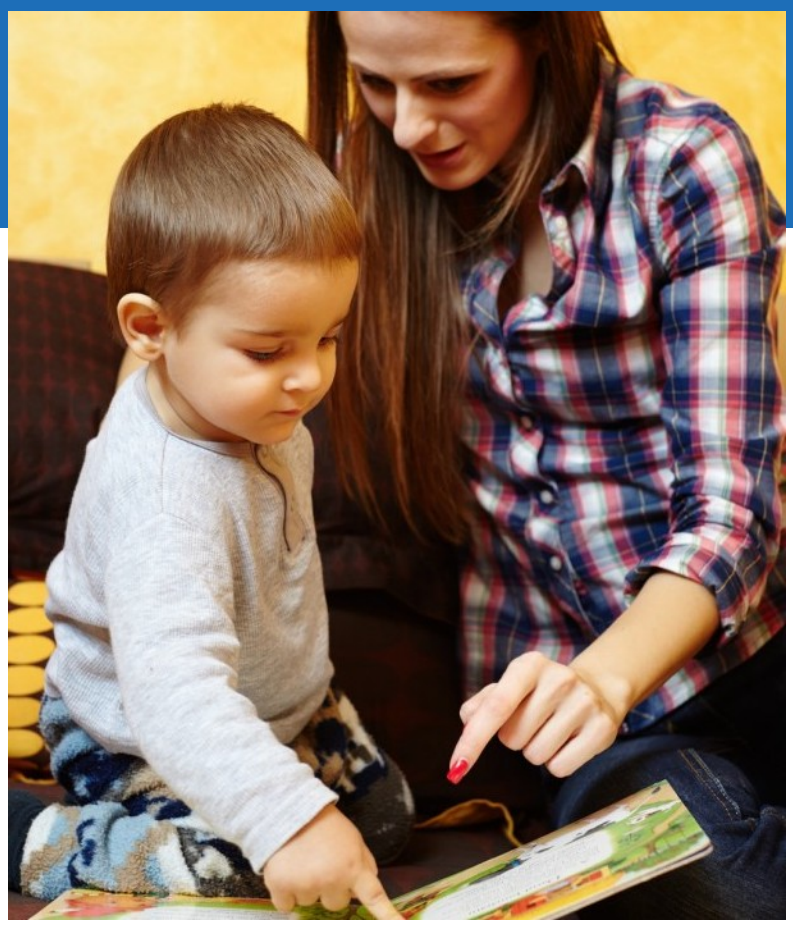

Bridging the WORD GAP National Research Network

KU Center for Community Health & Development
The University of Kansas
Life Span Institute

Development of this Action Plan was supported by the Health Resources and Services Administration (HRSA) of the U.S. Department of Health and Human Services (HHS) under cooperative agreement UA6MC27762, Bridging the Word Gap Research Network. This information or content and conclusions are those of the authors and should not be construed as the official position or policy of, nor should any endorsements be inferred by HRSA, HHS or the U.S. Government.

Charles R. Greenwood, Judith J. Carta, Dale Walker, Jomella Watson-Thompson, Alana Schnitz, Valerie Thompson, Dola Gabriel, Sanna Schneeberger, and Anna Wallisch

Bridging the Word Gap Research Network at the Juniper Gardens Children's Project and

Center for Community Health and Development, University of Kansas

2020

ABOUT BRIDGING THE WORD GAP

The mission of the Bridging the Word Gap Research Network (BWGRN) is to reduce the number of children entering school with delays in language and early literacy by preventing exposure to the Word Gap through actions of an interdisciplinary research network working to develop, promote, and scale-up evidence-based interventions so that all infants and young children enjoy rich language and literacy experiences https://bwg.ku.edu/objectives/

ABOUT THE KU CENTER FOR COMMUNITY HEALTH AND DEVELOPMENT

The Mission of the Center for Community Health and Development at the University of Kansas is: Supporting community health and development through collaborative research and evaluation, teaching and training, and technical support and capacity building. For more information on the Center, see http://communityhealth.ku.edu/.

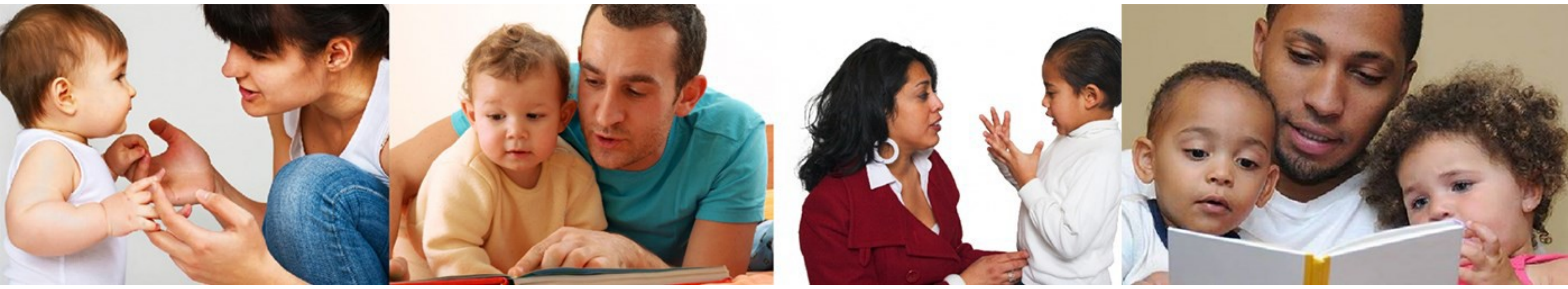

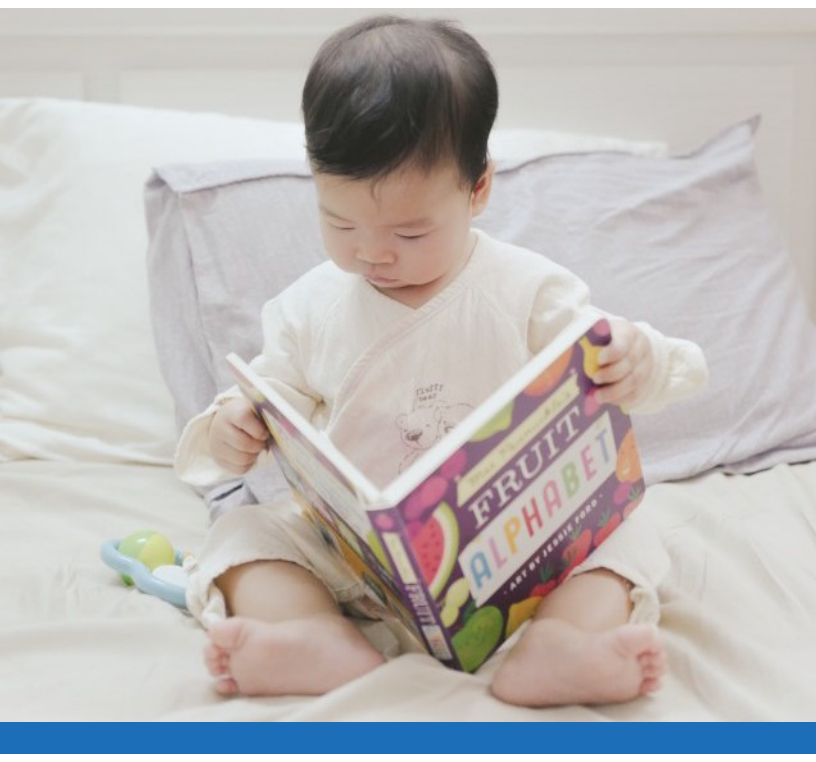

FORWARD

Almost twenty years ago, The Center for Community Health and Development began developing Community Action Planning Guides (CAPGs) as a means of facilitating collaborative change and improvement through the integration of a behavioral-community approach to planning. We conceptualized the CAPGs based on two seemingly disparate fields of study — community psychology and applied behavior analysis. Experience and expertise from both of these fields underscored the idea that if we are to change behavior, we must change the context for behavior and we must involve the community (especially those experiencing the problem being addressed) in designing strategies for behavior change. A critical aspect of these guides is the use of a public health approach to focus on societal problems that actually matter within communities.

The history of community systems improvement using Community Action Planning Guides (CAPGs) began in 1991 and 1992 with the development of a guide for Community-Based Substance Abuse Prevention, and then a second, Preventing Chronic Disease. Since then, CAPGs have been created for child abuse prevention, violence prevention, promoting healthy outcomes among youth and adults, and promoting community inclusion among persons with physical disabilities. The Center on Community Health and Development has developed, adapted, and disseminated nearly 20 versions of CAPGs to support communities in collaborating for change. The APGs provide a distillation of years of experience and research in facilitating collaborative change and improvement through the integration of a behavioral-community approach to planning. Many of the action planning guides are accessible online (http://communityhealth.ku.edu/publications/publicationsandreports).

We have found the APGs to be useful when developing community systems improvement plans, but only under certain conditions. The seven conditions for success include:

1. Having a targeted vision and mission
2. Charismatic and distributed leadership
3. Capacity building on the skills of advocacy and implementation
4. Community mobilizers
5. The presence of documentation and feedback on success changing community systems
6. An outcome that matters deeply in the community
7. Functional action plans containing all the potential new and modified programs, policies and practices that (taken together) add up to a comprehensive solution

With this right mix, you can lead an effort that will definitely make progress toward the chosen outcome. This Bridging the Word Gap CAPG incorporates these elements, and will make the work of coming together for effective action more feasible because of its systematic process for mobilizing collaborative stakeholders. Follow its guidance, and your community will be Bridging the Word Gap to improve outcomes for children and families.

Vincent Francisco, PhD, Director, Center for Community Health and Development, University of Kansas and Professor, Applied Behavioral Science, University of Kansas

The collaborative work of coming together to support change and improvements in communities is critical for addressing large-scale societal issues. Over the past 20 years, I have engaged in several community initiatives that used action planning guides to systematically bring about improvements in community-level outcomes and conditions from preventing youth violence and improving health outcomes, to neighborhood improvement. For example, my team and I worked with the Ivanhoe Neighborhood in Kansas City, Missouri through the complete community change process in this guide. Ivanhoe had experienced all aspects of urban decline lessening the quality of life for neighborhood residents. Issues this community faced included reduction in population, poverty, inadequate housing, increases in violence, and drug infiltration. With our support, several concerned residents re-invigorated the dormant Ivanhoe Neighborhood Council (INC) and worked within other neighborhood-serving organizations to improve neighborhood conditions. The process described in this guide that we used involved: information gathering, planning, developing an action planning guide, documenting progress, making outcomes matter, and evaluating improvements in desired outcomes.

Information Gathering: Through a needs assessment, community members identified housing development, crime, and youth programs as primary concerns. These findings provided the foundation for later planning efforts and the engagement of community partners.

Planning. INC collaborated with our University of Kansas (KU) Center for Community Health and Development (KU CCHD) and engaged approximately 90 residents and partners in a strategic planning phase to expound on the issues and concerns identified in the information gathering phase. This helped identify four prioritized goal areas for change: beautification, housing and economic development, crime and safety, and family and youth development/education.

Developing a Planning Guide to Facilitate Action. Through the planning process, neighborhood members identified more than 100 specific strategies or community changes from the prioritized goal areas. Neighborhood residents and partner organizations formed INC Action Teams related to the goal areas to facilitate engagement, ensure accountability, and promote partnership in implementing the strategies. The INC Action Teams used the Action Planning Guide and our *Best Change Processes* (https://ctb.ku.edu/en/best-change-processes) to mobilize the implementation of strategies identified in the Action Plan. In addition, the community established a local leadership capacity to support the work and hired a paid community mobilizer to work with the Action Teams to facilitate change.

Documenting Progress. Central to the implementation of the action plan was the use of the Community Checkbox Evaluation System to document use of the strategies and community changes. Primary documenters were community partners and the staff community mobilizer. A KU Center for Community Health and Development evaluator provided ongoing feedback on progress based on the documented activities. These data were shared quarterly to stakeholders to guide the group on current and future efforts. In all, INC facilitated 117 documented community changes across the four priority areas: beautification adult development youth development and crime and safety Documentation also permitted the evaluation of the distribution of community changes across sectors and strategies helping the community know what sectors were most engaged. For example, the data showed that the largest concentrations of community changes were being made by the faith-community (22%) and the local government (23%). Ten-years after the initial effort, 64% of the identified community change activities still were being maintained by the coalition.

Making Outcomes Matter. To institutionalize the use of the strategic plan a standard process during committee meetings was a review of the community change activities that had taken place. This helped create social contingencies of reinforcement for implementation of the strategies by the action committees. Annual recognition of neighborhood residents acknowledging their contributions also served as reinforcement. Continued implementation of community changes and resulting improvements in outcomes served as evidence of sustainability and the increased capacity of the Ivanhoe community.

Improvements in Outcomes. A **c**ritical aspect of the process was the evaluation of key outcome areas: housing improvements and crime prevention. Data on these indicators showed that the Ivanhoe Community compared very favorably to other neighborhoods in Kansas City Missouri. Ten years later, these positive results had maintained and grown over time.

This actual example of the use of the Community Action Planning process is but one that I have facilitated using action planning guides. Since the early 1990s, the KU Center for Community Health and Development has developed, adapted,

and disseminated nearly 20 action planning guides to support communities in collaborating for change. The guides provide a distillation of years of experience and research in facilitating collaborative change and improvement using the behavioral-community approach to planning and project management. Action planning guides make the work of coming together for change successful by providing a systematic process to plan and mobilize the collaborative actions of many toward common goals. These action planning guides are available online (http://communityhealth.ku.edu/publications/publicationsandreports).

I am elated by the opportunity to contribute to the development and implementation of the “Action Planning Guide for Bridging the Word Gap” (BWG-CAP). This Guide mobilizes multisector collaborative initiatives that promote rich language environments for children in their early years. These are efforts designed to provide the early language experiences to all children at risk of missing out prior to kindergarten. The Guide prompts stakeholders from multiple sectors to consider the conditions that they influence that can enable richer language engagement and interactions of children and families. For instance, what can be done in the workplace, city hall, the faith community, or healthcare to bridge the Word Gap?

The community-level efforts in which I am often engaged are related to youth violence prevention and adolescent substance abuse. Just like the Word Gap affecting many children in marginalized communities, a common underlying factor for many youth problem behaviors such as these are related to addressing socioeconomic determinants of health, including education. Through collective efforts to address the Word Gap, my hope is that communities will provide children with nurturing language learning environments, improve their school readiness, and develop long-term changes that contribute further upstream to preventing these adolescent problem behaviors.

Jomella Watson-Thompson, PhD, Associate Director for Community Participation and Research, Center for Community Health and Development Associate Professor & Director of Undergraduate Studies, Department of Applied Behavioral Science

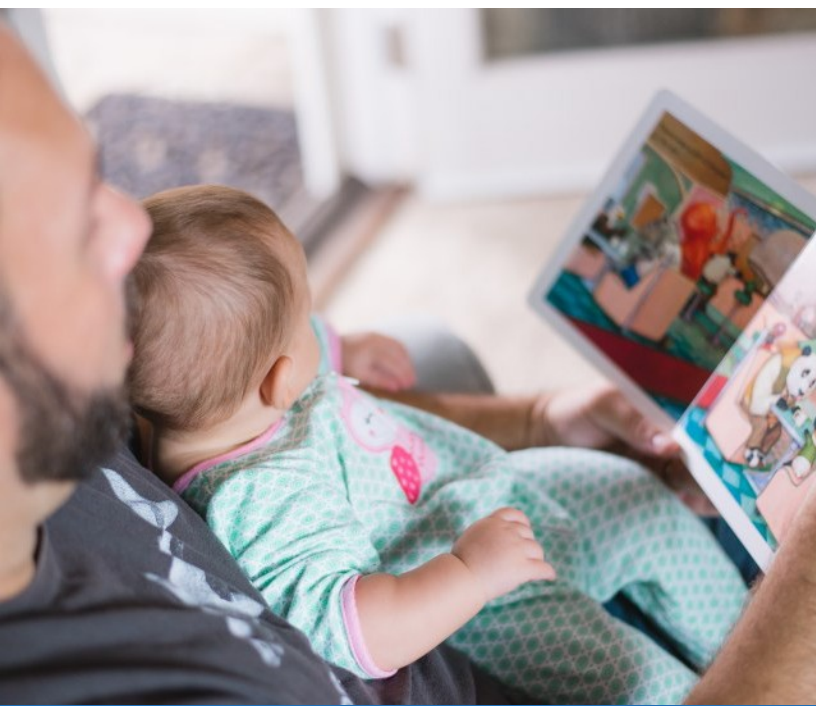

PREFACE

The history of the toxic effects of poverty on children, families, and communities in the U.S. can be seen in persisting disparities in health, education, and social outcomes that has been long-lived, often recognized as intergenerational, and defies prevention. What has been learned based on policies supporting intervention-prevention work in economically and socially disadvantaged communities is that starting younger is better and that health, education, and governmental systems need to maintain high quality to support favorable outcomes throughout out the life span. Early intervention is not an inoculation against later problems, but instead the stimulus needed for a good start and once established, needing to be promoted and maintained over the lifespan for best results.

The discovery of the Word Gap by researchers Betty Hart and Todd Risley and their team working in a poverty community in Kansas City in the 1980-90's focused on observations of the nurture and language stimulation parents actually provided their infants and toddlers. The design set out to compare contrasting groups, those in poverty vs. those more advantaged in resources, employment, and educational attainment. Their findings, repeated by others in subsequent studies, pushed the envelope on the need for language stimulation and early intervention down to birth and difference frequency, quality, and style of talk they received from the adults in the family.

This BWG Community Action Planning Guide continues this story, written by colleagues of Hart and Risley and the BWG Research Network. Coming more than 30-years later, the Guide is a wake-up call combined with a culmination of what has been learned since the initial discovery about intervening with the adults in the family, the early care, and community systems to promote a positive, rich language learning environment for each child. Also based on the success of public health prevention model, this Guide provides communities with a road map for preventing the Word Gap and its toxic effects on child development and later life outcomes for the individual, the community, and the nation.

TABLE OF CONTENTS

**FORWARD**	**3**
**PREFACE**	**6**
**TABLE OF CONTENTS**	**7**
**ACKNOWLEDGEMENTS**	**8**
**HOW TO USE THIS GUIDE**	**9**
**CHAPTER 1: INTRODUCTION TO THE PROBLEM**	**11**
**CHAPTER 2: ORGANIZING YOUR COALITION AND INFORMATION GATHERING**<br>*Planning Page: Listening to the Community*<br>*Planning Page: Documenting the Problem*<br>*Planning Page: Identifying Factors that May Affect Full Community Participation & Developing a Framework for Action*<br>*Planning Page: Becoming Aware of Local Resources and Efforts*<br>*Planning Page: Involving Key Officials and Grassroots Leaders*<br>*Planning Page: Creating a Supportive Context for Planning*	**16**
**Chapter 3. An Overview of Strategic Planning: Vision, Mission, Objectives, Strategies, and Action Plans**<br>*Planning Page: Refining Your Group's Vision, Mission, & Strategies*<br>*Planning Page: Refine Your Groups Choice of Targets & Agents of Change*<br>*Action Planning Workshop: An Example Outline*	**32**
**Chapter 4. Working Together: Involving Key Sectors of the Community**<br>*Key Community Sectors: An Example Partnership for Bridging the Word Gap*<br>*Planning Page: Choosing Community Sectors to Involve*	**41**
**Chapter 5. Preparing Your Action Plan: Identifying Community and Systems Changes**<br>*Inventory of Potential Community and Systems Changes*	**44**
**Chapter 6. Refining Your Action Plan: Building Consensus on Proposed Changes**	**64**
**Chapter 7. Finalizing Your Action Plan**	**66**
**Chapter 8. Documenting Progress and Promoting Renewal**	**68**
**Epilogue**	**73**
Glossary	**74**
**References**	**77**
**Appendix**<br>***A:** Blank Worksheet: Action Steps for Identified Changes*	**78**

ACKNOWLEDGMENTS

Preparation of this Action Planning Guide was supported by the Health Resources and Services Administration (HRSA) of the U.S. Department of Health and Human Services (HHS) under grant UA6MC 27762, Bridging the Word Gap Research Network, a cooperative agreement granted to Judith Carta, Charles Greenwood, and Dale Walker of the University of Kansas. The information or content and conclusions in this document are those of the authors. They should not be construed as the official position or policy of, nor should any endorsements be inferred by HRSA, HHS, or the U.S. Government.

The authors would like to acknowledge the collaboration that has emerged between the Bridging the Word Gap Research Network (BWGRN) and the Center for Community Health and Development (CCHD) at the University of Kansas. The collaboration has integrated the role and contribution that the community can make in preventing the deleterious effects of the Word Gap on children's language growth and cognitive development.

The first significant product was a paper laying out the vision of a multilevel, multisector, prevention program: Greenwood, C. R., Carta, J. J., Walker, D., Watson-Thompson, J., Gilkerson, J., Larson, A. L., & Schnitz, A. (2017). Conceptualizing a Public Health Prevention Intervention for Bridging the 30 Million Word Gap. Clinical Child and Family Psychology Review, 20(1), 3-24. doi:10.1007/s10567-017-0223-8.

The second was this Community Action Plan and the development of community progress monitoring tools (the Community Check Box) for a community-level effort to prevent the Word Gap. Both entities recognize the value of collaboration around BWG for both organizations going forward. Through this collaboration, we are developing the capacity to conduct effective multisector community participatory intervention research, through community partnerships that are designed to support large scale, prevention efforts to address the Word Gap.

ABOUT BRIDGING THE WORD GAP

The mission of the Bridging the Word Gap Research Network (BWGRN) is to reduce the number of children entering school with delays in language and early literacy by preventing exposure to the Word Gap through actions of an interdisciplinary research network working to develop, promote, and scale-up evidence-based interventions so that all children from birth on, enjoy rich language and literacy experiences http://www.bwgresnet.res.ku.edu/our-objectives/

ABOUT THE KU CENTER FOR COMMUNITY HEALTH AND DEVELOPMENT

The Mission of the Center for Community Health and Development at the University of Kansas is: Supporting community health and development through collaborative research and evaluation, teaching and training, and technical support and capacity building. For more information on the Center, see http://communityhealth.ku.edu/.

HOW TO USE THIS GUIDE

The purpose of this Guide is to support communities' efforts to plan and implement strategies to ensure that anywhere a child goes in the community, they receive positive and nurturing interactions with caring adults that promote their language learning.

This Guide describes a complete change process for communities wishing to provide their young children and families with the nurturing experiences they need for early language and literacy learning right from birth. This plan applies the knowledge that all parents and other caring, adults have the power to interact with their children in ways that support learning to talk. Also, this plan draws on the power that community coalitions and partnerships have in bringing together community leaders and grassroots organizations to identify priorities and take strategic actions that promote healthy child development by maintaining the positive environmental conditions that encourage young children to talk, and in a few short years be ready for school, to start reading, and to be prepared for later successful social and economic experiences.

This Guide details a full process of community change based on a record of successful community changes. The guide is designed to support the work of a local multisector collaborative partnership focused on improving the outcomes for its children and youth by creating and maintaining a healthy environment that supports learning beginning at birth and that protects very young children against potential future problems and adverse outcomes. The Guide supports collaboratively exploring and addressing key questions, such as:

- To what extent are children and families experiencing poverty and its association with educational disparity as indicated by the prevalence of low achievement, struggling readers, lack of school readiness, early language delays, school dropout?
- To what extent are community members experiencing health problems associated with poverty as indicated by rates of lack of health care or low quality maternal and child healthcare, substance abuse, crime and violence, childhood or adult obesity, or chronic health problems?
- How widespread are these problems in our community?
- Are these problems identified by community leaders, school and health officials, and community members?
- Are there current initiatives in place that address these problems and are they working?
- What are the changes that communities, parents, and caregivers of young children need to make to promote better early learning from birth for all children?
- What changes in practices, policies, and incentives should the media and business sectors make to support communities, parents and caregivers in making changes for all children?
- How can local government, civic organizations, education, and faith-based leaders play a role?

Seek to engage all sectors of the community in developing your blueprint for action.

HOW TO USE THIS GUIDE

Overview of the Guide

Chapter 1 introduces the early learning problems faced by communities with high poverty rates and a solution.

Chapter 2 provides background information on planning issues and concepts.

Chapter 3 provides an overview of the planning process, including vision, mission, objectives, and strategies.

Chapter 4 provides help determining which community sectors need to be involved in the initiative.

Chapter 5 supports planning of changes desired and strategies needed to garner full community support.

Chapter 6 provides strategies for building consensus on the changes sought.

Chapter 7 provides a process for finalizing and implementing the action plan**.**

Chapter 8 provides strategies for documenting the progress made attaining desired goals, outcomes, renewing effort as needed, and sustaining the initiative.

"Nothing happens unless something is moved."

– Albert Einstein

CHAPTER 1: INTRODUCTION TO THE PROBLEM

From one community,

"Our high poverty rate means that about 60% of our children are entering kindergarten 1-2 years behind. Although there have been steady improvements in local schools, children have not been able to fully close that educational gap. ***Our leaders need to understand why the educational gap exists and what, practically, can be done to fix it.*** *Your work on language development in low-income families and the use of strategies to address this issue would be perfect in aiding this process."*

Promoting a Healthy Community Environment for our Youngest Children

Families across America have hopes and expectations that their children will grow up to become successful, productive citizens. However, the majority of children growing up in economically disadvantaged communities find themselves confronting a range of challenges and issues in doing so. Communities with high numbers of families in poverty are challenged creating the healthy environments needed to promote children's social, educational, and economic success. All communities come with their own challenges and problems evidenced by key outcome indicators, many that can be addressed through improvements in current policies, programs, and practices. **We call on community leaders along with local policymakers, social service personnel, educators, business leaders, among others (healthcare professionals) to address their indicated problems by documenting, developing resources, assigning priorities, and promoting change.**

Why Does the Education Gap Exist?

When viewed within a lens of a lifetime, one's personal experiences along the way have much to do with resulting achievements, accomplishments, and outcomes, such as learning language, reading well, and achieving good grades, graduating high school, further education, employment, and beyond. The most rapid period of brain, language, and cognitive development occurs in the first 3-years of life (Shonkoff and Phillips 2000). Personal experiences in early childhood are the social determinants of health, education, and social well-being (Nores & Barnett, 2010; World Heath Organization, n.d.-a, n.d.-b). The social experiences that promote child development are those with stable, responsive caregiving by parents, family members, and other caregivers in the home, and community settings (e.g. child care, etc.) that occur in abundance. Nurturing conditions most occur frequently in safe, caring neighborhoods and communities that are sustained and reinforced by favorable local and national policies and resources. Such conditions are supportive of the opportunities a child has for quality child care, early learning, preschool, and education.

Conversely, children experiencing care that is infrequent, unstimulating, neglectful, or abusive may be challenged by poor language, education, and health outcomes with possible transmission for one generation to the next. Communities experiencing high levels of poverty and associated systemic disadvantage are less likely to be equipped to provide the environmental supports and resources associated with health and well being. Such conditions do not support communities in their quest to tap available regional or national resources for several reasons. At least one is residents may lack the resources needed to organize, advocate, and attain the resources needed to support quality child care, early learning, preschool, and education programs and facilities. Persons experiencing the stresses of poverty are more likely to struggle to be healthy and successful in school and later life compared to those not experiencing economic hardship. While not all children reared in poverty experience difficulties, we know that many do. Key points taken from the *State of America's 2020 Report* (Children's Defense Fund, 2020) include:

- One in six American children live in poverty, the poorest age group in the country— nearly 11.9 million children.
- Children reared in poverty are more likely to be poor at age 30 than children who never experience poverty.
- Lost productivity, worsened health and increased crime stemming from child poverty cost the nation about $700 billion dollars a year, or about 3.5 percent of GDP.
- Every year children spend in poverty is dangerous and expensive. The toxic stress of early poverty may impact children's development, creating opportunity gaps that can last a lifetime and harm the nation's economy.
- Children growing up poor are less likely to meet age/grade academic achievement goals, drop out of high school, and later become unemployed, experience economic hardship and be involved in the criminal justice system.

Thus, in the poorest communities, disproportionally high numbers of children reach adulthood without the skills and knowledge needed to succeed in today's society, as illustrated by this infographic depicting a hurdles race (Federal Reserve Bank of Minnesota, 2017). Children experiencing the stress of poverty start the race late around kindergarten, behind peers having had started before Pre-K. Poverty increases the risk of adverse learning at each hurdle (Evans & Kim, 2013).

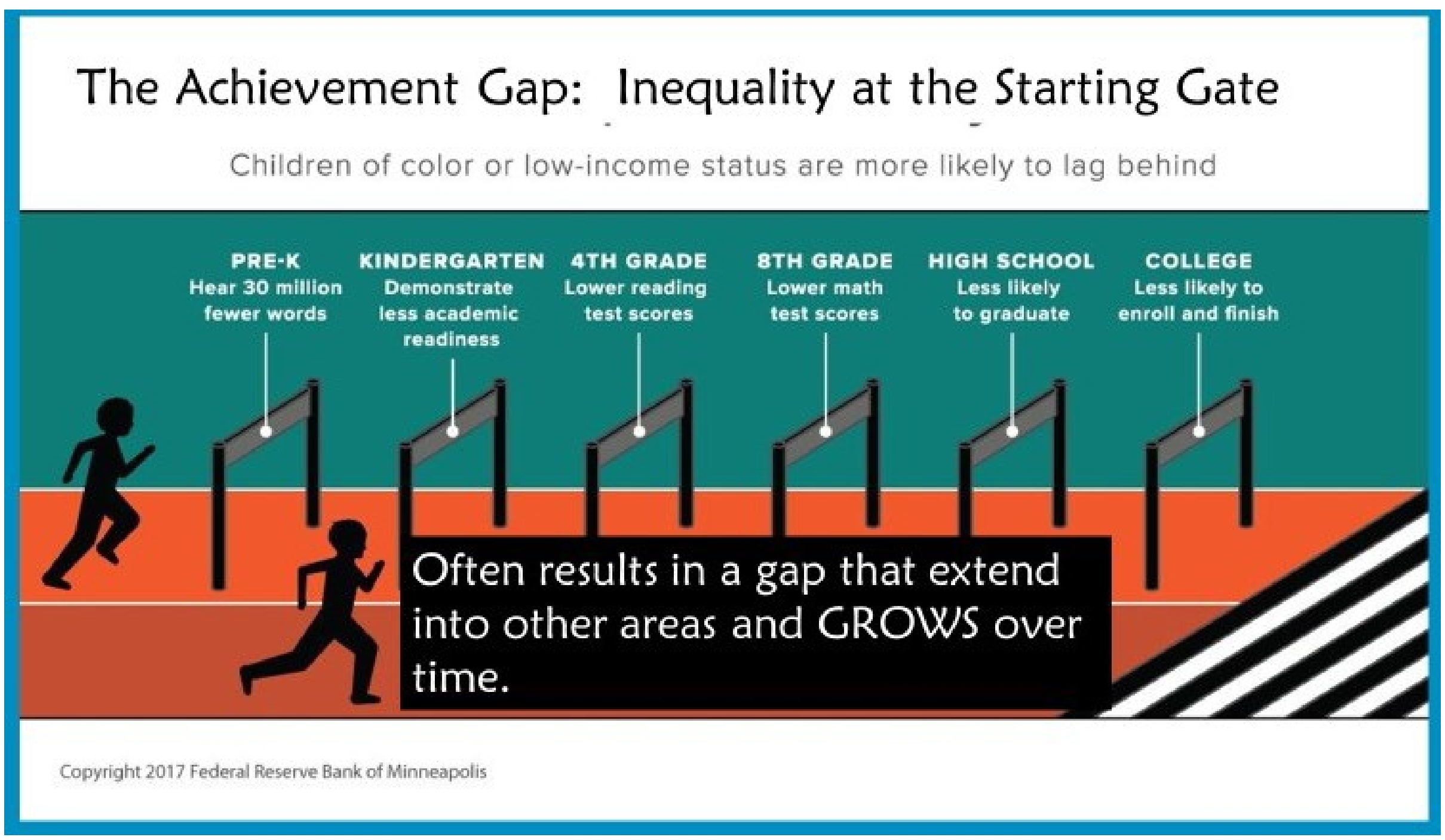

Children living in poverty on average will be less likely to experience the early language-learning experiences that fosters literacy, school readiness, and grade-level academic achievement (Hart & Risley, 1995; Hoff, 2013; Walker, Greenwood, Hart, & Carta, 1994).

Inequities in children's early learning start at birth. Poverty and its many stresses can lead to limited language learning opportunities at home. On average, children from poor families know fewer words by age three, compared to children from more advantaged families (i.e., 500 words versus to 1100 words) (Hart & Risley, 1995). These delays also are seen in children learning both English and

Spanish at home (Larson et al., 2019). The quantity and quality of language-learning opportunities that children receive from their parents and caregivers have a strong effect on children's vocabulary development by age 3. Child care, early learning, and Pre-K opportunities also play a role, where inequities exist in the access and availability of quality child care and early learning Pre-K services for children in poverty communities. Cost is often prohibitive and programs may not exist (U S. Department of Education, 2015, April).

Inequities persist in children's readiness for school. Delays in language development birth to age 5 put children at risk for not achieving expected literacy and social-emotional outcomes needed for readiness to learn once children enter kindergarten (Issacs, 2012; Neuman, Kaefer, & Pinkham, 2018; Zill & Resnick, 2006).

Inequities persist in learning to read well in elementary school. Lack of school readiness is a precursor of later achievement delays in the early grades, school failure later, and ultimately, to potential lifelong social and economic costs (Aram & Hall, 1989; Romeo et al., 2018). Some studies have documented that by the time they are entering school, children as young as 5-years-old who experience poverty score two years or more behind children from more affluent backgrounds on standardized measures of cognitive performance (Knudsen, Heckman, Cameron, & Shonkoff, 2006; Shonkoff & Phillips, 2000; Walker et al., 1994). Currently, 67% of children in the US are not reading at grade level by the time they are in the 4th grade (83% for students of lower socioeconomic status) and as a result, they have a four times greater likelihood of dropping out of high school compared to students who are proficient readers.

Income-related disparities leading to the achievement gap are now magnified because of the covid-19 pandemic. We have long been aware that children from lower-income backgrounds face a multiplicity of risks that affect their learning and long-term development. Inequities exist in higher rates of health problems, exposure to crime and violence, and limited job opportunities (Heckman, 2006; Knudsen, Heckman, Cameron, & Shonkoff, 2006).

The pandemic of 2020 has the potential to exacerbate these inequities and explode the achievement gap (Strauss, 2020, April 17). Most immediately, more families who are experiencing poverty, may face similar challenges (loss of jobs/income, food insecurity, homelessness, domestic violence, and greater health problems not withstanding covid-19). Young children may also be seriously affected by their time away from school. For children growing up in homes wherein families are experiencing extreme economic hardship or poverty, school is often the place that provides them with food, safety, and a secure, predictable place. The months spent away from school may adversely affect young children in multiple ways. But, additionally, following the pandemic, we can expect that families experiencing in poverty will face long-term economic hardships and take many more years to recover from a depression than more economically advantaged families. How all of these compound stresses affect the way parents support and interact with the youngest children during this time period may affect an entire generation and their lifetime outcomes.

What Can Be Done to Fix the Problem Using this Guide?

A child's language is at the core of everything a child does and learns at school, and is one of the most important predictors of later school success. We now know not to wait but to start promoting a child's language at birth. Hart and Risley's Word Gap discovery demonstrated that learning starts much earlier than educational research and theories of remediation had predicted. This start early message needs to get out to all in the community, because waiting until kindergarten to expect children to begin learning is simply too late. Communities need ways to support parents and caregivers in using the strategies and tools we know that can promote the language learning of babies and young children, illustrated by the those we have included in Chapter 5 below.

Pediatric and maternal health care and other community providers (e.g., home visiting, child care, foster parenting, faith-based organizations, schools, etc.) need strategies to promote the message as well as

ways that can build the capacity of parents and caregivers to provide infants and young children with rich language-learning experience that can build their language. Additional community sectors (e.g. government, media, business, philanthropy, research and evaluation, etc.) all need strategies to help provide the focus, the capacity, resources, supports and sustainability to the effort through complementary activities affecting policies, programs, and practices that support front-line caregivers' efforts across the community.

Communities need to band together to help families, schools, and communities support policies, programs, and practices that are more nurturing (Biglan, 2015) to provide the frequent language learning opportunities young children need to grow ready for preschool and kindergarten.

We are describing a proactive, prevention approach to tap the synergy between different but interrelated interventions at the community- and child-family levels focused on a common goal of bridging the Word Gap rather than only one intervention in isolation. In the community, engage and sustain the efforts by multiple sectors in collaborative partnership to build and support needed policies, programs, practices, and resources. In the child's family and child care, assist each provider in the use of evidence-based language promoting strategies. The goal is to reduce the number of children who enter school with delays in language and early literacy skills, and who go on to experience other schooling problems. What is new about this effort is, as soon as the child is born, we start setting the stage for their later success in school and beyond.

Fostering children's language stimulates brain growth by building neural pathways. Brain growth is a pronounced process during early childhood that is facilitated by early experiences, particularly language. When infants and young children hear words and have interactions with adults that are engaging, neural pathways are stimulated and connections form between what a child hears and what they experience.

When and how do we need to be talking to and interacting with children? Children learn best in their interactions with parents, family members, and other familiar adults (particularly when they are frequent, positive, and sustained). We also know that children who are dula language learners should have language-learning experiences in their home language as well as any language they are learning. Children also learn best when these interactions extend into the child care, preschool, elementary, and other educational settings.

Who needs to be talking to children? Certainly, parents are children's first teachers. The power of facilitating children's growth in interaction, words, and talk is in the hands of the parents and other caring adults in a child's life. Parents and other adults need to know that talking to children is important because when they have responsive, language-learning interactions with their children, they increase a child's capacities to learn—beginning early in their life. All adult caregivers (teachers, neighbors, other community members) also need this knowledge and how to provide children positive interactions that promote language learning.

Early Literacy and Reading? Reading is a keystone skill needed at proficiency levels well beyond basic literacy skills for success in our society (Warren 2015). Children need to be ready to learn to read at least in kindergarten and make timely progress by 3rd grade. This is important because thereafter in 4th grade, the curriculum changes from *learning to read* to a focus on *reading to learn* subject matter. Children who are still struggling readers typically do not do well in later grades.

What Informational Barriers Exist and Need to be Overcome?

- Parents may not understand the importance of talking to young children.
- Programs serving young children and their families may not understand the "Talking is Teaching" message and may not incorporate that message in their delivery of services to families.
- Community organizations and leadership may not yet know the longer-term effects of the Word Gap and the importance of talking to young children. As a result, they have yet to promote it among their

stakeholders, businesses, and service providers.

- Policies are lacking or obsolete with respect to preventing the Word Gap through resources and language-promoting messages and practices.
- Cultural practices and lack of knowledge inadvertently may discourage caregivers' promotion practices.

What Other Barriers Prevent Adults from Talking to or Interacting with Children?

- Time away from children while at work or during activities that do not include children with little or no time to talk and interact with infants and young children
- Knowing when and how to talk to an infant and toddler
- Adult absence from home and child
- Competing activities
- Lack of examples showing how to talk to or interact with babies
- Opportunities to learn parenting skills from a trusted adult

What Misconceptions Need to Be Overcome?

Many believe that children's active learning begins in kindergarten. This is not true. Active learning begins at birth.

Children's educational success is not influenced by parenting and teaching. This is not true. Tuning in and talking more to children helps build language skills (Suskind, Suskind, & Lewinter-Suskind, 2015) and can help to build important connections in the brain related to learning.

The Word Gap blames parents and families who are poor. This is also, not true. The Word Gap is the phrase used to describe the discrepancy in language-learning opportunities that some children experience—especially those already experiencing disadvantage related to poverty. To succeed, children need to read well, not just at a basic level. All parents want their children to succeed. Talking more and interacting in ways to promote language for children can be achieved in many culturally appropriate ways. All parents need to understand that they have the power to enhance their children's learning through the way they interact with their children daily.

CALL TO ACTION: We need to ensure that all infants and toddlers in our communities have the early language experiences needed to set them on a path leading to literacy, reading, and academic and social success. Making early learning in the home and child care a community priority can be a step toward promoting a healthy start, preventing the inequities and costs of stemming from the Word Gap.

CHAPTER 2: ORGANIZING YOUR COALITION AND INFORMATION GATHERING

Organizing to Make a Difference –Building a Community Leadership Team

To address the Word Gap, we cover two essential activities needed to engage a community on a solution path. This plan applies the knowledge that all parents and other caring, trusted adults have the power to interact with their children in ways that support learning to talk. The plan also draws on the power that community coalitions and partnerships have in bringing about the change needed, by joining together community leaders and grassroots organizations in planned activities that will promote and sustain the favorable positive environmental conditions needed by early learning young children. The first section covers organizing and leading a community coalition, the second section covers information gathering needed to define and identify the community problem to be addressed. This problem will serve as the basis for all other steps going forward with a solution.

Organizing and Leading a Community Coalition

The first step in forming a coalition involves leaders and leadership in building a multisector organization, *Community Leadership Team (CLT)*, with the ability to change community, policies, programs and practices, many described below, known to improve the language/literacy environment of all young children. The CLT will manage the project to prevent the Word Gap through its implementation through sustainability.

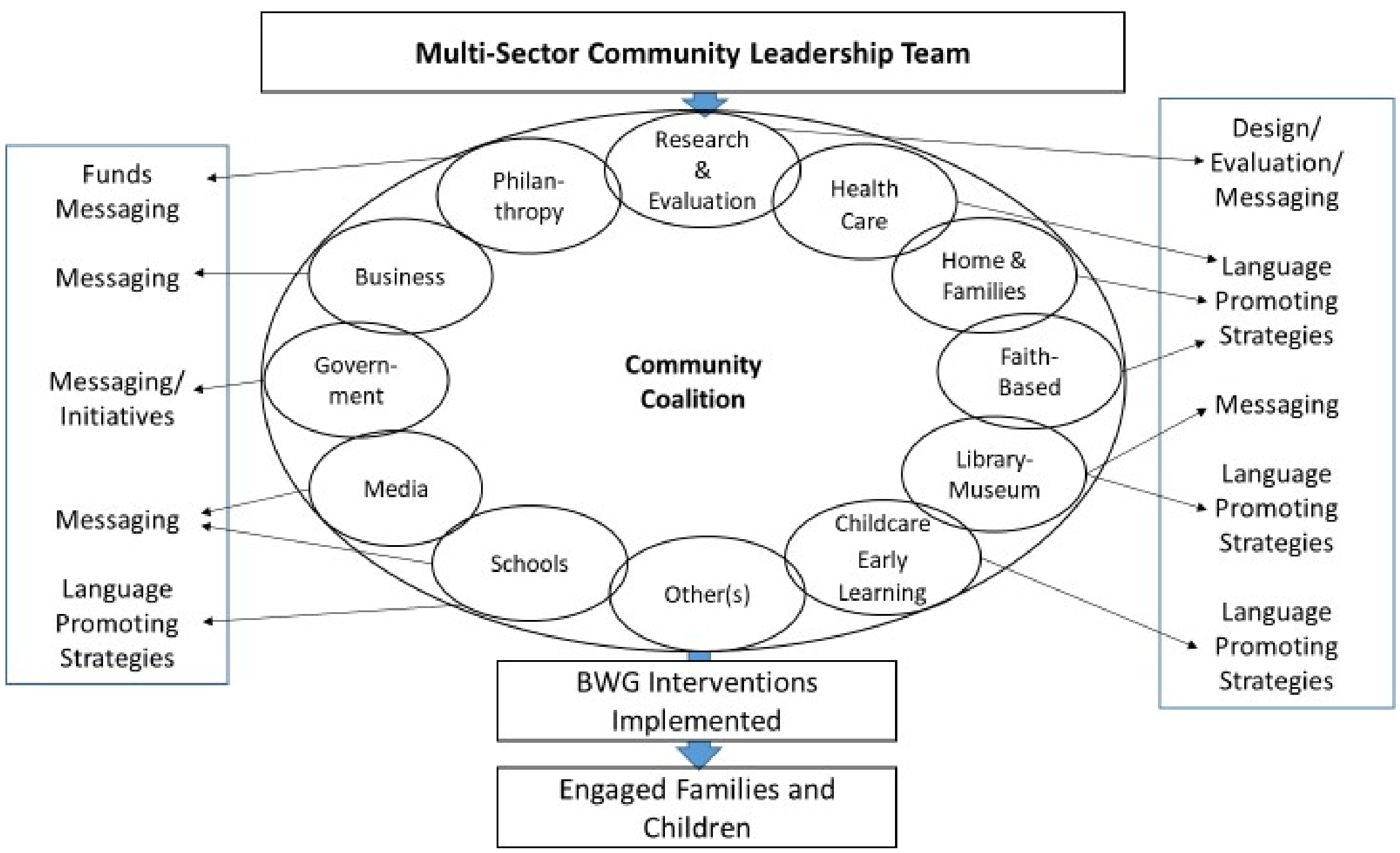

Most coalitions are initiated through efforts of at least one or more champions who bring a problem to light, envision better outcomes, see the need to act, and engage others. Champions are the founding community coalition members. Moving forward, it is essential to involve additional community leaders and representatives of grassroots organizations in the coalition when they share a concern about the problem or hold a stake in its solution.

We recommend that a CLT of stakeholders representing community sectors eventually be involved in helping organize and manage the activities designed to make beneficial changes across all sectors of the community. Under this leadership/coordination, the coalition will seek to achieve the desired community changes to prevent the Word Gap.

From its founding, we expect the CLT to grow in membership and coordinate the activities of multiple community sectors (e.g., government, child and health care, etc.) who are committed to working in partnership to achieve the vision, mission, the implementation of the strategies described in this Guide (see Figure below). Respected leaders and individuals representing a sector should be recruited to participate on the CLT agreeing to be responsible for ensuring that the goals and outcomes of their sector are targeted and implemented in coordination with the other sectors as planned. Sector leaders are also responsible for ensuring that the activities and accomplishments of the sector are documented in the context of the project's collective effort. Individual sector leaders also should work closely with their member participants to achieve the sector's contribution to the overall effort of changing policies, programs, and practices to support bridging the Word Gap as documented in this Guide and their community's Action Plan.

In brief, the CLT is expected to organize and lead implementation of a program to intervene and prevent the Word Gap using all the tools, practices, and resources available as documented in this guide as well as other resources available to the CLT. This may include for example, using the local media to produce messaging and the information campaigns needed to promote knowledge and awareness of the Word Gap and this effort taking place in the community to prevent its negative outcomes. If possible, the CLT should engage the philanthropic sector to fund local efforts and sustain them over the long run. The CLT will involve research and evaluation partners to assist in assessing and reporting progress in reaching its goals and outcomes, including help with data collection, monitoring short-term progress, and evaluating longer range impact.

For more information regarding the action planning process promoted in this Guide, visit the Community Tool Book (https://ctb.ku.edu/en). To better understand the Word Gap, resources are available through the Bridging the Word Gap Network, visit this website (https://bwg.ku.edu/).

INFORMATION GATHERING

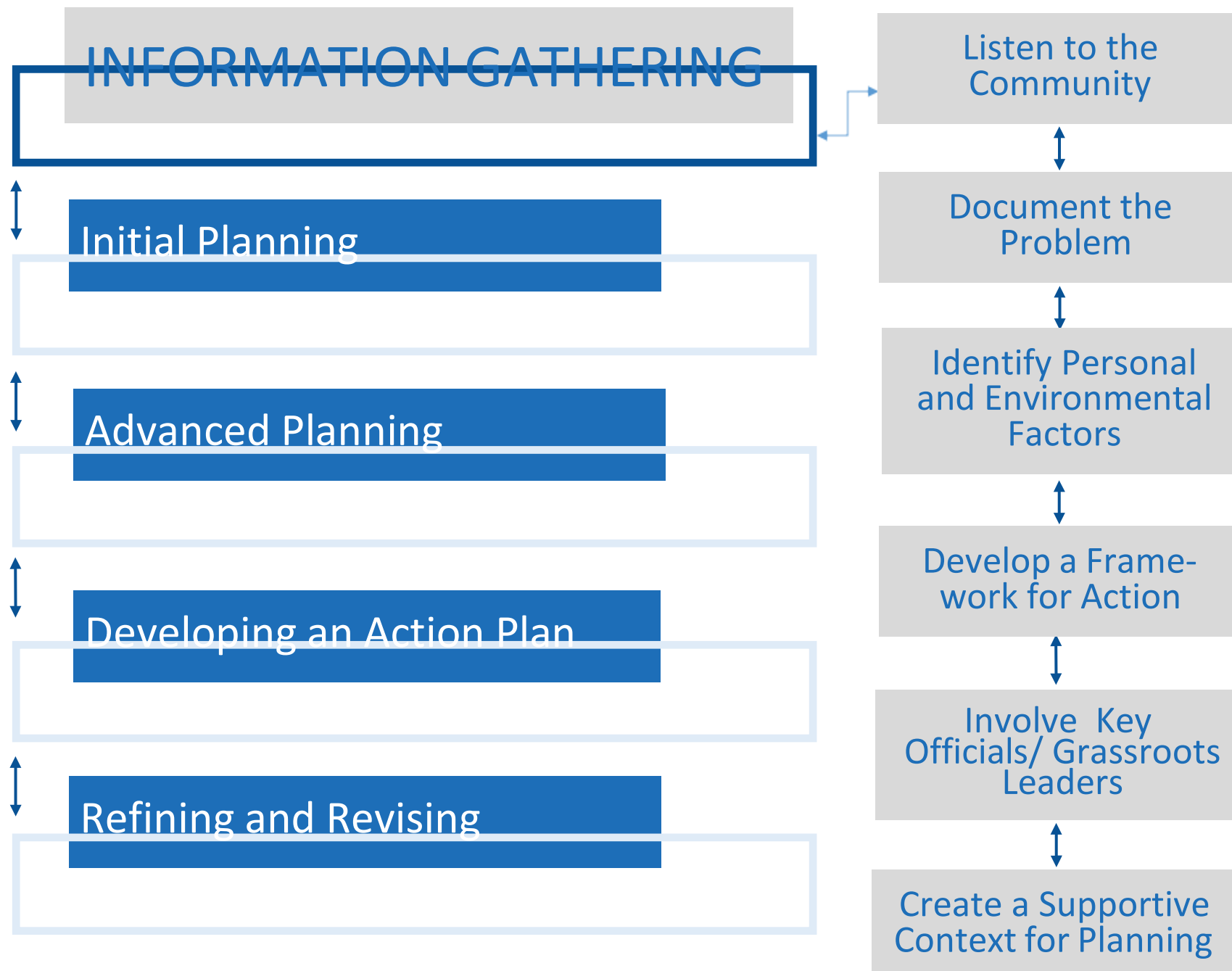

As your community works toward a broad vision of promoting language-learning experiences and school readiness for all children, creating supportive conditions requires collaborative efforts among diverse sectors of the community. Some of the sectors include health organizations, faith-based communities, schools, and businesses. Individuals who represent each sector come together to form a community coalition. Your community coalition can strive to influence systems changes (changes in programs, policies, and practices) that can enhance the community's capacity to be a healthy environment for promoting children's language-learning. A community coalition initiates its work by generating an action plan. An action plan outlines what should happen to achieve the vision for a language-promoting community. Desirable changes and proposed activities (action steps), timelines, and assignment of accountability for actions provide a detailed road map for collaborators to follow.

Listening to the Community

Listen to the community about issues and options. When listening to the community make sure you hear from all people in your community. Your team will know best how to access information from all and how to engage members of your community. It is important to remember that some community members might not have access to technology, some may have lower literacy levels and come from different language backgrounds, others may not check and respond to mailings, and may or may not feel comfortable providing feedback in a formal meeting. Consider several ways to listen to your community about perceived issues and solutions through focus groups, public forums, talking with community groups, meeting with parents at child care centers, in community centers, etc.

The key pieces of information you should gather in each listening session or focus group include:

- The perceived problem or issue
- Perceived barriers or resistance to addressing the issue
- Resources for change
- Recommend solutions and alternatives
- Current and past initiatives to address the problem or issue

Document the Problem

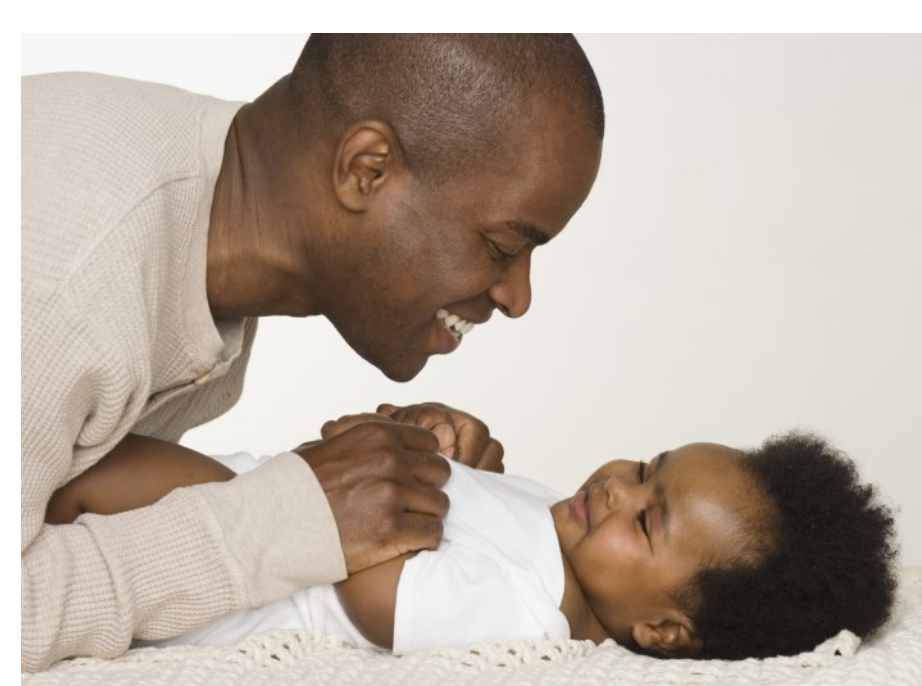

Gather data to document the problem . In addition to hearing community members' perspective on problems or goals related to the issue at hand, it is important to document the issue using existing information sources.

- "What are the issues related to the problem/topic in your community?"
- "What are the consequences of the problem?"
- "Who is affected?"
- "How are they affected?"
- "Are these issues of widespread concern?"

While the information that collected can help better understand the problem, remember that it will also play a key role in helping determine how effective the group was in addressing the problem. Use these baseline data—data that document the extent of the problem prior to implementation of the initiative—for comparison with data that document the extent of the problem after implementation of the initiative.

Listed below are helpful data sources to investigate. Please note, not all of them will be relevant to your particular issue or problem.

- School district data
- Data from specialized local, statewide, or national organizations
- State or county health department data
- State social services department data
- Hospital admissions and exit records
- Police records
- Chamber of commerce data
- Nonprofit service agency data
- Information from your local reference librarian

Also see federal websites and foundation resources such as:

- The U.S. Centers for Disease Control and Prevention's reportable disease files
- The National Center for Health Statistics' Statistical Abstract of the United States
- County Health Rankings
- Census data
- Maternal and Child Health Bureau, HRSA, Title V Block Grant Information System
- Annie E. Casey Foundation

Become aware of local resources and past and current efforts: If current efforts targeting your issue exist, think of ways in which they can become more effective via support, advocacy or other means. Consider the following:

- Do current efforts have a parallel vision? If so, can they be leveraged?
- How many people are they serving?
- Do the services meet local needs?
- What lessons can be learned from outside initiatives with a similar mission?

COMMUNITY TOOL BOX ONLINE RESOURCES

Chapter 3: Assessing Community Needs and Resources http://ctb.ku.edu/en/table of contents/assessment/assessing community needs and resources

Chapter 13: Developing a Plan for Building Leadership http://ctb.ku.edu/en/table of contents/leadership/leadership ideas/plan for building leadership/main

Chapter 4: Getting Issues on the Public Agenda http://ctb.ku.edu/en/table of contents/assessment/getting issues on the public agenda

Chapter 17: Analyzing Community Problems and Solutions http://ctb.ku.edu/en/table of contents/analyze/analyze community problems and solutions

Personal and Environmental Factors

Consider how individual characteristics of children, parents, and family members and the environments in which they live may influence how each of them might be able to access, engage, and benefit from the community-wide efforts.

Individuals most likely to affect children's outcomes include:

- Parents, guardians, and teachers
- Siblings and peers
- Neighbors and the broader community
- Service providers in health, early intervention, child care, and education.

Personal factors of children and their family members may include:

- Knowledge, skills, and history, such as temperament, exposure to caring adults, and support from family
- Degree of existing physical and psychosocial health and cognitive ability
- Educational background

- History of exposure to traumatic events

Aspects of the social and physical environment may also affect child well-being. Environmental factors may include:

- Degree of connection to family and culture
- Availability and continuity of service
- Financial barriers and resources, including incentives
- Quality of educational opportunities
- Poverty and living conditions
- Accessibility of community resources

Develop a Framework for Action

A framework (sometimes known as a "model" or "theory") helps guide the process of community action and change. How is our community to navigate the course from initial understanding and planning down the long road to bridge the Word Gap? A clear framework helps communicate the pathway for improvement, focusing local efforts on changing conditions for improved child well-being. An illustrative "Framework for Promoting Healthy Child Development" follows. Five interrelated phases include:

- Planning and building capacity (e.g., listening to the community, documenting the problem, building leadership)
- Targeted action (e.g., community organizing, contacting key officials, advocacy, social marketing)
- Community and systems change (e.g., bringing about new or modified programs, policies, and practices relevant to the mission in all appropriate sectors of the community or broader system)
- Widespread change in relevant behaviors (e.g., increasing the frequency of parents talking and interacting with their children)
- Improving children's early communication skills in the short-term, vocabulary size later, and early literacy and readiness for school even later.

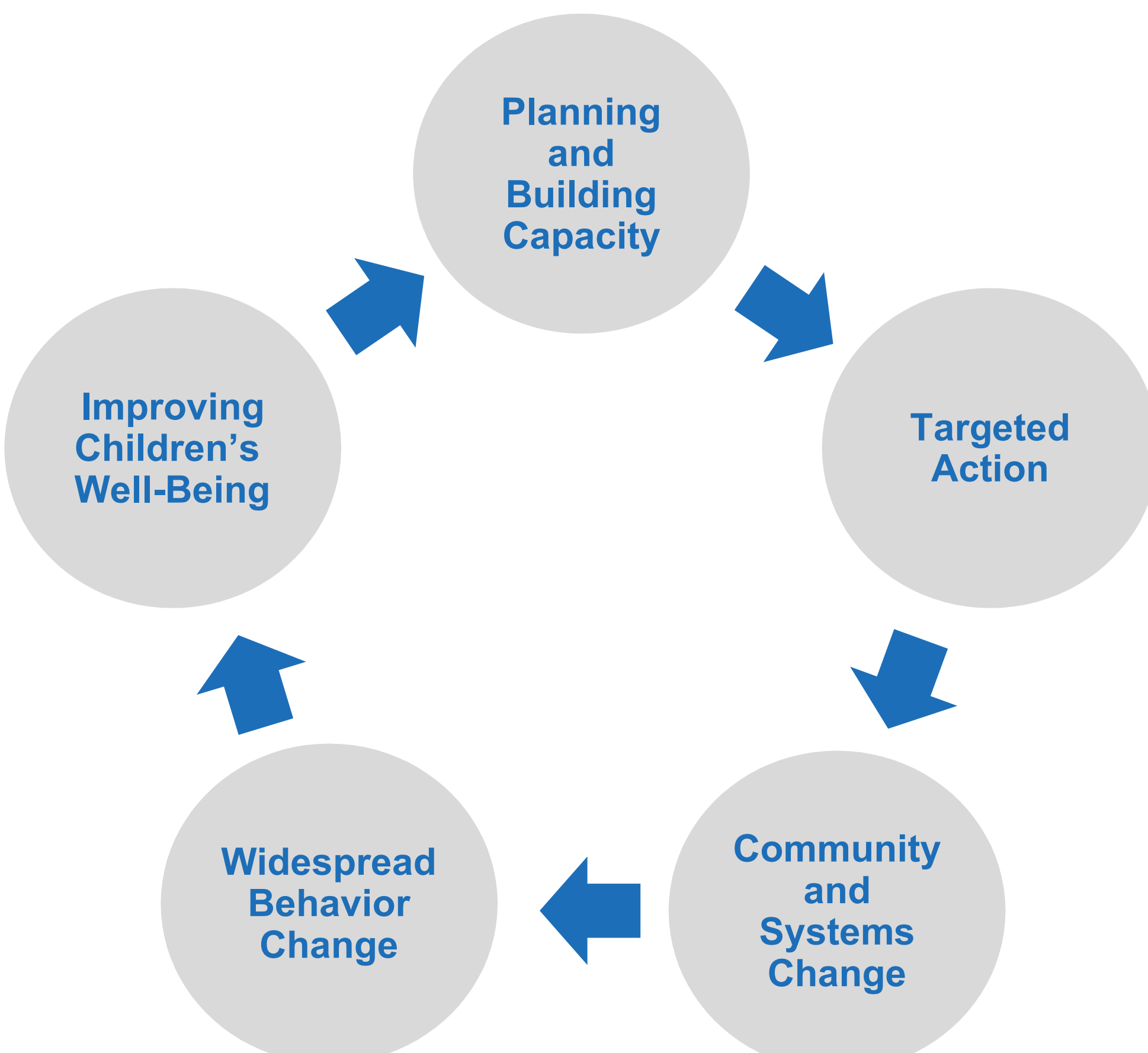

Becoming Aware of Local Resources and Efforts

You can find out about existing local resources by talking with service providers and clients. Below are questions to keep in mind. Investigating these questions is critical to ensure that the initiative doesn't overlap with ongoing initiatives or reinvent the wheel, and to maximize potential through partnering with others.

1. What programs, task forces, or coalitions with similar purposes already exist in the community?
2. How many people are involved?
3. Who are they serving?
4. Could the services and programs be more effective? How?
5. Were there past initiatives with a similar mission? Why and how did their efforts end?

Involve Key Officials/ Grassroots

Involve key officials and grassroots leaders in a planning group. While you may easily identify key officials, service providers, or representatives from relevant agencies, ensure to extend the boundaries of your planning coalition to be as inclusive as possible. Remember that your planning group should reflect the diversity of the local community.

Your group might use interviews with both key officials and key grassroots leaders to answer the following questions:

1. Who can make things happen on this issue?
2. Who are the individuals in a position to create change on a local as well as state level?
3. Who within the initiative would be most successful in getting those key officials to become involved?
4. What neighborhoods and ethnic and cultural communities are particularly affected by this issue?
5. Who are the individuals and what groups make things happen in these neighborhoods?
6. Who within the initiative would be most successful in involving members of these neighborhoods?

Creating a Supportive Context for Planning and Action

Leadership is the process by which leaders and constituents work together to bring about valued change by setting priorities and taking action. In successful groups, people take responsibility for the initiative. Leaders should have a clear vision, and the ability to attract others to that vision. Leaders also have the capacity for listening and relating to others in the group.

- The organization of the planning group is also important. The planning group must have a manageable size and structure. If many people are interested in working on the issues, the group can be structured into smaller groups, such as task forces organized by community sectors, which can report back to a coordinating council or the coalition as a whole. In larger groups or communities, action planning might initially be done in subcommittees or task groups that are organized around each sector of the community to be involved. For example, separate task forces might be set up for businesses, schools, or non-profit agencies. In smaller groups or communities, the entire group might work on the action planning. The planning groups should be diverse and integrated, including officials from various sectors. They also include people concerned about what is going on in the sector, such as youth and parents, who are affected by and interested in bringing about change.
- Community sectors should be representative of the community and include members representing diverse racial, ethnic, cultural, and linguistic background.
- A planning retreat can also be used as a time to focus specifically on the goals and means of the initiative. This can be accomplished in a half or full-day session.

Overall Tips on the Planning Process

a. *Be Inclusive*

Seek out key players with diverse viewpoints on the problem or issue, and facilitate good communication. Call on silent members during pauses in the discussion. Convey the value of each person's opinion on the issues. Occasionally, it may be necessary to discourage an overly enthusiastic member from dominating meetings. You may do so by thanking them for their comments and indicating the importance of hearing from other members of the group.

b. *Manage Conflict*

If the group is effective in attracting diverse views, conflict among members may result. Group facilitators can recognize differences, perhaps noting the diverse experiences that give rise to divergent views. To resolve conflicts, leaders may attempt to elevate the discussion to a higher level on which there may be a basis for agreement, such as, reminding the group that we are all about the same shared vision of bridging the Word Gap.

c. *Use Brainstorming Rules*

Group facilitators must avoid making judgments about ideas and suggestions. Brainstorming rules apply. All ideas should be heard and noted without criticism. Ground rules should establish that hate speech, racist, or discriminatory language are not welcome

d. *Be Efficient*

Planning meetings must be efficient, starting and ending on time. It is helpful to have an agenda and build a consensus at the beginning of the meeting about what will be accomplished and in what time frame.

e. *Communicate Products of Planning*

Planning will result in a useful product. Try to structure every planning session so that it results in a product, such as a list of issues or ideas. Show off the product at the end of planning meetings, distributing copies of the products of planning to all members.

f. *Provide Support and Encouragement*

It is important to provide support and encouragement throughout the process of planning. Good planning takes time; it usually requires months to produce a detailed plan of action. Acknowledge the contributions of all participants, especially key leaders. Let the group know when it is doing a good job.

“The goal is to turn data into information and information into insight”

– Carly Fiorina

PLANNING PAGE: LISTENING TO THE COMMUNITY

Please review the ideas in this chapter. Use these planning pages to conduct listening sessions in the community. This will help refine your group's understanding of the problem or issue, barriers and resistance to addressing the concern, resources for change, and recommended alternatives and solutions. Your group might arrange town meetings or focus groups in which to consider these issues.

The Problem or Issue

1. What are the issues related to Bridging the Word Gap in your community?
2. What are the consequences of these issues?
3. Who is affected?
4. How are they affected?
5. Are these issues of widespread concern?

Barriers and Resistance

1. Are there key individuals or groups who might oppose your efforts?
2. Can they be involved effectively?
3. What other barriers might limit the effectiveness of the initiative?
4. How can the barriers and resistance be overcome?

PLANNING PAGE (cont.)

Resources for Change

1. What resources and capacities are needed to address the mission?
2. Who are the local individuals or groups who can contribute?
3. What financial resources and materials are needed?
4. Where might the money and materials be obtained?

**RESOURCES NEEDED**	**POTENTIAL/EXISTING SOURCES**
People/Organizations:	
Financial:	
Materials:	

Solutions and Alternatives

1. What are some alternatives for addressing the problem or goal in light of the anticipated barriers and resources? These ideas may provide an initial indication of what solutions might be acceptable to the community. (The group will refine these ideas in its action plan that is described in later chapters of this guide).

PLANNING PAGE: DOCUMENTING THE PROBLEM

Please review the ideas in this chapter. Use this planning page to focus your group's efforts to document data related to healthy child development.

Community Data on Poverty-linked risks to Healthy Child Development

Various systems have data from behavioral surveys regarding poverty rates and associated risks to healthy child development. Ask questions of importance to your community.

1. What is the poverty rate in the Community?

a. Consider locating the Community's data to the zip code (five and/or nine digits) or school catchment area whenever possible using data from the sources such as the latest US Census, and poverty support programs (e.g., SNAP [food stamps], TANF, etc.).

b. Document the percentage of children under age 6 living in families with incomes at or below the federal poverty line.

2. What are the community's school-based, educational outcomes based on multiple sources (e.g., school district records, state education department, annual report cards, media reports)?

a. High school graduation rate?

b. Elementary school 3rd or 4th and 8th grade reading proficiency rate?

c. School readiness rate for children prior to or beginning of Kindergarten?

d. Rate of 3- and 4-year-old children expelled from child care or early education programs?

e. Rate of language delays in young children (e.g., referrals to Part C-IDEA infant/toddler programs based on language delays)?

f. Rate of 3- and 4-year-old children enrolled in center-based early education programs? (or rate of children not served by any early child program?)

3. What are the community's adult and child health outcomes based on multiple sources (e.g., public health department, city, county and state health agencies, etc.)

PLANNING PAGE: IDENTIFYING RISK/PROTECTIVE FACTORS & DEVELOPING A FRAMEWORK FOR ACTION

Use this planning page to refine your understanding of factors that may affect the mission of Bridging the Word Gap. Outline the framework for action (or "model" or "theory" of change) that your community initiative will use to address its mission. Your group might use past planning products, interviews with community leaders and outside experts, and models provided by others (refer to the illustrative "Framework for Promoting Healthy Child Development").

Here's a resource for personal and environmental factors affecting early child development:
https://www.childtrends.org/wp-content/uploads/2017/03/2016-61ConstructsMeasureChildWellbeing.pdf

Some Factors that May Affect Bridging the Word Gap

Various systems have data from behavioral surveys regarding Bridging the Word Gap. Ask questions of importance to your community. Here are some examples:

1. What personal factors affect healthy child development? These may include parent or caregiver parenting knowledge and skills, parent/caregiver responsiveness and warmth, and shared family activities/routines.
2. What environmental factors affect healthy child development? These may include protective factors such as stimulating and safe home environments and cohesive neighborhoods and safe housing; risk factors may include hazards related to healthy births, poverty, infant injuries, and limited access to medical/dental care.
3. How can we use this analysis – and our experience and knowledge of our local communities – to identify promising strategies and tactics for promoting healthy child development?
4. Are there existing services and supports in place in the community?

Framework for Action

1. Is the community initiative already using a framework for action (or model or theory of practice) to guide its efforts?
2. If appropriate, how can it be adapted?
3. What is the logical path from initial understanding and planning to improvements in outcomes at the community level?
4. How can this framework for action help guide the group's efforts?

PLANNING PAGE: BECOMING AWARE OF LOCAL RESOURCES AND EFFORTS

Please review the ideas in this chapter. Use this planning page to refine your group's understanding of existing programs and resources as well as current and past efforts of groups with a similar mission.

Your group might use interviews with community leaders to help with these questions. Informants might be drawn from key officials in health and human services, and the business and government sectors, as well as parents and grassroots leaders in communities particularly affected by the concern.

Existing Programs and Resources

1. What are the existing programs and resources for promoting bridging the Word Gap?

2. How many youth and families are they serving?

3. Do these services and programs meet community needs?

4. Can these services be made more effective?

Current and Past Initiatives

1. Are there coalitions currently involved in promoting Bridging the Word Gap?

2. If so, who are they?

3. How many people are actively involved?

4. Are these groups as effective as they could be?

5. Were there past initiatives with a similar mission?

6. Why and how did their efforts end?

PLANNING PAGE: INVOLVING KEY OFFICIALS AND GRASSROOTS LEADERS

Please review the ideas in this chapter. Use this planning page to refine your group's understanding of which key officials and grassroots groups should be involved in the initiative.

Your group might use interviews with community leaders to help with these questions. Informants might be drawn from key officials in health and human services and the business and government sectors, as well as parents and leaders in communities particularly affected.

Key Officials

1. Who can make things happen on this issue?
2. Who are the individuals in a position to create change?
3. What contact people from the initiative would be most successful in getting these key officials to become involved in the initiative?
4. Consider involving those who may initially be for (and against) the initiative.

Key Officials to be Involved: | **Contact People from the Initiative:**

Key Grassroots Leaders

1. What neighborhoods, racial, ethnic, cultural, and linguistic communities are particularly affected by this concern?
2. Who are the individuals and what groups make things happen in these neighborhoods communities?
3. Which contact people from the initiative would be most successful in involving members of these neighborhoods and communities?

Key Grassroots Leaders: | **Contact People from the Initiative:**

PLANNING PAGE: CREATING A SUPPORT CONTEXT FOR PLANNING

Use these planning pages to consider how your group will position itself for success. Note the leadership of the planning group and its preferred size and structure, organization, and plans for integration of key leaders and people affected by the concern.

Leadership

1. Has a person or small group accepted responsibility for the initiative's success?
2. How can the leaders attract others to the vision?
3. How can the leaders enhance their skills to relate to others?
4. How can the group select for and support those with the attributes necessary to help transform the community?

Group Size and Structure

1. What is a manageable size for the planning group?
2. If more people wish to be involved, what structure will be used to include them? (Perhaps the planning group might be composed of a smaller executive or steering committee that would report to the group).

PLANNING PAGE (cont.)

Group Organization

1. How will the planning group be organized? In larger groups or communities, planning might initially be done in subcommittees or task groups organized around community sectors, such as schools or religious organizations.

Group Diversity and Integration

1. How will diversity and integration of differing perspectives be assured?

2. How will influential people be involved?

3. How will other people affected by the concern be involved?

4. How can the continuing participation of those with resources and authority be maintained while preserving the involvement of other community members without official titles?

CHAPTER 3: STRATEGIC PLANNING

INITIAL PLANNING

The purpose of this chapter is to provide an overview of strategic planning which is the process by which a group defines its vision, mission, objectives, strategies, and action plans. When groups develop a plan for action, they decide what they hope to accomplish and how they will do so to get there. This chapter will help the group consider where to direct its efforts, determine who's at risk, and decide who's in a position to help bridge the Word Gap by identifying targets and agents of change.

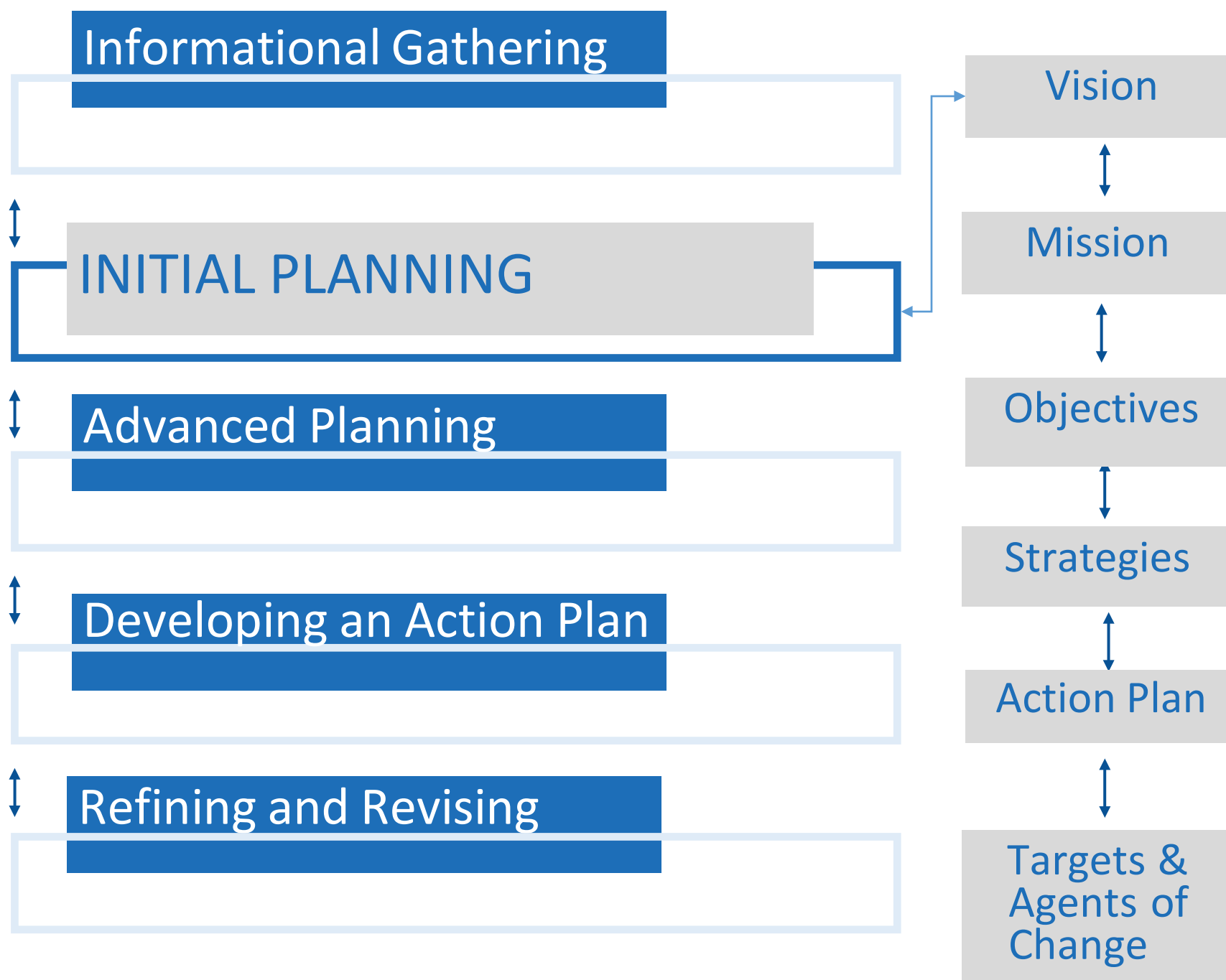

Strategic Planning. A strategic plan consists of five components: vision, mission, objectives, strategies, and an action plan.

Vision

The vision should convey the community's ideal condition desired for the future. It should be shared by members of the community, uplifting to those involved in the effort, and easy to communicate (i.e., "All children and families thrive.")

Mission

The mission describes what the group is going to do and how. The mission might refer to implementing a community-based prevention program to reduce the Word Gap; or increasing the level of a positive condition, such as increasing the percentage of children who enter kindergarten ready to learn. The mission may look something like this: "To build a community supportive of positive language and learning outcomes for all children through policy, system, and practice changes."

VMOSA: A planning process to develop a blueprint for moving from dreams to action steps to outcomes.

Vision ○ **Mission** ○ **Objectives** ○ **Strategies** ○ **Action Planning**

For additional guidance, see Community Tool Box Chapter 8, Section 1: http://ctb.ku.edu/en/table-ofcontents/structure/strategic-planning/vmosa/main

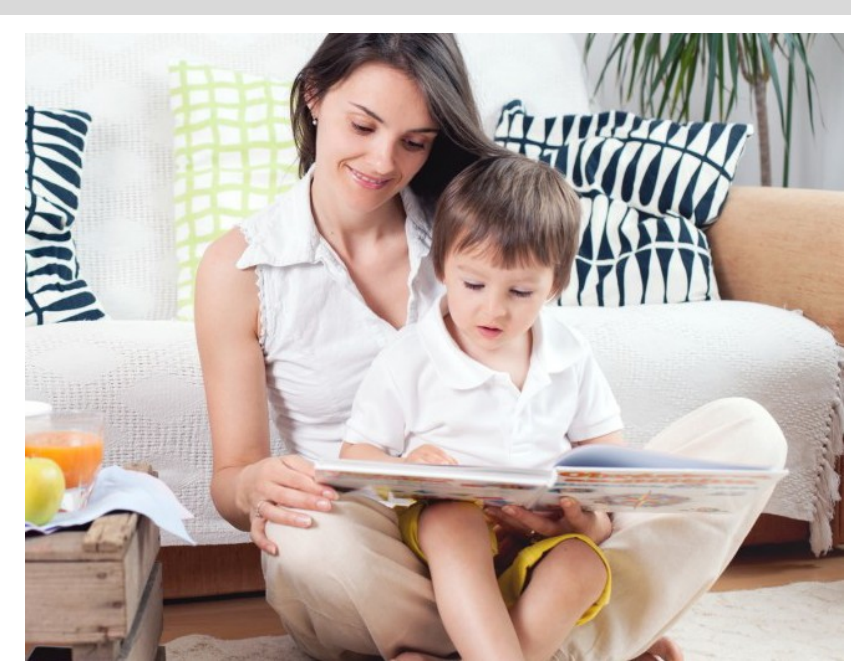

Objectives

Objectives refer to specific measurable results of the initiative. They include: a) key behavioral outcomes (e.g., 90% of all children will enter kindergarten ready to learn, increasing infant's and toddler's scores on progress monitoring measures of language); b) related community level outcomes, (e.g., improving the quality of language interactions in childcare settings, all parents in NICU having knowledge and skills to support their infants' language development, increasing the resources in libraries to support the message of early literacy for infants and toddlers); and c) key aspects of the process, such as adopting a comprehensive plan for the bridging the Word Gap coalition. Objectives set specified levels of change and dates by when change will occur.

Example objectives

a. By the year 2030, increase by 30% the percentage of children who enter kindergarten with the language skills needed to succeed in school.
b. By the year 2030, decrease by 30% the percentage of children entering kindergarten who are significantly below average in their expressive language skills (skills needed for success in school).

The objectives must be measurable (within the limits of the measurement systems now or potentially available), challenging, important to the mission, timed, and feasible to accomplish.

For more information on developing objectives, see the Community Tool Box Chapter 8, Section 3:
http://ctb.ku.edu/en/table-of-contents/structure/strategic-planning/create-objectives/main

Strategies

Strategies refer to how the initiative will be conducted. Types of strategies include building coalitions among community groups and organizations, organizing grassroots efforts at the neighborhood level, modifying access to products and services, and advocating for new programs, policies, and laws related to the mission. A group may use a variety of strategies to meet its objectives and fulfill its mission. The proposed strategies should be consistent with what is known about planned change with communities, organizations, and individual behavior.

Although your group's mission, objectives, and strategies may be mandated, its action plan will reflect your community's unique vision, goals, concerns, and experiences.

Some example strategies

- Use the media to promote public awareness of the benefits of positive adult interaction with children.
- Build a successful community coalition that involves all relevant sectors of the community in promoting child development.
- Enhance grassroots involvement in children's initiatives.
- Promote coordination and integration of existing services and resources for promoting bridging the Word Gap.
- Advocate for changes in programs and policies related to creating healthy environments for children.

Visualizing Healthy Environments to Develop Strategies

- Policies supporting parental leave time
- Improved pay for early educators
- Improved quality of child care
- Readily available resources on the initiative found in school and libraries
- Policy support importance of children's home language while learning another language

For more information on developing strategies, refer to the Community Tool Box Chapter 8, Section 4 for more information:
https://ctb.ku.edu/en/table-of-contents/structure/strategic-planning/develop-strategies/main

Action Plans

Action plans describe how strategies will be implemented. They include action steps for community and systems changes to be sought. Action steps indicate what actions will be taken (what), the responsible agents (by whom), the timeline (by when), resources and support needed and available, potential barriers or resistance, and with whom communications should occur. Example community and systems changes and action steps for identified changes are provided in later chapters.

For further information on developing action plans, see the Community Tool Box Chapter 8, Section 5:
http://ctb.ku.edu/en/table-of-contents/structure/strategic-planning/develop-action-plans/main

Targets and Agents of Change

When the group has determined where it is going and how it is going to get there, it will focus on key persons whose behaviors need to be changed and those who are in a position to affect change. Clarifying who is the recipient of the change efforts to address the problem will help in planning for action.

TARGETS OF CHANGE

Potential targets of change include children, their parents, caregivers, and teachers who have been identified as being at risk for poor language and school outcomes or other target populations identified. They also include those who parents trust in providing information about their young children and their development. These may include: pediatricians, nurses, public health nurses, social workers, librarians, and others in the community.

AGENTS OF CHANGE

Potential agents of change include all those in a position to contribute to the Word Gap solution, such as parent and extended family members, early educators, pediatricians, nurses, public health workers, nutritionists, librarians, media experts, teachers, and marketers. They also include those who have a responsibility to contribute to the solution, including business people, religious leaders, and elected and appointed officials.

For further information on identifying targets and agents of change, please refer to Chapter 8, Section 3 of the Community Tool Box:
https://ctb.ku.edu/en/table-of-contents/analyze/where-to-start/identify-targets-and-agents-of-change/main

"If you don't know where you are going, you'll end up someplace else."
– Yogi Berra, former New York Yankees catcher

PLANNING PAGE: REFINIING YOUR GROUP'S VISION, MISSION, AND STRATEGIES

Use these planning pages to refine your group's vision, mission, objectives, and strategies.

Vision

1. The vision describes the ideal condition desired for the community. It conveys the community's dream for the future and "why" the effort exists. It must be a shared vision; uplifting and easy to communicate. Please state the vision of your group.

Mission

1. The mission statement describes the special task or purpose of the group. It communicates "what" the group intends to do and "why". It must be concise, outcome-oriented, and inclusive. Please state the mission of your group.

PLANNING PAGE (cont.)

Objectives

1. Objectives state the goals toward which project activities are directed. Objectives describe "how much" will be accomplished in specific measurable results and state the time-frame for accomplishments. Objectives must be challenging, important, potentially measurable, and feasible to accomplish. Please list the objectives of your group, inserting the appropriate dates and target percentages:

   By the year _____, the percentage of 2 year old children who demonstrate age-appropriate expressive language on developmental screening measures will increase by______percent.

   By the year_____, the percentage of 2 year old children who fall below the cut-off score for typical expressive language development on developmental screens will decrease by _____ percent.

   By________, ____

   ____

   ____

   By________, ____

   ____

   ____

   By________, ____

   ____

   ____

PLANNING PAGE (cont.)

1. Strategies describe “how: the objectives are going to be met.

Broad strategies include: increasing community cohesion, increasing personal experience and competence, decreasing environmental stressors and barriers, and enhancing environmental support and resources.

Specific strategies related to changing individual behavior include: a) providing information and enhancing skills, b) enhancing services and supports, c) modifying access, barriers, and opportunities, d) changing consequences (e.g., altering incentives and disincentives), and e) modifying policies and practices.

Specific strategies related to organizational and community development include: a) community assessment and monitoring, b) public awareness and media campaigns, c) integrating and coordinating local agencies and resources, d) coalition building, e) modifying access to products and services, f) developing resources to enhance family and peer support, g) advocacy and nonviolent protest, h) enforcement of existing policies and laws, and i) changing policies and laws.

PLANNING PAGE: TARGETS & AGENTS OF CHANGE

Use these planning pages to refine your group's vision, mission, objectives, and strategies. Please note that if you are applying for grant funds, the funding agent may largely or fully predetermine the mission, objectives, and/or strategies.

Targets of change

1. Targets of change are those who directly experience the problem or are at risk, or those people who contribute to the problem through their actions or lack of action. Please list the identified targets of change for your group.

Agents of Change

1. Agents of change are those who are in the best position to contribute to the solution and those who have a responsibility to contribute to the solution. Please list the agents of change identified for your group. Remember that targets of change can also be included as agents of change.

PLANNING PAGE: STRATEGIC PLANING WORKSHOP

An effective strategic planning session allows a diverse group of participants to:

- Clarify common purpose—Through listening, gathering and reviewing data, and building a shared vision and mission.
- Generate and critique options—Through consideration of risk and protective factors, broad and specific strategies, and the community's framework for action, it identifies particular changes in communities and systems (i.e., new or modified programs, policies, and practices) to be sought to achieve the mission.
- Obtain consensus about community and systems changes to be sought—Methods can include ballot voting about the importance and feasibility of proposed changes, or having participants use "dots" to register preferences for changes to be sought.
- Decide how to proceed as a group—Through open discussion, the group identifies action steps (i.e., who will do what by when) to bring about the identified changes.

Background work before the session/workshop:

- Listening sessions with a variety of people including those most affected.
- Documenting the issues or problems, including data on the priority levels of problems or goal attainment.

Products

- Product of Session/Day One: A new (or renewed) statement of the group's vision, mission, objectives, and strategies. (These may require review or approval by a broader group).
- Product of Session/Day Two: A set of community and systems changes (i.e., new or modified programs, policies and practices to be sought in each relevant sector of the community (e.g., Community Organizations, Faith Communities, Government).

Homework Before Session/Day Two

Review the "Inventory of Potential Community and Systems Changes for Bridging the Word Gap" in chapter 4. **Bring recommended changes to be sought**:

a) By specific strategy (i.e., providing information and enhancing skills; enhancing services and support; modifying access, barriers, and opportunities; changing consequences; and modifying policies)

b) By community sector (e.g., community organizations, businesses, government).

PLANNING PAGE (cont.)

Example Planning Schedule: Keep in mind to format should tailor to each community including a virtual format

Session One (1/2 Day)

8:30 Continental breakfast, registration

9:00 Welcome and Introductions

9:20 Overview of the Strategic Planning Process

9:30 What is a vision, mission, objective, strategy, and action plan?

9:45 Vision: Creating your own community's vision

10:15 Mission: What are we trying to accomplish and why? Stating your mission.

10:45 Objectives: How much of what will we accomplish by when? Creating your objectives.

11:15 Strategies: How will we get there: Identifying a set of broad and specific strategies.

12:15 Questions/ Wrap Up: Group summarizes accomplishments of the day

12:30 Adjourn

Session Two (1/2 Day)

9:00 Review of Session/Day one and overview of Session/Day two

9:15 Identifying Targets and Agents of Changes: Who should benefit? Who can contribute?

9:30 Identifying community and systems changes by strategy (work in small groups organized by strategy)

10:15 Small Group Reports

10:45 Identifying community and systems changes by sector (work in small groups organized by sector)

11:15 Small Group Reports

11:45 Building consensus on community and systems changes to be sought (e.g., vote using dot stickers or post it notes placed on large sheets of paper around the room, each featuring a potential change to be sought)

12:15 Next Steps:

Building consensus/seeking approval from the larger group (if appropriate)

Identifying Action Steps for each change to be sought (who will do what by when)

Plan for documenting progress and promoting celebration and renewal

12:45 Questions/ Wrap Up: Group summarizes accomplishments of Session/ Day Two

1:00 Adjourn

CHAPTER 4: INVOLVING KEY SECTORS OF THE COMMUNITY

ADVANCED PLANNING

It is vital to involve a variety of community sectors in the prevention initiative. Community sectors are the parts of the community which will help the group fulfill its mission (e.g., education, faith communities, social service, government, businesses, etc.). Some sectors will be selected because they provide a good way to reach families while other sectors will be included because they offer a way to involve community members who have an interest or responsibility in bridging the Word Gap.

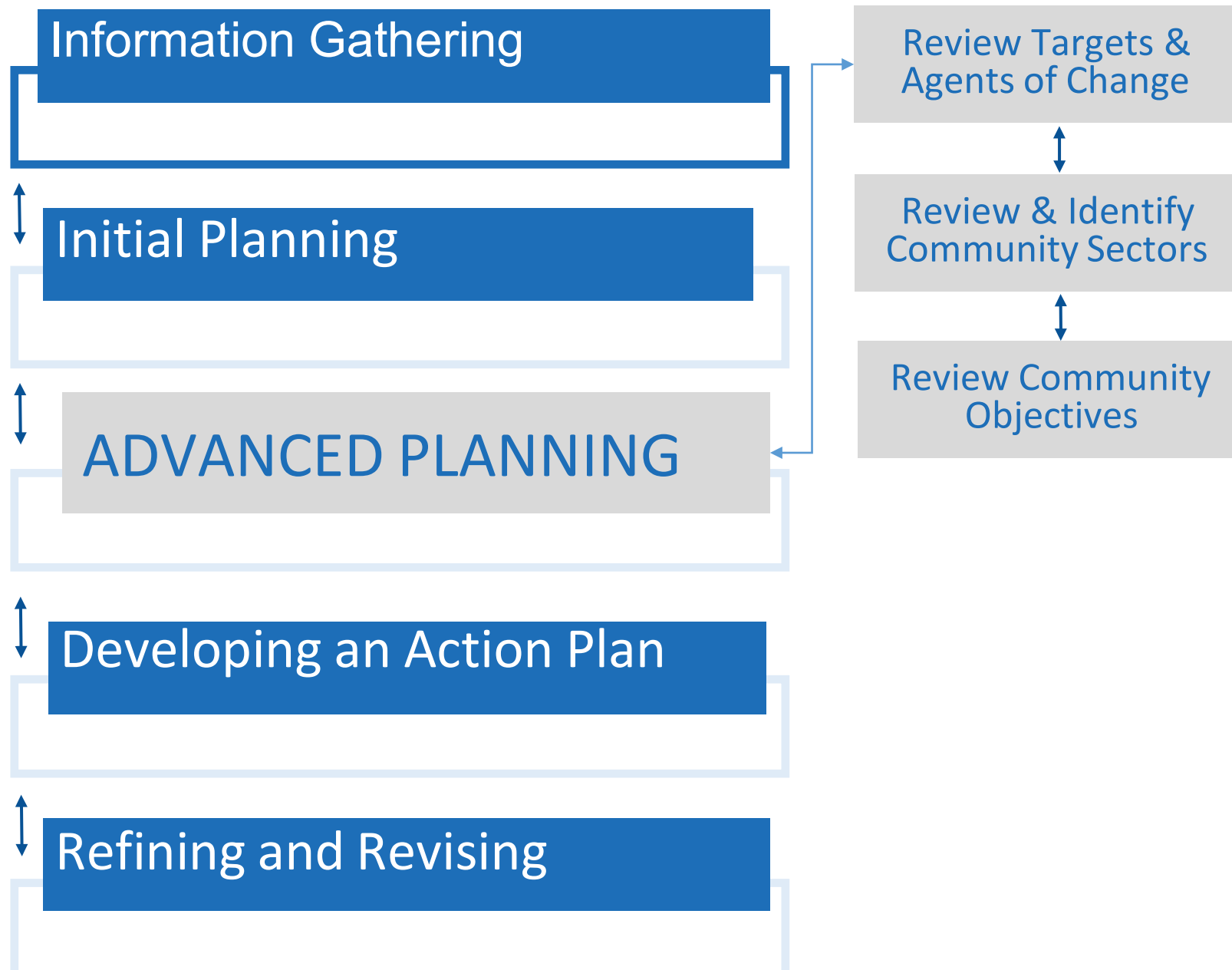

Important Activities

1. Review the targets and agents of change identified in the previous chapter. These are the people whom your group hopes to influence and involve in its efforts.
2. Review the diagram on the next page of the community sectors involved in an example of a Bridging the Word Gap coalition. Modify the chosen sectors to fit your community's unique needs, resources, barriers, and experiences.
3. Use the planning page at the end of this chapter to identify the sectors that your group will engage. Each sector should help engage your targets and agents of change. Your initiative's own particular sectors will reflect the overall vision, mission, objectives, and strategies, as well as local resources and opportunities.

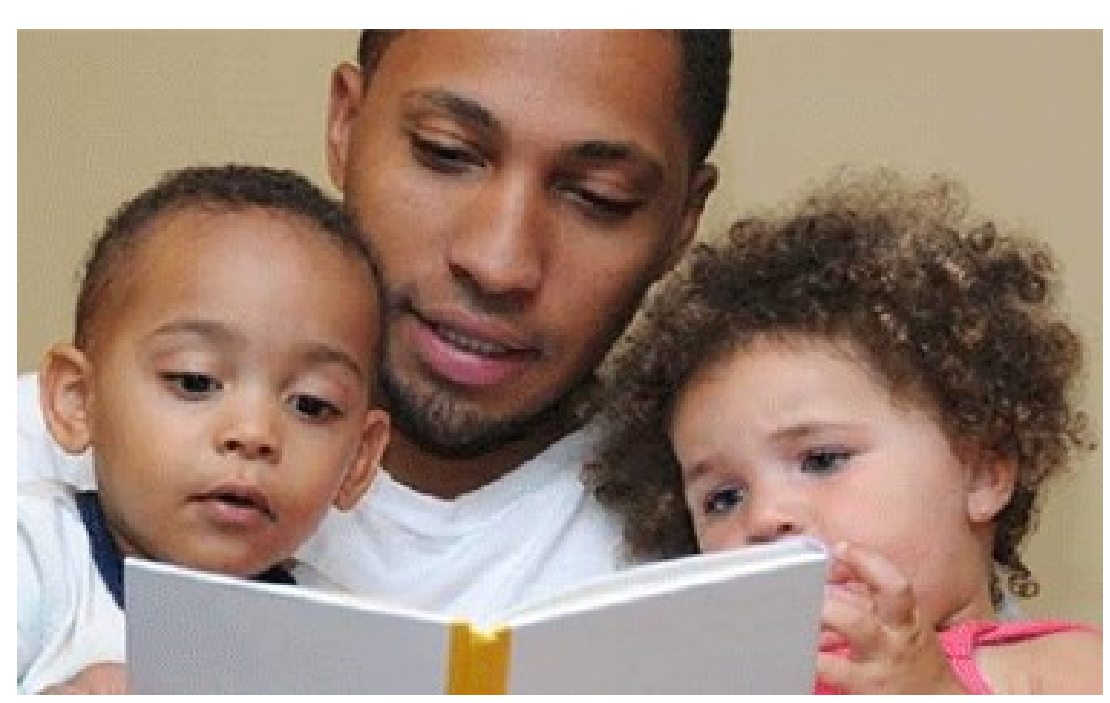

Key Community Sectors

Here is an example of community sectors or groups that might be involved to fulfill the mission of Bridging the Word Gap. Which community sectors should be engaged to address our mission? Can you think of other types of community groups that have a concern about Bridging the Word Gap?

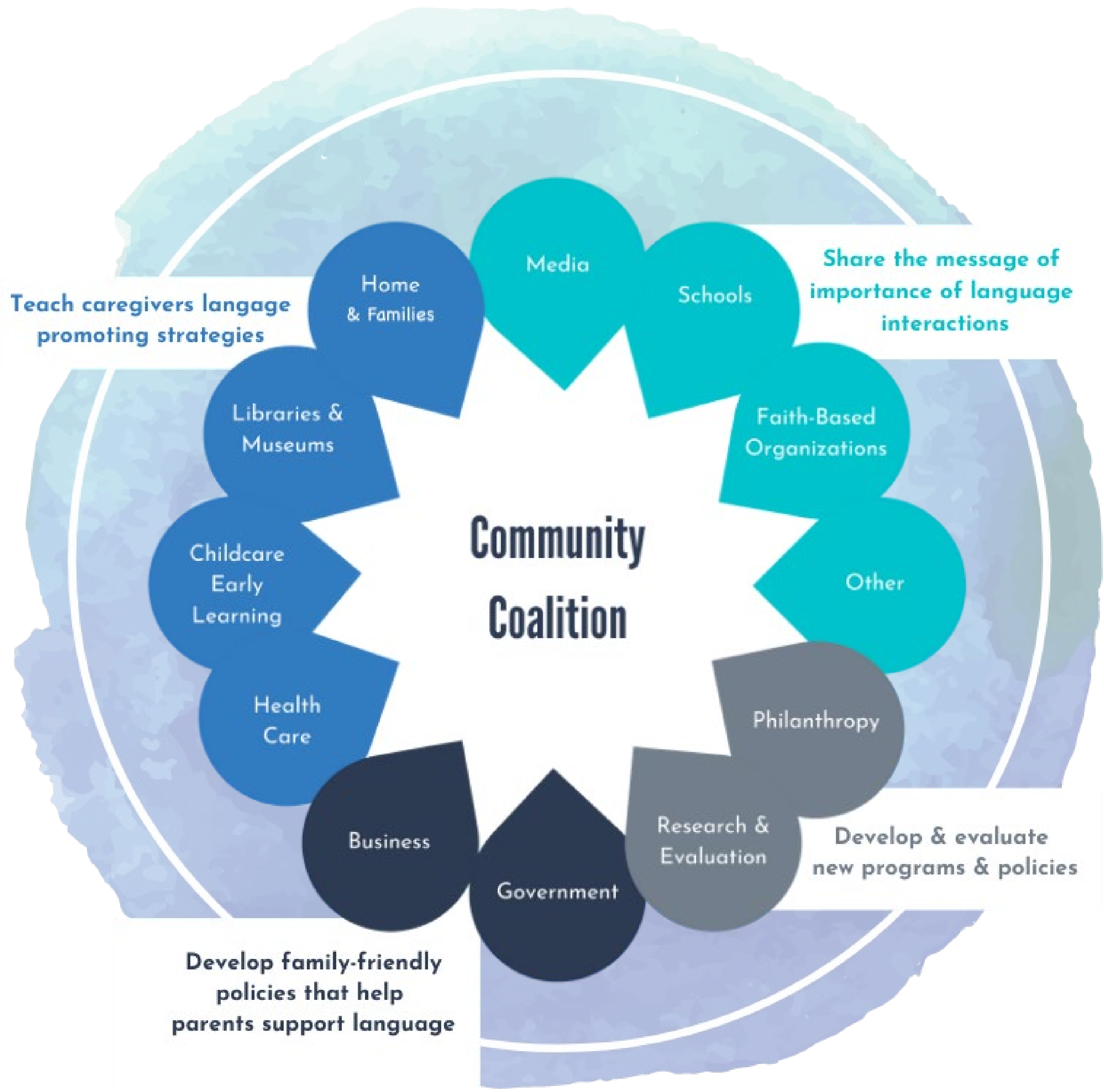

PLANNING PAGE: CHOOSING COMMUNITY SECTORS TO INVOLVE

Review the diagram on the previous page. Use this page to list proposed sectors of the community in which your group can and will have influence. Consider what community sectors will best enable the group to reach the targets of change and to involve potential agents of change. Consider the following questions: Does the sector provide a way to reach large numbers of families who would benefit from the initiative (e.g., families experiencing economic disadvantage, poverty) who are at increased risk? Does it provide access to community members who have an interest or responsibility for bridging the Word Gap? Is this part of the community important to the mission? What other sectors could or should be involved?

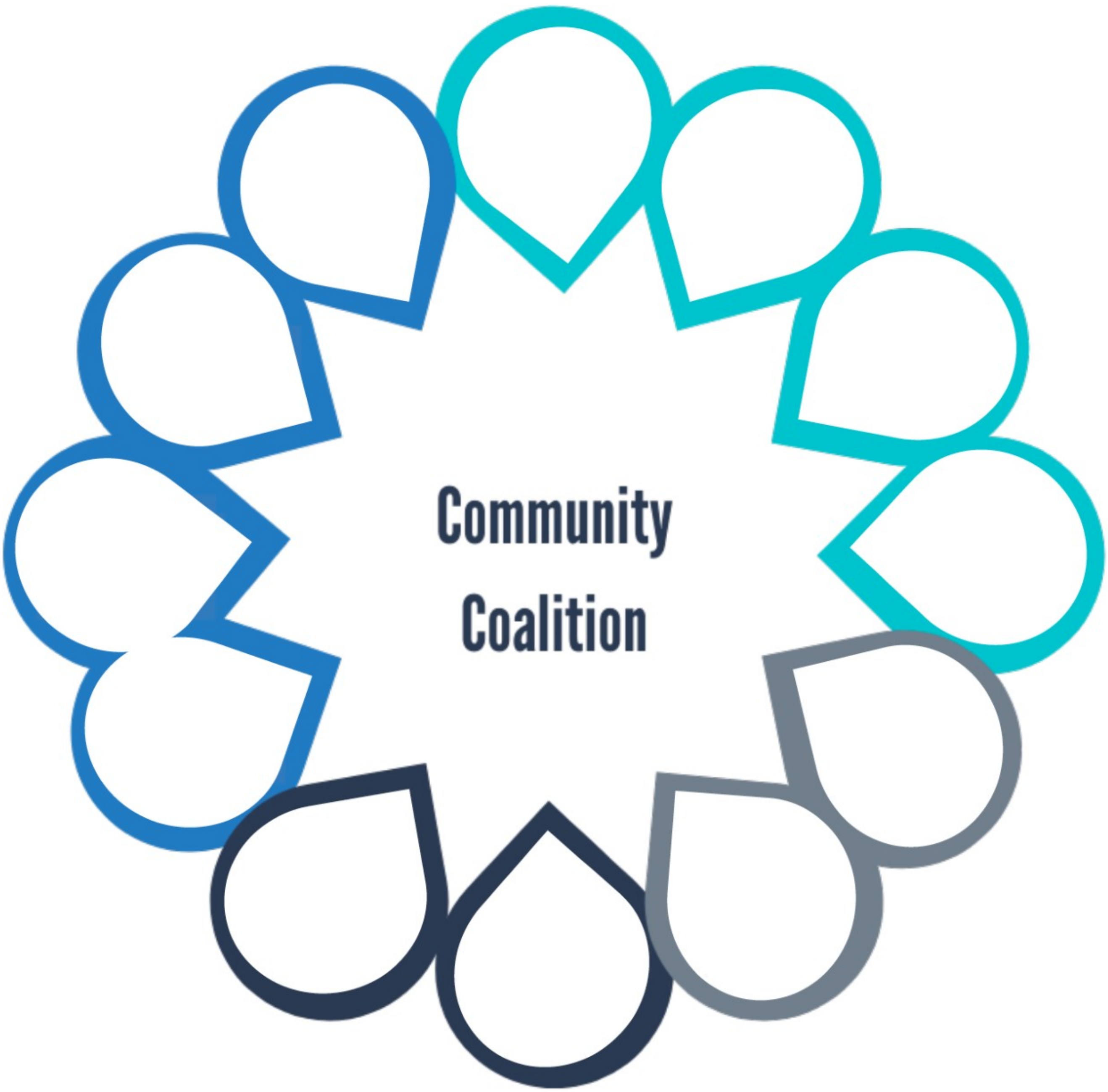

CHAPTER 5: IDENTIFYING COMMUNITY & SYSTEMS CHANGES

DEVELOPING AN ACTION PLAN

The purpose of this chapter is to help choose community changes that your group will seek in each sector of the community. Your group might change programs, policies, and practices to address the mission. This chapter provides an inventory of possible changes that your group might choose in each sector. Ultimate decisions about what changes or improvements will rest with your community group.

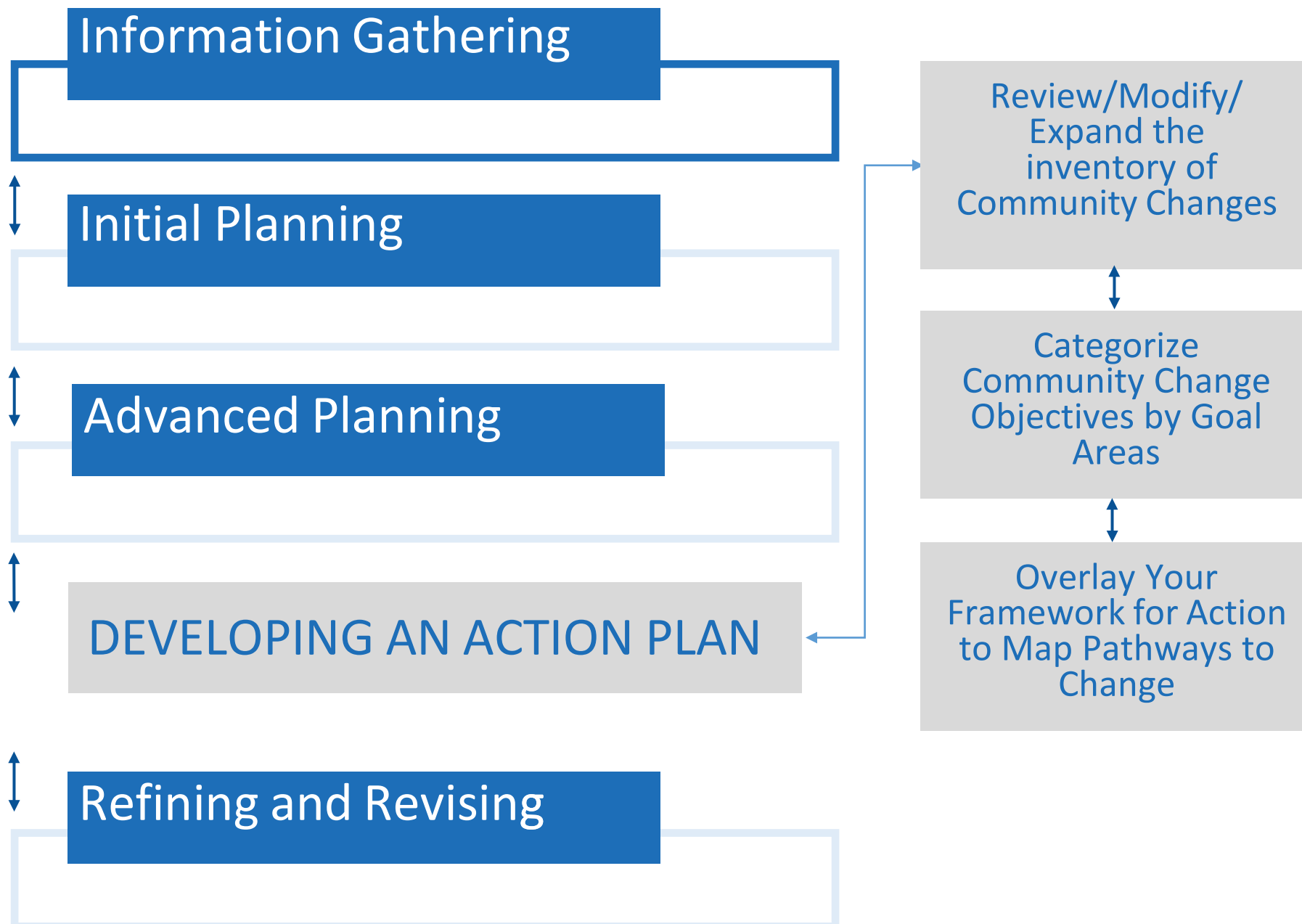

Behavior Strategies

Identify action across behavior strategies to address your problem at multiple points of intervention. Behavior strategies include providing information, enhancing services and supports, changing consequences (e.g., incentives and disincentives), and modifying policies and boarder systems that affect the issue.

For more information on develop strategies, visit The Community Toolbox Chapter 1, Section 9:

https://ctb.ku.edu/en/table-of-contents/overview/model-for-community-change-and-improvement/community-action-guide/main

Review the Inventory of Community Changes

1. For each sector of the community to be involved, refer to the following examples of possible changes that might be sought.

2. Review the following Inventory of Potential Changes that might be sought for each sector. Consider which might be useful in your group's efforts to bridge the Word Gap. Modify these potential changes and delete or add new ones to fit your community's needs, resources, and barriers.

3. Potential changes your group identifies might be directed at behaviors of individual family members, influential people within organizations, or directed towards the environment by altering the programs, policies, and practices of institutions or organizations. Your group should attempt to make changes in a variety of different sectors and levels in your community using a mix of strategies to bring about more change and improvement.

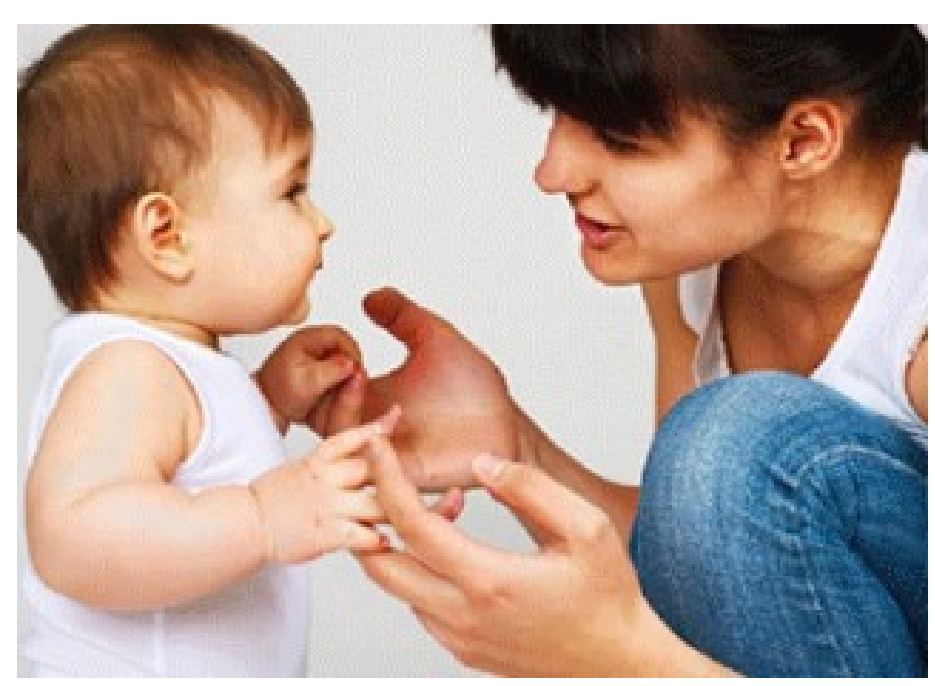

Inventory of Potential Changes by Sector

Please review this list of potential changes by sector. The list notes how particular programs, policies, and practices might be changed to contribute to the mission of bridging the Word Gap. These changes are organized by behavior strategies. Identify 5 to 10 changes that might be the most helpful to the mission of bridging the Word Gap in your community. Modify these possible changes, and delete or add new ones, to fit your community's needs, resources, and experiences. Consider the evidence of effectiveness for some of the strategies, when possible. Examples are provided.

HEALTH CARE

Overview: The Health Care sector includes maternal and child health providers who reach new and expectant parents and their infants and young children. Examples are pediatricians, pediatric nurses, WIC nutritionists, and other health providers (e.g., midwives; obstetric nurses, and clinical nurse specialists). Because nurses, pediatricians, and other health care professionals reach such a large proportion of expectant and new parents, they are in a unique position to convey the message to parents about how language interactions with children can promote brain development and build language and literacy skills.

Providing Information and Enhancing Skills

1. By__________, distribute information to places where families go for pediatric primary health care (e.g., hospitals, clinics, public health settings, and private pediatric settings) to educate health care professionals about the Word Gap and the importance of educating parents about the importance of talking and interacting with their young children.
2. By______________develop and distribute an information sheet and videos for informing patients in public health departments, public clinics, pediatrician offices, parenting classes of the importance of talking to their child.
3. By__________, meet with directors of programs that provide primary pediatric care to recruit them into participation in a language/literacy promotion program for well-child visits
    a. Talk with Me Baby (TWMB) (http://www.talkwithmebaby.org/).
    b. Reach Out and Read (http://www.reachoutandread.org/).
4. By____________, generate a report that documents how training on the language nutrition intervention resulted in changes in nurses' behavior during well-child visits, in parents' talk and interaction with their young babies, and children's expressive communication.
5. By__________, ____

Enhancing Services and Supports

1. By__________, meet with interested clinic teams to determine readiness to participate and implement Talk with Me Baby or other pediatric "language nutrition intervention" and identify potential barriers to implementation.
2. By____________, identify an internal "clinic champion" who will work with nurse providers and support day-to-day implementation of TWMB.
3. By______________educate nurses and health care providers who will conduct well-baby visits in the strategies for promoting language/literacy.
    a. Talk with Me Baby (http://www.talkwithmebaby.org/).
    b. Reach Out and Read (http://www.reachoutandread.org/).
4. By__________, carry out observations to ensure that nurses/health care providers are delivering the selected language/literacy program with fidelity.
5. By__________, provide nurses/health care providers with coaching/feedback on their implementation of language/literacy intervention.
6. By__________, provide opportunities for health care providers to provide feedback on their perceptions of the acceptability/feasibility of the language/literacy intervention during well-child visits.
7. By__________, nurses or other health care providers trained in language/literacy promotion strategies will implement them during well-child visits for children from birth to 3 years.
8. By__________, ____

Modifying Access, Barriers, and Opportunities

1. By__________, identify how nurse providers or other health care practitioners will be able to find time in their schedules to attend training and coaching in language/literacy promotion.
2. By__________, provide ways for nurse provider or other health care professionals to deliver training in ways that are tailored appropriately to address culturally/linguistically diverse families.
3. By__________, provide parents with an app that reinforces the language/literacy promotion strategies that they learn from the health care provider.
   a. Talk with Me Baby App: (http://www.talkwithmebaby.org/download_the_app.)
   b. Háblame Bebé App: (https://apps.apple.com/us/app/h%C3%A1blame-beb%C3%A9/id1349793334.)
   c. Talk Around Town App: (http://talkaroundtown.waypoint-platform.com/)
4. By__________, ____

Changing Consequences (e.g., altering incentives and disincentives)

1. By__________, provide Continuing Education Credits for health care providers who participate in selected language/literacy training.
2. By__________, provide health care providers with apps that they can suggest to parents that reinforce them for talking and interacting with their children.
3. By__________, ____

Modifying Policies and Systems

1. By___________, work with clinics to establish a way to screen children for delays in language.
2. By__________, clinics will document language screening of children in the electronic health record.
3. By__________, work with clinics to establish information that will help them refer children who screen positive for language delays to appropriate sites that can provide more intensive support for children's language.
4. By__________, health care providers engaged in language/literacy will begin to document their implementation of language intervention as part of children's electronic health record.
5. By__________, carry out discussions with state Medicaid officials to make sharing "language nutrition" information a billable service in preventive pediatric care.
6. By__________, ____

CHILD CARE/ EARLY LEARNING

Overview: The Child Care/Early Learning sector is inclusive of those professionals and providers conducting programs of early care/early education for children birth to five years. Examples include Early Head Start, Head Start, Pre-K, community-based child care, family child care, among others (e.g., privately funded preschool). Intervention for reducing the Word Gap and promoting early communication is critical to promotion of children's school readiness from the earliest opportunity.

Providing Information and Enhancing Skills

1. By__________, conduct events communicating information that introduces child care and early learning personnel to the Word Gap (e.g., presentations, professional development activities, workshops, forums).
2. By__________, provide information about Bridging the Word Gap through presentations, written materials, websites, videos, webinars.
   a. The Promise: (https://www.youtube.com/watch?v=F9HWlIQ1nZE.)
   b. Cox Campus—Talk with Me Baby—Early Educators:
      (http://www.talkwithmebaby.org/early_educators).
   c. Educare Talks! (https://vimeo.com/252896066)
   d. Promoting Communication for Language in Kids: (http://www.talk.ku.edu/.)
   e. PC Talk Strategy Videos: (http://www.talk.ku.edu/tools/videos/)
   f. PC Talk Webinar: (https://mediastream.ksde.org/Media/CSAS/supportinglanguagedev040319.mp4)
   g. LENA Grow Professional Development Program: (https://www.lena.org/lena-grow/).
   h. Talk with Me Baby---Meet Baby Ella video: (https://www.youtube.com/watch?v=mGWjkrErxOw).

        i. TMW-The Power of Words video: (https://tmwcenter.uchicago.edu/the-power-of-words/).
3. By__________, use school newsletters and parent meetings to inform parents/guardians about the Word Gap and ways that they can Bridge the Word Gap.
4. By__________, providing staff and teacher in-service trainings to build teacher and caregiver capacity to use strategies to promote communication and language to bridge the Word Gap in child care and early learning program classrooms.
    a. PC TALK Intervention http://www.talk.ku.edu/
    b. PC TALK Videos http://www.talk.ku.edu/tools/videos/
    c. LENA Grow https://www.lena.org/solutions/
5. By__________, provide information for parents and guardians to build capacity to use strategies to promote communication and language.
    a. PC TALK (Promoting Communication): http://www.talk.ku.edu/
    b. Talk Read Play (Brochure): https://www.thefamilyconservancy.org/TalkReadPlay/
    c. Zero-to-Three: https://www.zerotothree.org/early-learning/language-and-communication
6. By__________, provide information about dialogic and shared book reading interventions with staff and families.
    a. Talking is Teaching: https://talkingisteaching.org/resources/literacy
    b. Reading Rockets: https://www.readingrockets.org/audience/professionals/preschool
7. By__________, ____

Enhancing Services and Supports

1. By__________, provide teacher in-service professional development on Bridging the Word Gap Strategies.
2. By__________, choose a language intervention to use in classrooms
    a. PC TALK (Promoting Communication): http://www.talk.ku.edu/
    b. LENA Grow: https://www.lena.org/solutions/
    c. Cox Campus Online Professional Development: https://app.coxcampus.org/?_ga=2.33062974.1683210649.1572397476-52414245.1572397476#!/courses/categories/5bec8f89447831002c7c3b60
3. By__________, provide training to programs on how to share information with parents about Bridging the Word Gap with parents.
4. By__________, provide training for parents and guardians on strategies to Bridge the Word Gap
    a. PC TALK (Promoting Communication) http://www.talk.ku.edu
    b. American Academy of Pediatrics - Toolkit https://www.aap.org/en-us/literacy/Pages/default.aspx
5. By__________, provide cultural competence training for program/school staff.
    a. CCEI course on cultural competence: https://www.cceionline.com/new-course-ccei-covers-cultural-competence-diversity-child-care-environment/
    b. NAEYC: https://www.naeyc.org/our-work/public-policy-advocacy/cultural-competence
6. By__________, select appropriate assessment tools for documenting child outcomes.
7. By__________, provide training to staff on assessments to document child outcomes.
8. By__________, provide training to staff on progress monitoring assessments to document child progress.
    a. Infant-Toddler Individual Growth and Development Indicators: https://igdi.ku.edu/
9. By__________, provide training to staff on surveys or self-checklists to document intervention delivery.
    a. PC Talk: https://talk.ku.edu/tools/self-checks/
10. By__________, encourage staff to build in time to observe interactions between teachers and children and to document level of strategy/intervention use.
11. By__________, partner with community organizations including libraries, bookmobiles, and museums to promote collaboration around early literacy events for children and families.
12. By__________, partner with local and national institutions of higher education around word gap activities including webinars, conferences and expert guest speakers, research and professional development activities.
13. By__________, encourage staff to build in time to observe interactions between teachers and children and to document level of strategy/intervention use.
14. By__________, ____

Modifying Access, Barriers, and Opportunities

1. By__________, identify resources and supports to facilitate a curriculum that includes activities that support early communication and language development to bridge the Word Gap.
2. By__________, provide space for parent trainings and community events.
3. By__________, identify parent and community events and activities that promote early literacy, language development and school readiness.
4. By__________, identify people who can make information and communication documents in languages of the community.
5. By__________, promote opportunities for screening and assessment of children's language.
6. By__________, promote and support access and equity of digital technology as a resource.
7. By__________, examine families' barriers to accessing high quality early care and education programs including transportation, availability of infant-toddler programs, and affordability.
8. By__________, promote parent access to community events addressing the Word Gap by providing child care.
9. By__________, expand accessibility of professional development opportunities to community child care providers and early educators by providing substitute care providers/early educators.
10. By__________, individualize Word Gap interventions for early care and education programs to make it more likely that interventions/strategies will be implemented by child care providers/early educators.
11. By__________, provide time for teachers to meet and reflect on intervention/strategy use and to plan for future use.
12. By__________, provide time and resources for teachers to videotape use of strategies/intervention so that videos can be used for self-reflection and training.
13. By__________, build the capacity of early education staff to present to peers and parents about the importance of addressing the Word Gap and strategies to use.
14. By__________, ____ ___________

Changing Consequences (e.g., altering incentives and disincentives)

1. By__________, establish an incentives program to encourage child care providers to implement Word Gap curriculum.
2. By__________, provide incentives for child care and early learning programs to train parents and guardians on ways to bridge the Word Gap.
3. By__________, provide opportunities for staff to teach peers and parents about the Word Gap so they can align the intervention with their curriculum.
4. By__________, include review of the Word Gap information as a component of parent and teacher meetings and conference.
5. By__________, ____

Modifying Policies and Systems

1. By__________, inform child care and early learning staff about how research addressing the Word Gap and intervention strategies align with their policies and practices (Early Head Start policy, Parents as Teachers, Zero to Three, NAEYC etc.).
2. By__________, integrate into training and licensing procedures for child care/early learning staff about assessments that inform intervention practice.
   a. Individual Growth and Development Indicators website: http://igdi.ku.edu/
   b. PC TALK (Promoting Communication): http://www.talk.ku.edu/
   c. LENA Grow: https://www.lena.org/solutions/
3. By__________, ____

BUSINESS

Overview: The Business sector includes the community's companies, trade groups (Unions), chambers of commerce, and economic activities broadly defined. As employers, the business sector has a vested interest in the future workforce by supporting local edu-cation affected by the Word Gap and promoting family-friendly policies that help parents provide nurturing environments for children. As community partners, businesses seek to promote positive public relations among consumers regarding their activities.

Providing Information and Enhancing Skills

1. By__________, encourage local businesses to distribute information to employees that discusses the Word Gap and

the importance of talking and interacting with young children to ensure a rich language environment at home (e.g., *Talk, Read, Play* Kansas City flyer; https://www.thefamilyconservancy.org/talkreadplay/).
2. By_________, encourage local business to educate employees about the importance of early brain development, and the way parents can nurture their child's development. Provide information briefs describing the word gap that may be of interest to employees who are parents or who may interact with infants and young children. Next Generation: see: http://thenextgeneration.org/files/Word_Health_v3.pdf.
3. By_________, develop and distribute information that discusses that school readiness starts at birth. Post this information and other Word Gap resources and "talking tips" in employee breakrooms. Parent handout about early literacy and language: https://www.zerotothree.org/resources/300-what-we-know-about-early-literacy-and-language-development.
4. By__________, encourage businesses to display Word Gap information in their company's commitments and contributions to improving community resources on the company's website.
5. By__________, disseminate information about the Word Gap and what parents can do about it through local businesses (e.g., grocery stores, banks, print and broadcast media, employees' unions, gas stations, malls, shopping centers, etc.)
6. By__________, ____

Enhancing Services and Supports

1. By__________, encourage businesses to partner with community sectors/organizations that support parenting (home visiting, child care, pediatric health services) and promote activities to address the Word Gap.
2. By__________, investigate company specific resources locally and nationally that support children's language and literacy learning. For example, the National Laundry Cares Foundation and their funding of literacy centers and materials in local laundromats – making learning opportunities available to children in everyday spaces/places (https://laundrycares.org/family-read-play-learn-space-kits/)
3. By_________, encourage businesses to develop family-friendly work policies that support parents of young children to create stable, supportive households (e.g., paid and/or protected time off following the birth of a baby; flexible scheduling options for working parents with young children so that they have the ability to spend time during the workweek caring for and engaging with their children).
4. By__________, encourage businesses to provide onsite company sponsored/supported child care services to the children of employees.
5. By__________, provide professional development for caregivers around using strategies that promote communication.
   a. PC TALK (Promoting Communication) http://www.talk.ku.edu/
   b. Talk Read Play (Brochure) https://www.thefamilyconservancy.org/TalkReadPlay/
6. By__________, allow employees to participate in reading programs with local childcare facilities and schools.
7. By__________, encourage businesses to work with local employee unions to promote Word Gap prevention strategies/services among their members.
8. By__________, ____

Modifying Access, Barriers, and Opportunities

1. By__________, use the local, regional, and national business associations (Chambers of Commerce) to assist in organizing the business community's efforts to bridge the Word Gap.
2. By__________, provide opportunities for expert speakers to address businesses about the Word Gap and strategies that can used to promote children's language acquisition.
3. By__________, encourage businesses to provide employees opportunities to receive training and support/coaching on how to promote children's language acquisition.
   a. Zero to Three: https://www.zerotothree.org/resources/300-what-we-know-about-early-literacy-and-language-development
4. By__________, encourage businesses to provide community service opportunities to organizations and nonprofits that support bridging the Word Gap as a priority.
5. By__________, encourage businesses to make corporate contributions to nonprofit organizations that carry out Bridging the Word Gap interventions through grants and corporate matching of volunteer hours to nonprofit organizations.
6. By__________, encourage businesses to reach out to their company boards and/or the local philanthropic community and lobby for funding that supports and maintains local Word Gap intervention and prevention efforts.

7. By__________, encourage businesses to work with local nonprofits, and libraries to develop the infrastructure necessary to narrow the digital divide as a means of preventing the Word Gap (e.g., make broadband connections more affordable; provide accessible digital literacy classes).
8. By__________, ____

Changing Consequences (e.g., altering incentives and disincentives)

1. By__________, establish incentives that will encourage local businesses to engage in Bridging the Word Gap activities and strategies.
2. By__________, create and publish individual businesses commitments to engage in bridging the Word Gap activities and strategies, and create a system to report their progress regularly.
3. By__________, create and publish reports in the local media to report the contributions of local businesses and the Chamber in bridging the Word Gap activities and strategies.
4. By__________, encourage businesses to advertise stories of employees' (as parents) personal experiences engaging in Bridging the Word Gap with their children.
5. By__________, encourage businesses to raise funding and contribute funding to all local efforts to Bridge the Word Gap.
6. By__________, ____

Modifying Policies and Systems

1. By__________, create policies within the Chamber of Commerce and Trade Unions that support and reward their businesses that engage in bridging the Word Gap and other health promotion efforts.
2. By__________, advocate for policies that support practices that address the Word Gap such as child care programs and early learning programs that build children's readiness for school.
3. By__________, hold politicians and political candidates accountable for supporting universal early learning programs that include mandates to Bridge the Word Gap.
4. By__________, commit corporate giving to support efforts to Bridge the Word Gap.
5. By__________, support an effort to create a local not-for-profit organization to sustain the community-wide effort to Bridge the Word Gap over time.
6. By__________, ____

MEDIA

Overview: The Media sector includes the range of print and electronic messaging tools that include video, TV, websites, blogs radio, news, social media, newspapers, magazines, and books. Media organizations and companies operate to create, publish, broadcast, and stream messages to the public. Because most have goals or requirements to produce messages to the public, they are in an excellent position to message the Word Gap and inform the public. For example, radio and TV broadcast stations are required by law to identify needs in their listening community and address them through local programming.

Providing Information and Enhancing Skills

1. By__________, develop core public awareness campaign messages about the Word Gap that can be adapted to meet the needs of specific audiences in the community.
2. By__________, determine which communication channels will be utilized for the community awareness campaign about the Word Gap (e.g., printed resources, website, social media, e-newsletter, blogs, broadcast media, billboards)
3. By__________, keep the media and public awareness campaigns informed about activities, research, conferences, and public relation efforts addressing the Word Gap.
4. By__________, provide information about current public awareness campaigns about Bridging the Word Gap through presentations, written materials, websites and videos.
    a. Talking is Teaching https://talkingisteaching.org/talking-is-teaching-campaigns
    b. Zero to Three
    c. Strategies for Children http://www.strategiesforchildren.org/EEA.html
    d. Campaign for Grade Level Reading https://gradelevelreading.net/our-work/school-readiness.
5. By__________, use social media or other selected communication channels to inform stakeholders about the Word Gap and strategies to Bridge the Word Gap.

6. By__________, provide trainings to build community-specific public awareness campaigns.
7. By__________, provide training to programs and template materials (e.g., example press release) on how to share information with the community about Bridging the Word Gap.
8. By__________, ____

Enhancing Services and Supports

1. By______, establish partnerships with media companies and organizations to offer pro bono (i.e., volunteer) supports and resources to help spread the word.
2. By__________, provide public service announcements about the availability of supports and services for parents and teachers to decrease the Word Gap.
3. By__________, identify and disseminate information about community events and activities around the Word Gap.
4. By__________, provide public awareness campaigns that outline what community members can do to Bridge the Word Gap.
5. By__________, ____

Modifying Access, Barriers, and Opportunities

1. By__________, identify resources and supports for Public Service Announcements on Bridging the Word Gap.
2. By__________, host community events to disseminate research on the Word Gap, and use social media to highlight the event and provide information to those who were unable to attend.
3. By__________, provide information and communication documents in languages of the community for culturally relevant dissemination.
4. By__________, engage influential community members to disseminate information about Bridging the Word Gap, including parents and other leaders.
5. By__________, identify policy leaders/champions favorable to Word Gap programs and practices and provide opportunities for them to communicate their stories.
6. By__________, ____

Changing Consequences (e.g., altering incentives and disincentives)

1. By__________, provide public incentives through media (e.g., published posting of child care facilities that support Word Gap strategies) to encourage child care and early learning programs to train parents and guardians on how to bridge the Word Gap.
2. By__________, establish an ongoing process to promote public recognition for community efforts Bridging the Word Gap.
3. By__________, ____

Modifying Policies and Systems

1. By__________, promote messages and stories identifying gaps in existing policies that influence programs and practices affecting young children and families related to Bridging the Word Gap.
2. By__________, recognize and promote in the media examples (i.e., best practices) of policies and systems that are effectively bridging the Word Gap to promote changes locally.
3. By__________, disseminate communications that encourage policy leaders to support early learning programs and practices that impact prospective parents and children birth to three.
4. By__________, ____

PHILANTHROPY

Overview: Philanthropy advance solutions to social problems and promote well-being through investment of resources. Within their charters and articles of incorporation are mission statements and goals that target their investments toward solutions to social problems of prioritized interest. Poverty and “school readiness” are often among these goals, and Bridging the Word Gap is largely an untapped opportunity to support programs and services for addressing both problems. Thus, local foundations can play a key role.

Providing Information and Enhancing Skills

1. By__________, develop a resource listing of private foundations and public sources of funding (federal, state, and local) for community-based efforts such as Bridging the Word Gap .
2. By__________, determine and disseminate a strategic plan that includes the allocation of resources for Bridging the Word Gap programs and services.
3. By__________, give Word Gap messaging a priority location in the organization's website and print materials.
4. By__________, support activities (e.g., conferences, symposia) to provide information about and how to address the Word Gap.
5. By__________, ____

Enhancing Services and Supports

1. By__________, provide resources to organizations addressing the Word Gap.
2. By__________, support the development of strategic and action plans by organizations to address the Word Gap.
3. By___________, support research and evaluation that tracks and reports the landscape of community sectors with programs and practices in place that bridge the Word Gap.
4. By__________, ____

Modifying Access, Barriers, and Opportunities

1. By__________, work with funders to develop the Word Gap as a priority for local funding support by providing information and linking the Word Gap to their mission and portfolio of causes.
2. By__________, work to place activists for children or the Word Gap in the community on organization's boards.
3. By__________, provide funding for activities and/or organizations that disseminate Word Gap information and messaging.
4. By__________, ____

Changing Consequences (e.g., altering incentives and disincentives)

1. By__________, provide public credit, recognition, and thanks to local funders supporting Bridging the Word Gap efforts.
2. By__________, provide incentives (e.g., bonuses) to sectors, organizations, programs, and providers that improve outcomes related to Bridging the Word Gap.
3. By_________, establish a means of maintaining financial support for the BWG community partnership over the long-run.
4. By___________, incentivize policies and programs that ensure that all young children are experiencing rich language experiences in various community settings.
5. By__________, ____

Modifying Policies and Systems

1. By__________, establish a priority funding commitment for local Word Gap initiatives within the funding organization.
2. By___________, promote with potential board members, donors, and investors the importance of supporting local Word Gap initiatives.
3. By___________, identify existing policies that hinder Bridging the Word Gap efforts and support change.
4. By__________, ____

RESEARCH AND EVALUATION

Overview: The Research and Evaluation sector includes partners in a position to provide technical support to the community-wide effort to Bridge the Word Gap. Particularly desirable partners are those with the experiences with both community and child-family level interventions, development, and evaluation. The role of researchers and evaluators in a community partnership is to provide the information and technical support for identifying the need and monitoring and reporting progress of the community's effort to Bridge the Word Gap.

Providing Information and Enhancing Skills

1. By__________, gather and disseminate information about the local need for an effort to Bridge the Word Gap.
    a. Resource: https://www.aecf.org/resources/2019-kids-count-data-book/
2. By__________, provide regular reports (e.g., annually) with updated data to provide to community leaders regarding the level of the Word Gap in the local community.
3. By__________, disseminate information about the Word Gap and evidence-based practice.
    A. Review the special issue of *Early Childhood Research Quarterly* on the Word Gap (2019) and other documents, studies, BWG briefs, and resources contained in this document, and online at the BWG Research Network website.
3. By__________, circulate information on your organization's interest in the Word Gap and how to support community efforts to Bridge the Word Gap.
    A. Review Greenwood et al. (2017) "Conceptualizing a public health prevention intervention for bridging the 30 Million Word Gap. *Clinical Child and Family Psychology Review 20* (1), 3-24.
4. By__________, provide informational briefs (e.g., fact sheets) regarding the local Word Gap to help promote the issue.
5. By__________, provide information about the Word Gap based on research.
6. By__________, ____

Enhancing Services and Supports

1. By__________, provide research leadership on the Word Gap and effective intervention practices for reducing the Word Gap.
2. By__________, provide technical assistance and support for documenting activities addressing the Word Gap.
    A. Consider use of the *Community Check Box* Evaluation System (https://checkbox.ctb.ku.edu).
3. By__________, provide technical assistance evaluating community efforts to monitor progress reaching Word Gap prevention goals.
4. By__________, provide support for collecting progress data and reporting and interpreting results concerning achievements to the community leaders/partners as well as where renewal and redirection of efforts are needed.
5. By__________, ____

Modifying Access, Barriers, and Opportunities

1. By__________, create (or join) collective efforts to bridge the Word Gap (e.g., Bridging the Work Gap Research Network [https://bwg.ku.edu/], Thirty Million Words [https://tmwcenter.uchicago.edu/], Too Small to Fail [http://toosmall.org/], etc.)
2. By__________, identify local communities with elevated Word Gap risk (e.g., high poverty, low attendance) who are likely to benefit by addressing the Word Gap and improving young children's school readiness.
3. By__________, identify local leaders and partners to promote Word Gap messaging and support a call to action.
4. By__________, assess current public health and early education initiatives to align causes and leadership around the Word Gap.
5. By__________, create opportunities to seek funding for Word Gap prevention through local, regional, and national granting agencies concerned with school readiness promotion of young children.
6. By__________, use messaging, technology, and community champions to make it easy for community leaders who might want to collaborate to address the Word Gap.
7. By__________, ____

Changing Consequences (e.g., altering incentives and disincentives)

1. By__________, provide professional development opportunities and technical assistance to community coalitions and local efforts seeking to bridge the Word Gap.
2. By__________, examine and communicate the negative consequences of not addressing the underlying issues (e.g., poverty, inequities) fueling the Word Gap to individuals, neighborhoods, cities, and the nation.
3. By__________, ____

Modifying Policies and Systems

1. By__________, advocate for and support community-engaged research scholarship in your institution of higher learning or organization.

2. By__________, work to identify and support any new Early Learning Initiatives including Pre-K initiatives occurring in your community/region, and promote how they can address the Word Gap.
    A. Review the BWG Brief for Policy Makers.
3. By__________, work toward favorable policy in local and federal programs for young children affected by poverty. At the federal level these social programs include:
    A. The Head Start Act of 1981, a community-based program for low-income families with pregnant women, infants, and toddlers up to age 3 (Early Head Start) and ages 3 to 5 (Head Start).
    B. The Title V Maternal and Child Health Services Block Grant Program, a key source of states' support for promoting and improving the health and well-being of the nation's mothers, children, including children with special needs and their families (https://mchb.hrsa.gov/maternal-child-health-initiatives/title-v-maternal-and-child-health-services-block-grant-program).
    C. The Individuals with Disabilities Education Act Part C (infant and toddlers) and Part B (preschoolers) that provides resources or early intervention services for children from birth to 36 months of age with atypical physical, cognitive, communication, adaptive and social or emotional development. (https://sites.ed.gov/idea/statuteregulations/)
    D. The Title I of the ESEA (Elementary and Secondary schools Act 1965), provides additional funds to public schools with high portions of low SES eligible children for extra education programming. (https://www2.ed.gov/programs/titleiparta/index.html
4. By__________, reach out to the local professional organization chapters of child caregivers/home visitors/pediatricians to ensure local policies, programs, practices are using accountability standards to positively address the Word Gap.
5. By__________, ____

FAITH-BASED ORGANIZATIONS

Overview: The Faith-based sector includes the local religious organizations, churches, sects, and social service agencies, charities, and their membership who are in a position to support and champion the effort to bridge the Word Gap through their activities and services. They are an important member of this community partnership.

Providing Information and Enhancing Skills

1. By__________, develop and distribute interesting and easy-to-read fliers on the Word Gap initiative that highlight the importance of talking and engaging young children in the home and in faith-based settings.
    a. Fliers should provide simple and easy-to-implement examples on ways to increase interactions to increase words said in the home and in other places (e.g. LENA talking tips https://info.lena.org/14-talking-tips)
    b. TALK, Read, Play: (https://www.thefamilyconservancy.org/)
2. By__________, provide information to faith-based centers to include on their websites or social media profiles.
    a. Provide information on language milestones.
    b. Provide ideas for increasing words spoken in the home.
    c. Provide information on the Word Gap initiative and statistics.
3. By__________, identify faith-based organizations with social media accounts and promote messaging that can be easily shared related to the Word Gap (e.g., tags, hashtags, coordinated message).
4. By__________, partner with local media sources to advertise events held at faith-based centers and in the community (e.g., book drives, charity events).
5. By__________, distribute information (e.g., brochures) at faith-based activities about the Word Gap.
    a. TALK, Read, Play: http://thefamilyconservancy.org/talkreadplay
5. By__________, develop and distribute informational posters on "language and early learning" to be displayed at faith-based organizations on their bulletin boards and in their bulletins/programs.
    a. Highlight specific examples from: http://www.talk.ku.edu/wp-content/uploads/2018/11/PC-Poster.pdf
    b. TALK, Read, Play: http://thefamilyconservancy.org/talkreadplay
6. By_________, Include information about the Word Gap as a sermon topic.
7. By__________, ____

Enhancing Service and Supports

1. By__________, provide faith-based organizations with information for local language/speech/early learning services

and resources.
2. By__________, train faith workers to lead parenting/caregiving/babysitter classes addressing early language development.
3. By__________, encourage faith-based organizations to establish either in person or online peer support groups (e.g. expectant parents, parents of young children)
4. By__________, provide faith-based child care curriculum and training for faith-based child care providers (see Child Care/Early Learning sector for curriculum resources) to provide rich language environments.
5. By__________, provide faith-based organizations support to establish a family night that includes opportunities for activities such as story time, young child friendly sing-a-longs, games, and opportunities for parents to see healthy modeling of positive communication with their children.
6. By_________, provide opportunities at holidays and celebrations (e.g., baptisms, baby dedications, back-to-school events) to provide information to congregations addressing the Word Gap (e.g., Talk, Read, Play brochure).
    a. TALK, Read, Play: http://thefamilyconservancy.org/talkreadplay
    b. Talking is Teaching Supports for parents to talk, read, and sing: https://talkingisteaching.org/resources
7. By___________, provide branded brochures and associated merchandise (e.g., book bags, shirts, pens, luggage tags) to provide incentives to congregations about the Word Gap.
    a. TALK, Read, Play: http://thefamilyconservancy.org/talkreadplay
    b. Talking is Teaching: https://talkingisteaching.org/resources
8. By__________, ____

Modifying Access, Barriers, and Opportunities

1. By__________, provide a resource contact for faith-based organizations to refer their questions or request support regarding the Word Gap initiative.
2. By__________, assist faith-based organizations in establishing a book borrow/sharing program to increase access to books to increase opportunities for reading at home.
3. By___________, put into place supports for parents/adults who may not attend services regularly to receive information about the Word Gap (e.g., brochures mailed, links to websites).
    a. TALK, Read, Play: http://thefamilyconservancy.org/talkreadplay
    b. Talking is Teaching Supports for parents to talk, read, and sing: https://talkingisteaching.org/resources
4. By___________, provide support for older siblings to positively interact (e.g., read, play, talk) with younger children to promote rich language environments.
5. By__________, ____

Changing Consequences (e.g., altering incentives and disincentives)

1. By__________, incentivize participation by advertising faith-based organization accomplishments towards bridging the Word Gap through social media or other media sources.
2. By__________, highlight family success stories in faith-based organizations' weekly/monthly fliers, boards, social media, or other sources of communication.
3. By__________, spotlight stories of older siblings reading and otherwise verbally engaging younger siblings.
4. By__________, ____

Modifying Policies and Systems

1. By__________, assist in outreach and advocacy efforts directed towards local policymakers to develop/support policies that support the Word Gap initiative.
2. By__________, evaluate current faith-based child care programs for opportunities to enhance curriculum to address the Word Gap.
3. By__________, be proactive in establishing areas in which the BWG message and effort are compatible with the faith's basic doctrine.
4. By__________, ____

HOME AND FAMILIES

Overview: The Home and Families sector includes the local programs and professionals who provide home-based care and counseling to families. These may include home visitors sponsored through health care (e.g., parenting, nutrition) and early prevention/intervention (e.g., Early Head Start; IDEA Infant/Toddler programs) programs.

Providing Information and Enhancing Skills

1. By__________, conduct events to introduce home visiting and early intervention administrators and personnel to the Word Gap (e.g., presentations, professional development activities, workshops, webinars and forums).
    a. Webinar:https://mediastream.ksde.org/Media/CSAS/supportinglanguagedev040319.mp4
2. By__________, provide information about Bridging the Word Gap through presentations, written materials, websites and videos to home visiting personnel.
    a. Talk, Read, Play: https://www.thefamilyconservancy.org/TalkReadPlay/
    b. Talking is Teaching: https://talkingisteaching.org/resources
3. By__________, use newsletters and parent meetings to inform home visiting staff, parents/guardians and community partners about the Word Gap and ways that they can Bridge the Word Gap.
4. By__________, provide home visiting staff with professional development trainings about strategies to promote communication and language to Bridge the Word Gap to assist them in building parent capacity to support children's language learning.
    a. PC TALK Intervention: http://www.talk.ku.edu/
    b. Webinar: https://mediastream.ksde.org/Media/CSAS/supportinglanguagedev040319.mp4
    c. Talking is Teaching: https://talkingisteaching.org/resources
    LENA Home: https://www.lena.org/lena-home/
5. By__________, provide information for parents and guardians to build capacity to use strategies to promote communication and language.
    a. PC TALK (Promoting Communication) http://www.talk.ku.edu/
    b. Talk Read Play (Brochure) https://www.thefamilyconservancy.org/talk-read-play-campaign/ c. Zero-to-Three: https://www.zerotothree.org/early-learning/language-and-communication
    d. LENA Home: https://www.lena.org/lena-home
    e. TMW: https://tmwcenter.uchicago.edu/tmw-programs-2/
6. By__________, provide information about dialogue and shared book reading interventions with home visiting personnel to disseminate to families.
    a. Talking is Teaching: https://talkingisteaching.org/resources/literacy
    b. Reading Rockets: https://www.readingrockets.org/audience/professionals/preschool
7. By__________, ____

Enhancing Services and Supports

1. By__________, provide home visitors with professional development opportunities on Bridging the Word Gap and interventions/strategies that can be shared with parents.
    a. PC TALK (Promoting Communication) http://www.talk.ku.edu/
    b. LENA Grow https://www.lena.org/solutions/
    c. Zero-to-Three: https://www.zerotothree.org/early-learning/language-and-communication
    d. Literacy Network: https://mediastream.ksde.org/Media/CSAS/supportinglanguagedev040319.mp4
2. By__________, provide training to home visiting programs on how to share information with parents about Bridging the Word Gap.
3. By__________, provide training for parents and guardians on strategies to Bridge the Word Gap.
    a. PC TALK (Promoting Communication) http://www.talk.ku.edu/
    b. American Academy of Pediatrics - Toolkit https://www.aap.org/en-us/literacy/Pages/default.aspx
4. By__________, select appropriate observation and assessment tools for documenting fidelity of intervention delivery by home visiting staff.
    a. http://www.talk.ku.edu/
    b. https://www.lena.org/lena-grow/
5. By__________, provide training to staff on progress monitoring assessments to document child

progress.

a. Individual Growth and Development Indicators for Infants and Toddlers: https://igdi.ku.edu/

6. By__________, provide training to home visiting staff and personnel on surveys or self-checklists to document intervention delivery of Word Gap efforts.
    a. http://www.talk.ku.edu/
7. By__________,encourage staff to build in time to observe interactions between parents and children and to document level of strategy/intervention use.
    a. PC TALK: http://www.talk.ku.edu/
    b. LENA: https://www.lena.org/lena-grow/
8. By__________, partner with community organizations including libraries, bookmobiles, and museums to promote collaboration around early literacy events for children and families.
9. By__________, partner with local community and national home visiting programs and institutions of higher education around Word Gap activities including webinars, conferences and expert guest speakers, research and professional development activities.
    a. Zero-to-Three: https://www.zerotothree.org/early-learning/language-and-communication
    b. Literacy Network: https://mediastream.ksde.org/Media/CSAS/supportinglanguagedev040319.mp4
10. By__________, individualize interventions addressing the Word Gap to make it more likely that interventions/strategies will be implemented by parent(s).

11. By__________, provide time for home visiting staff to meet regularly and reflect on intervention/strategy use by families on their caseload and to share data and discuss successes and challenges with delivering the intervention.
12. By__________, ____

Modifying Access, Barriers, and Opportunities

1. By__________, identify resources and supports to enhance understanding of providers and parents about Bridging the Word Gap.
2. By__________, provide space for professional development activities as well as parent trainings and community events.
3. By__________, disseminate information to providers and parents about community events and activities around the Word Gap.
4. By__________, identify staff who can develop and/or translate information and communication documents in languages of the local community so that home visitors and parents can access materials in their home language.
5. By__________, promote opportunities for providing information about documenting the degree to which the intervention is delivered by multiple sources (e.g., home visitors, parents).
6. By__________, promote parent access to community events by providing child care during events addressing the Word Gap.
7. By__________, provide paid time to allow home visiting staff access to attend professional development activities.
8. By__________, build the capacity of home visiting staff to present to peers and parents about the importance of addressing the Word Gap, and strategies for implementation.
9. By__________, provide opportunities for home visiting staff to align the intervention with their home visiting curriculum.
10. By__________, ____ ___________

Changing Consequences (e.g., altering incentives and disincentives)

1. By__________, establish an incentives program to encourage home visitors to implement interventions around the Word Gap.
2. By__________, provide incentives for home visitors to train parents and guardians on ways to Bridge the Word Gap.
3. By__________, provide opportunities for staff/ parents to have opportunities for discussion of successes and challenges with implementing interventions/strategies addressing the Word Gap.
4. By__________, document and share progress regarding efforts to address the Word Gap.

Modifying Policies and Systems

1. By__________, inform home visiting administrators and/or staff about how research addressing the Word Gap and intervention strategies align with their policies and practices (e.g., Early Head Start policy, Parents as Teachers, Zero to Three, NAEYC .)

2. By__________, inform home visiting staff about assessments and/or observation measures that inform intervention practice addressing the Word Gap.
    a. Individual Growth and Development Indicators: https://igdi.ku.edu/.
    b. PC TALK (Promoting Communication) http://www.talk.ku.edu/
    c. LENA Grow https://www.lena.org/solutions/
3. By__________, inform home visiting staff about assessments and/or observation measures that inform intervention practice addressing the Word Gap.
    a. Individual Growth and Development Indicators: https://igdi.ku.edu/.
    b. PC TALK (Promoting Communication) http://www.talk.ku.edu/
    c. LENA Grow https://www.lena.org/solutions/
4. By__________, ____

GOVERNMENT

Overview: The Government sector refers broadly to the local elected leaders/officials providing responsible leadership to the community, city, and state. These may include mayors, governors, legislators, county officials, and civic employees. Because of their key role in policy formation, implementation, and funding, they are key stakeholders.

Providing Information and Enhancing Skills

1. By_________, access resources about advocacy on behalf of infants, toddlers and young children provided by agencies.
    a. Zero to Three: https://www.zerotothree.org/resources/series/policymakers-advocates
    b. Child Care Aware of America: https://usa.childcareaware.org/advocacy-public-policy/
2. By_________, distribute information on the importance of the early years (birth to 5) to children's brain and language development.
3. By_________, provide information on the adverse effects of the Word Gap to brain and language development, as well as readiness for kindergarten, and later life social, educational, health, and economic outcomes.
4. By_________, provide information on the long-term social and economic impact of the Word Gap and the return on investment of investing in the early years.
5. By_________, provide facts and information on poverty in this geopolitical unit (community, city, state, etc.) by areas likely to be affected (e.g., poverty rate, school readiness, graduation rate, crime rate).
6. By_________, use the "bully pulpit" to engage the media and provide constant messaging on the Word Gap and efforts to bridge the Gap.
7. By__________, ____

Enhancing Services and Supports

1. By_________, create and/or support early learning initiatives through multiple pathways (e.g., creating or supporting a vision, advocating for and identifying exemplary programs, convening stakeholders in planning).
2. By_________, conduct landscape studies of early learning programs, identify local early leaning program deserts, disseminate findings, and make the case for needed improvements, and investments.
3. By_________, bring together multi-sectoral stakeholders and support creation of a coalition/partnership with a mission to bridge the Word Gap.
4. By_________, lead creation of a non-for-profit foundation with the mission to bridge the Word Gap through information, evidence-based practice, and the need to sustain the effort.
5. By_________, make early learning and early childhood education a priority initiative of the administration.
6. By__________, ____

Modifying Access, Barriers, and Opportunities

1. By_________, make it possible for all families to afford access to high quality, early learning programs.
    a. Encourage states to increase child care subsidies.
    b. Encourage federal and state investments in programs to increase number of high-quality early care and education programs such as Early Head Start and Head Start.
2. Provide incentives to quality early education programs to raise their income eligibility levels for families who wish to enroll young children.

3. By_________, convene stakeholders (sectors) around the Word Gap and structure the opportunity for them to make commitments to actions.
4. By_________, support creation of new and expansion of existing early learning programs with an emphasis of promoting language learning.

5. By_________, in collaboration with stakeholders create/encourage collaboration between and with parents that supports Bridging the Word Gap at home.
6. By_________, identify other related initiatives to align the Word Gap as part of these efforts to improve impact and leverage current resources.
7. By__________, ____

Changing Consequences (e.g., altering incentives and disincentives)

1. By_________, create a monthly program honoring persons and organizations that have taken steps to Bridge the Word Gap.
2. By_________, publish and post a running record of efforts being made by persons or organizations to Bridge the Word Gap on a website, social media, or other medium with wide distribution.
3. By__________,provide financial incentives to providers and service provider (e.g., home visitor) programs that contribute to improved outcomes related to addressing the Word Gap.
4. By__________, ____

Modifying Policies and Systems

1. By_________, review existing policies regulating children's care and learning programs and determine how they might support efforts to Bridge the Word Gap.
2. By_________, rewrite policies to promote greater support and taxpayer investment in programs for young children.
3. By_________, partner with organizations and programs serving young children to encourage accountability for measurable improvements in the evidence-based practices they use and support.
4. By__________, promote organizational practices before implementing a new policy that asks, "Is it good for the children?"
5. By__________, ____

LIBRARIES/MUSEUMS

Overview: Local institutions that provide services to support the continuing educational and informational needs of students, teachers, and all community members in welcoming spaces and interesting programs and resources. The institutions in this sector provide additional educational experiences complementary to the local schools and align as excellent community partners.

Providing Information and Enhancing Skills

1. By__________, promote the "Babies Need Words Every Day: Talk Read, Sing, Play: the set of resources available from the Association for Library Services to Children (ALSC) for providing parents with ways to build their children's language and literacy skills: http://www.ala.org/alsc/babiesneedwords.
2. By__________, identify and coordinate a group of community partners to share and help disseminate the "Babies Need Words" materials.
3. By_____________, develop or distribute already-prepared brochures in multiple languages reflecting the local population about the Word Gap. Brochures or fliers should be written in a clear, simple format that highlights the importance of talking and engaging young children with books in the library and at home.
   a. Fliers should provide simple and easy-to-implement examples on ways to increase interactions to increase words said in the home and in other places (e.g. LENA talking tips https://info.lena.org/14-talking-tips)
   b. TALK, Read, Play: http://thefamilyconservancy.org/talkreadplay
   c. Every Child Ready To Read @ your library http://everychildreadytoread.org/
   d. Department of Education Reading Resources: https://www2.ed.gov/parents/read/resources/edpicks.jhtml
4. By_____________, provide informational graphics to include on library or museum websites or social media posts, or to display in public settings.

    a. Provide information on language milestones.
    b. Promote ideas for increasing words spoken in the home.
    c. Provide information on the Word Gap initiative and statistics: https://www.vroom.org/tools-and-resources

5. By_____________, develop and distribute informational posters on "language and early learning" to be displayed on bulletin boards.
    a. Highlight specific examples from: http://www.talk.ku.edu/wp-content/uploads/2018/11/PC-Poster.pdf
    b. TALK, Read, Play: http://thefamilyconservancy.org/talkreadplay
    c. Vroom Resources: https://www.vroom.org/tools-and-resources
6. By____________, create informational interactive displays on language and early brain development. Include books for parents to check out.
7. By_____________, develop a regular story time/play time for parents to come with their infants and toddlers to learn how to engage their children with books to promote early language.
8. By__________, ____

Enhancing Services and Supports

1. By___________, use the Association for Library Services (ASLC) Press Release and Letter to the Editor templates to disseminate information about the Babies Need Words materials available for community agencies to post and distribute.
2. By___________, make librarians aware of the Babies Need Words Talking Points for Librarians and a book list for parents available from Association for Library Services (ASLC) that recommend books parents can use to promote children's language and cognitive growth.
3. By__________, provide training to library or museum staff on how to share information with parents about the importance of talking and reading with children.
4. By__________, provide parents with information for local language/speech/early learning services and resources.
5. By__________, train library and museum staff to address early language development in library programs.
6. By__________, encourage libraries to establish peer support groups (e.g., expectant parents, parents of young children, toddler play time).
7. By__________, provide early language and literacy development curriculum and training for library and museum staff.
    a. Every Child Ready to Read: http://everychildreadytoread.org
8. By__________, ____

Modifying Access, Barriers, and Opportunities

1. By__________, host literacy events at libraries, museums, or in the community and give out books along with brochures and other information about the Word Gap in several languages.
    a. Talk, Read, Play: http://thefamilyconservancy.org/talkreadplay
    b. Dept of Ed Reading Resources: https://www2.ed.gov/parents/read/resources/edpicks.jhtml
    c. Vroom: https://www.vroom.org/tools-and-resources
2. By__________, provide a resource contact for libraries/museums to refer their questions or request support regarding the Word Gap initiative. (See story in School Library Journal about Chicago Children's Museum exhibit on the Word Gap: https://www.slj.com/?detailStory=closing-the-30-million-word-gap-up-front.)
3. By__________libraries and/or museums provide outreach to share the importance of early literacy and enriching language experiences and/or provide programming (such as story time)to early childhood educators, such as: licensed home child care providers, child care center staff, Head Start teachers, preschool teachers, and other child care professionals .
    a. ECRR Toolkit: http://everychildreadytoread.org/shop/
4. By__________, increase access to museums by offering free times for parents.
5. By__________, increase access to libraries and museums for parents by offering extended hours.
6. By_____________, ensure educational materials are available in several languages.
7. By__________, ____

Changing Consequences (e.g., altering incentives and disincentives)

1. By__________, highlight family success stories in library/museum weekly/monthly fliers, boards, social media, or other sources of mass communication.
2. By__________, provide branded brochures and associated merchandise (e.g., book bags, shirts, pens, luggage tags)

to provide incentives to patrons about the Word Gap.

a. Talk, Read Play: http://thefamilyconservancy.org/talkreadplay

b. Talk is Teaching: https://talkingisteaching.org/resourcess

3. By__________, offer meals, child care, free books, discounted rates or other incentives for families to participate in programs about talking and reading to children.

4. By__________, develop partnerships through local early childhood programs (e.g., Department of Health Services) to offer parents and other early educators and professionals credit for taking parenting and/or other professional development classes sponsored through libraries and/or museums. Credits may in turn be used for parenting supplies, resources for professionals and/or child supplies (e.g., diapers, clothing, toys).

a. Descriptions of the national Stork's Nest Program:

https://www.joe.org/joe/1998december/a5.php

http://zphib1920.org/storks-nest/

5. By__________, ____

Modifying Policies and Systems

1. By__________, assist in outreach and advocacy efforts directed towards local policymakers to develop/support policies that support the Word Gap initiative.
2. By__________, emphasize or create a mission statement regarding the Word Gap for the library/museum board and any supporting foundation boards.
3. By__________, advocate for the importance of the Word Gap initiative with city/county councils.
4. By__________, ____

SCHOOLS

Overview: The sector includes the public and private education programs in the community (Pre-K – 12), as well as higher education. The School sector is responsible for the education of the community's children and youth. As such, they have a vested interest in the "school readiness" of each child entering school and ready to learn. Because Bridging the Word Gap experiences of children prior to school entry promote school readiness, the School sector has a stake in facilitating experiences and educating staff and older students of the importance of rich language experiences in literacy and school success.

Providing Information and Enhancing Skills

1. By__________, develop a Bridging the Word Gap (BWG) information toolkit containing multiple messages and formats (paper, electronic, social media; Word Gap websites) regarding what teachers, parents, and students should know about the Word Gap.
2. By__________, disseminate information from the Bridging the Word Gap (BWG) toolkit that school staff and patrons need to know and understand.
3. By__________, ensure that current, timely information on educational attainment is publicly available regarding local poverty and free lunch rates, children's readiness for school, academic achievement, school level discipline rates, suspension, drop-out and graduation rates.
4. By__________, provide timely and frequent information on what the school (district, education area coop) is doing to prevent the Word Gap, including formal and informal efforts.
5. By__________, ____

Enhancing Services and Supports

1. By__________, based on student age and grade level, provide teachers with professional development on ways they can teach high school students about the significant role parents play in fostering their children's early development and language/communication that supports school success and lifetime outcomes.
2. By__________, reach out to the community to engage parent volunteers and parent groups (e.g., Parent Teacher Association) to get out the word about the Word Gap and what can be done.
3. By__________, for older students (high school/higher education), ensure the Word Gap and its message about the importance of talking/interacting more with young children are covered as part of the curriculum related to parenting, home, and community life.
4. By__________, ____

Modifying Access, Barriers, and Opportunities

1. By__________, ensure that classroom teachers are knowledgeable and skilled in using effective instructional and interaction strategies that scaffold interactions for Dual Language Learners (DLL) and students with language/communication delays/disabilities (e.g., Individualized Education Plan [IEP]).
2. By__________, ensure that teachers are trained in using strategies that provide a rich language learning environment for students, including frequent opportunities to communicate with peers using for example, class-wide peer tutoring and peer-assisted learning strategies.
3. By__________, ensure that parent support groups (e.g., PTA) are reaching out to the parents of all children regarding the Word Gap risk and opportunities for parent training and engagement in the Word Gap intervention prevention.
4. By__________, join a larger community partnership in the effort to prevent the adverse effects of the Word Gap.
5. By__________, ____

Changing Consequences (e.g., altering incentives and disincentives)

1. By__________, ensure that school administrators, specialists, and coaches are knowledgeable of the Word Gap, and actions that can be supported to prevent it, including providing teachers with supportive feedback regarding their prevention efforts.
2. By__________, develop incentives that can be used to recognize ways in which local community sectors/organizations (e.g., child care, parent groups, businesses, philanthropic institutions, libraries/museums, etc.) are making significant contributions to Word Gap prevention and promoting school readiness.
3. By__________, highlight success stories by actors including teachers, librarians, administrator, etc who are supporting strategies to address the Word Gap.
4. By__________, ____

Modifying Policies and Systems

1. By__________, include the Word Gap and its prevention strategies as part of local school policies including curriculum standards for students, and professional development/evaluation experiences of school staff.
2. By__________, make Word Gap prevention an expectations standard component of school policies.
3. By___________, ensure policies are in place requiring tracking and reporting annual school success indicators including school readiness, achievement, discipline problems, suspension, dropout, and graduation so that trends and change due to Word Gap prevention actions can be evaluated.
4. By__________, ensure school accountability policies include evaluation of student readiness for school rates that are based on good and frequently used measurement practices.
5. By__________, ____

“If you always do what you’ve always done, you’ll always get what you’ve always got.”

– Henry Ford

CHAPTER 6: REFINING YOUR ACTION PLAN

Building Consensus on Proposed Changes

The purpose of this chapter is to help guide final changes to be sought by your community's initiative for Bridging the Word Gap.

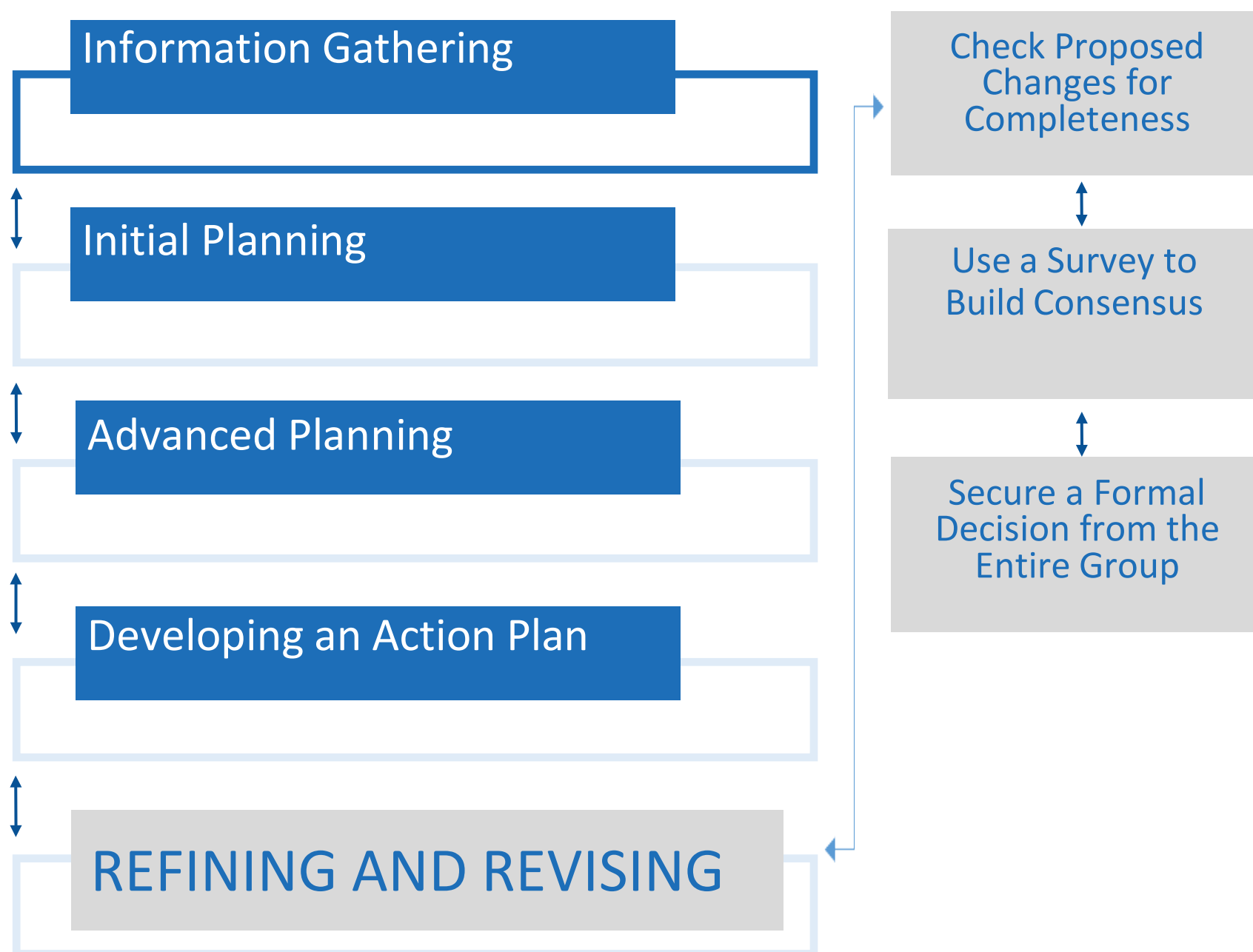

Checking the Proposed Changes for Completeness

The group should review proposed changes within and across each sector, as for completeness. To review the proposed changes in each community sector, we recommend asking two questions:

- Collectively, do these proposed changes maximize this sector's contribution to the mission of promoting children's optimal language development and later school readiness by encouraging caring adults to interact in more frequent high quality interactions with young children?
- What other changes in programs, policies, or practices could or should be made in this sector?

To review the set of proposed changes and their completeness, we suggest asking:

- Would all the changes be sufficient to increase adult language input to desired levels?
- What other changes in programs, policies, or practices could or should be made in the community?
- For each change ask, "Is it good for the children?"

Using a survey (or a more Informal review)

To build consensus to help attract and assure commitments, it is important to build consensus on the changes to be sought. The group may use a survey to review the proposed changes. It can also be done less formally, such as individually or in small groups. We recommend listing all the proposed changes, organized by community sector, along with questions about their importance and feasibility for addressing the mission of promoting health for all.

For each change to be sought, we recommend asking:

- Is this proposed change important to the mission of providing a rich language environment for all young children?
- Is the proposed change feasible?

The following is an example of a useful survey for building consensus on your action plan:

Proposed Changes in the Sector	How important is it to...					How feasible is it to...				
	Not at all				Very	Not at all				Very
Providing nurses/healthcare providers with coaching/feedback on the implementation of a language intervention	1	2	3	4	5	1	2	3	4	5
Providing Continuing Education credits for healthcare providers who participate in language intervention	1	2	3	4	5	1	2	3	4	5

Distribute surveys to all key audiences (e.g., community members, school staff, representatives of funding sources, and experts in child development, etc.) Collect the completed surveys and compute an average rating for the importance and feasibility of each proposed change. Results of this survey can be used to guide final choices and prioritization of community changes to be sought. Proposed changes with high importance and high feasibility ratings should be given higher priority for action; those with lower importance or feasibility, a lower priority. It may be helpful to set a cutoff for choosing priorities. For example, perhaps only those proposed changes with an average rating of 4 or higher on importance, and 3 or higher on feasibility might be included on the final action plan.

Securing a Formal decision from the Entire Group

Seek formal approval of the proposed changes by the members of the initiative. A one-half day action planning retreat or working session can be used to do this effectively.

Seek consensus. Use a formal vote to resolve disputes about changes only when necessary. When the action plan is complete, arrange for all members to vote on the completed plan.

"We cannot solve our problems with the same thinking we used when we created them."

– Albert Einstein

CHAPTER 7: FINALIZING YOUR PLAN

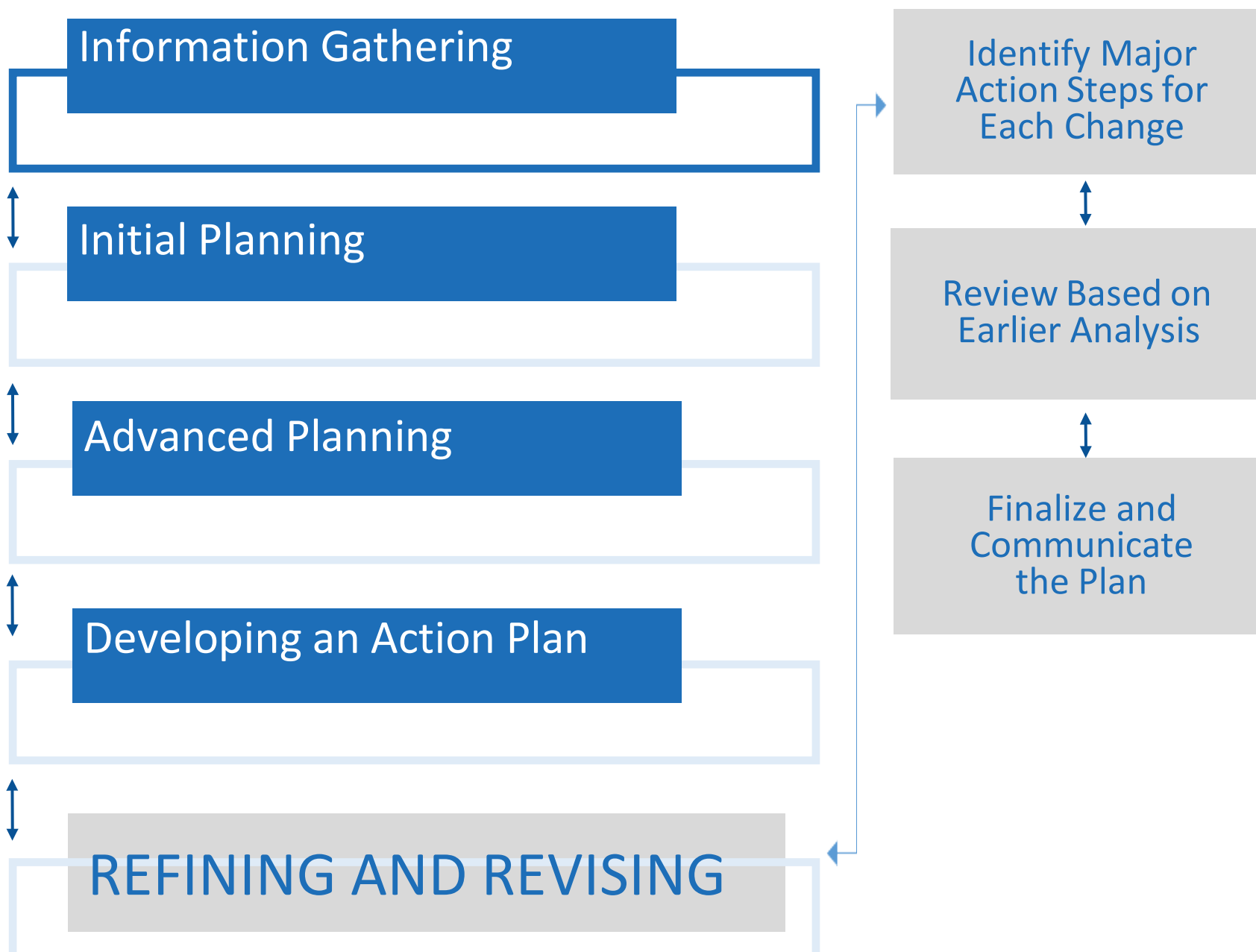

Listing Action Steps for Proposed Changes

The purpose of this chapter is to help prepare action steps for each community or systems change sought by your group. We recommend prioritizing the major action steps needed to attain each proposed change. It is not necessary to list all the action steps--list only the more critical steps required to create the desired change in program, policy, or practice.

Identify Major Action Steps

The action steps detail what will occur, in what amount, by whom, and by when. To prepare action steps for your action plan, define the following for each proposed change (see Example List, pg. #37):

- What actions will be taken (what)
- The responsible agents (by whom)
- The timing (by when)
- Resources and support needed and available (how)
- Potential barriers and resistance
- With whom communication about the plan should occur

Review Based on Earlier Analysis

Use the information gathered in the previous chapters to guide your initiative's action steps for bringing about identified community and systems changes. For example, what does your analysis of assets and resources suggest about responsible agents (by whom)? How can your understanding of potential resources and barriers be used to plan action steps and outline a communications plan?

Finalize and Communicate the Plan

A comprehensive action plan--proposed changes and related action steps--helps communicate to important audiences that the group is clearly organized. It helps demonstrate that the group understands what is needed to be effective in bringing about change. The complete action plan includes action steps for each change to be sought. Organize the changes by community sector, listing each proposed change, and related action steps, in the order in which they are expected to occur. The example that follows illustrates how to list action steps for a specific change to be sought in the Health Care sector. A blank form can be found at the end of this guide, which can be used and adapted (Appendix A, pg. 69).

Action Steps for Identified Changes

(An Example)

Use this page to outline action steps for each identified change to be sought in each community sector.

Community Sector: HEALTH CARE

Community Change to be sought: CLINICS WILL REFER CHILDREN SCREENED POSITIVE FOR LANGUAGE DELAYS TO LOCAL SITES WHO CAN PROVIDE MORE INTENSIVE SUPPORT FOR CHILDREN'S LANGUAGE LEARNING

ACTIONS	BY WHOM	BY WHEN	RESOURCES & SUPPORT NEEDED/AVAILABLE	POTENTIAL BARRIERS OR RESISTANCE	COMMUNICATION
What needs to be done?	Who will take action?	By what date will the action be done?	What financial, human, political and other resources are needed? What resources are available?	What are the policies or practices that are currently in place that could be barriers?	What individuals and organizations should be informed about these actions?
DISTRBUTE INFORMATION AND GET THE WORD GAP MESSAGE OUT	SECTOR LEADERSHIP	JANUARY 2020	NEED FINANCING AND PERSONNEL TO PREPARE; NEED PERSON TO DOCUMENT DISTRIBUTION; ALL ARE AVAILABLE	LACK OF AWARENESS AND TOOLS FOR EFFECTIVE ACTION	PRIMARY HEALTH CARE PROVIDERS AND ALL OTHER SECTOR MEMBERS IN OUR COMMUNITY
MEET WITH PRIMARY CARE PROVIDERS, RECRUIT, & ENROLL THEIR PARTICIPATION	SECTOR VOLUNTEERS AND CHAMPION GUESTS	FEBRUARY 2020	PERSONNEL TO MEET PROVIDERS, PERSON TO DOCUMENT MEETINGS CONDUCTED, COST OF TRAVEL TO LOCATIONS	CURRENT PRACTICE IS NOT TO REFER FOR GREATER LANGUAGE SUPPORT	ALL SECTORS/ MEMBERS IN OUR ACTION PLAN SO THEY CAN COORDINATE AS NEEDED
PROVIDE TRAINING ON SCREENING PROTOCOL AND SCREENING MEASURE	PROVIDERS WHO HAVE VOLUNTEERED TO SCREEN	FEBRUARY 2020	LOCAL RESEARCHERS WHO ARE CERTFIED TRAINERS IN SCREENING PROTOCOL	PROVIDERS MAY NOT GET REIMBURSED FOR THEIR TIME DOING SCREENING	LOCAL PROGRAMS THAT WOULD SERVE AS REFERRAL SITES FOR LANGUAGE INTERVENTION
PROVIDE EDUCATION CREDITS AND ONLINE APPS FOR PARTICIPANTS	AUTHORIZE CREDITS, ACQUIRE APPS AND DISSEMINATION	MARCH 220 AND MAINTAIN THROUGH REST OF YEAR	NEED DOLLARS REQUIRED TO PAY FOR CREDITS AND ONLINE APPS; NEED TO ACQUIRE	EDUCATIONAL CREDIT PROVIDERS POLICIES MAY PREVENT OR RESIS	SECTORS THAT PRVIDE PHILANTHROPY, AND RESEARCH/ EVALUATION

CHAPTER 8: DOCUMENTING PROGRESS AND PROMOTING RENEWAL

Evaluating Efforts

The purpose of this Chapter is to describe how this community coalition may document and evaluate progress implementing the Action Plan designed to ensure all children have the language-learning opportunities they need to stay on track in learning language and literacy, and readiness for school. The Action Plan supports the community's full support behind creation of the strong early language environments that children need, and the resulting beneficial outcomes envisioned.

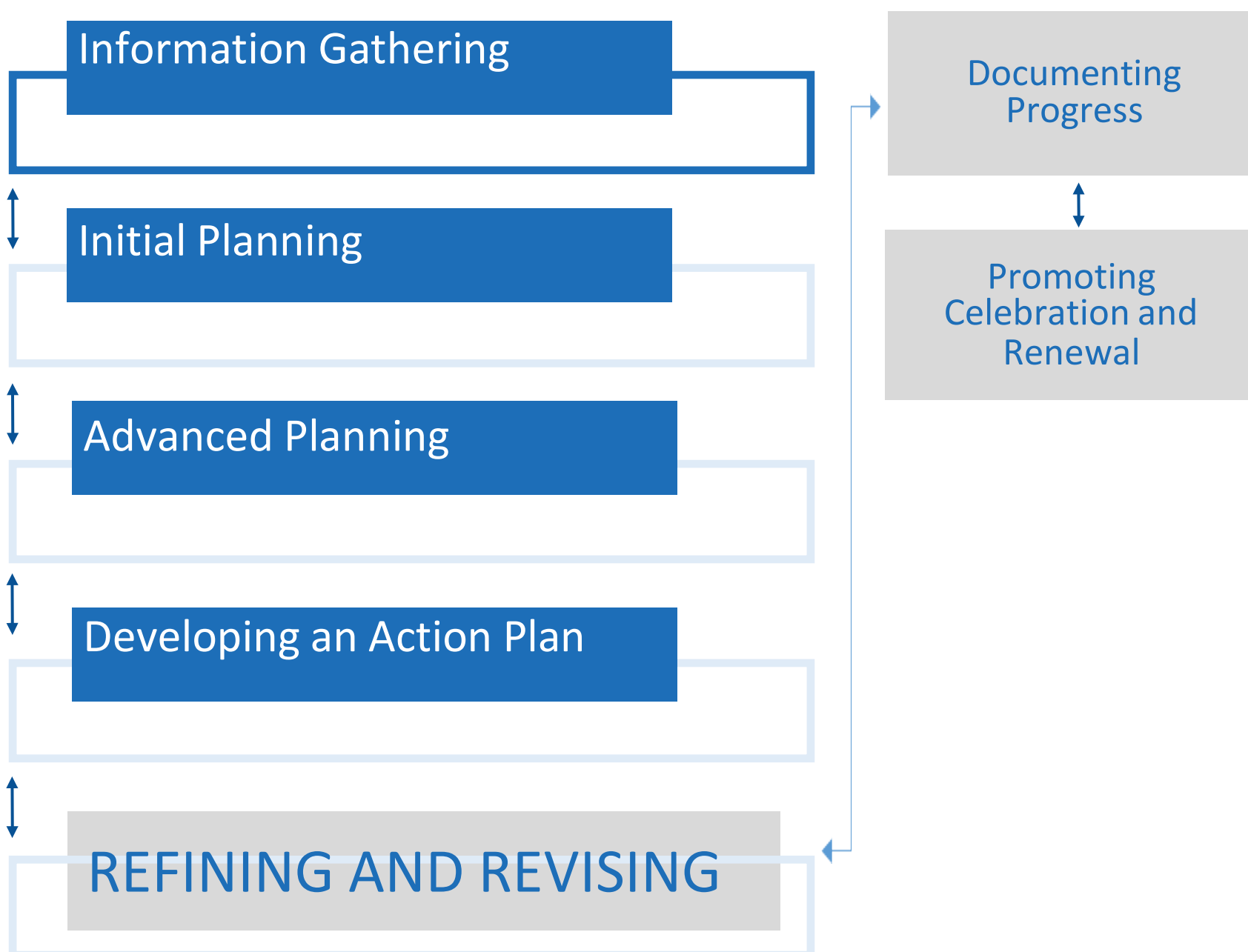

Supportive community change is the goal behind the Action Plan. **We define community change in terms of new or desired modifications to existing policies, programs, and practices that are related to the coalition's mission of bridging the Word Gap**.

Examples of community changes in the Pediatric Health Care Sector include:

- Changing existing medical policies so that language and literacy screening will become a part of children's electronic health records.
- Establishing the means of screening all young children for language delays and risk of language delay in all pediatric health care clinics in the community.
- Establishing a means of referral in pediatric clinics for children with positive screens for risk of or with language delay to other providers (child care, home visiting, faith-based) who can arrange more intensive supports for children's language learning.

Examples of community changes in the Child Care Sector include:

- Identifying and creating an online information bank of evidence-based language promotion interventions and strategies to be used in Child Care practice.
- Developing a set of resources supporting professional development experiences for all providers concerning language promotion strategies that to be used with all children.
- Establishing a means of quarterly universal screening of all children's progress learning language
- Improving the frequency and timeliness of agencies (e.g., Early Head Start, Part C – Infant/Toddler Services) and professions (e.g., speech and language therapists, developmental specialists, pediatric health providers, etc.) required to collaborate in provision of services to individual children around evidence-based, language-promotion practices.
- Increasing implementation of a universal use of an intervention decision-making model based on individual children's response to language intervention and provision of more intensive services to children not making progress.

Community coalitions may fail to achieve their goals because information on their actions, achievements and outcomes are not available, leading to frustration over what is being done and an unclear record of accomplishment. Conversely, documenting progress in implementing the Action Plan assures that planned steps are implemented; and where they are not, adjustments made to identify, address, and overcome barriers moving forward.

Figure 1

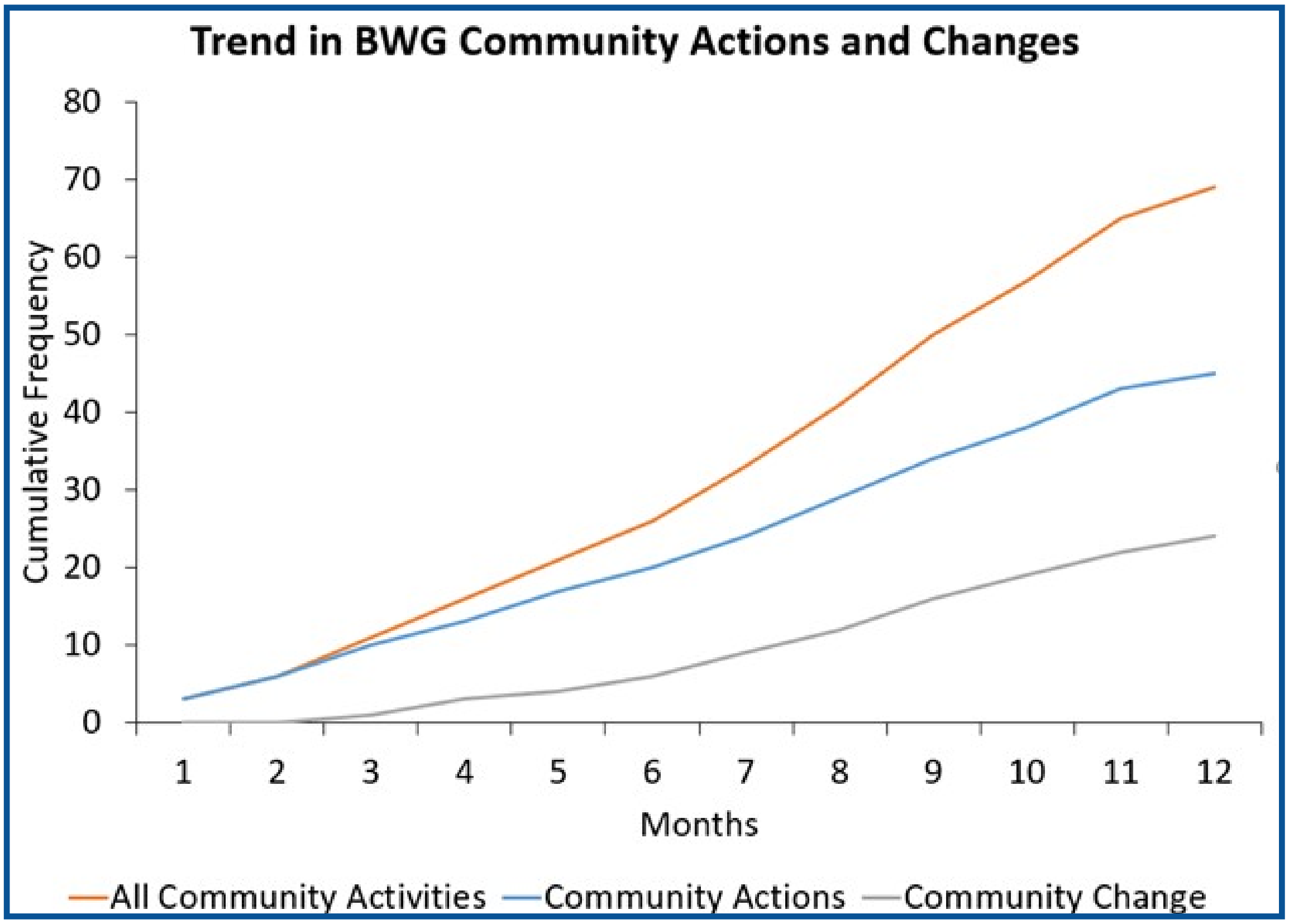

Documenting Progress

The Community Check Box (CCB) Evaluation System was developed for just this purpose. This coalition may use the CCB to document its progress implementing the Action Plan (see the Community Tool Box, https://ctb.ku.edu/en/community-check-box-evaluation-system for more information). The goal of CCB is to "turn data into action" because we "measure what we treasure."

Simply put, the action steps completed by the coalition will be entered into the CCB. Based on systematic documentation of the collective efforts to address the issue based on the action plan, reports will be regularly generated (at least monthly is recommended). Thus, the findings can be reviewed monthly by the coalition, discussed, and celebrated. In cases where there are lulls in progress, then, it can be recognized as occasions of renewal of the group's efforts by identifying challenges and barriers, adjusting activities going forward in the allocation of resources, or using other tools (levers) designed to readjust and put things back on track.

What will be recorded?

The CCB enables the coalition to document each individual accomplishment it makes by date of occurrence and other important features. This information is entered into the online CCB by a trained observer. For example, each accomplishment a text description is provided, which provides a record and also the activity is categorized to better understand key dimensions (e.g., actors, sectors involved).

How will this community project benefit from documenting the efforts of the coalition members?

One important look at the progress being made is to better understand the growth and if the desired community changes are being implemented as shown in Figure 1. In Figure 1, we can see across 12 months the growth in the total number of accomplishment made (orange line). We also see growth in the number of Community Actions (blue line) that started earlier in the project, beginning at the first month, continued thereafter at a higher rate over time because of the increasing number of accomplishments of the coalition. We also see how these actions were linked to growth in the desired Community Changes in policies, programs, and practices (grey line), that starts two months later and grows more slowly.

Figure 2

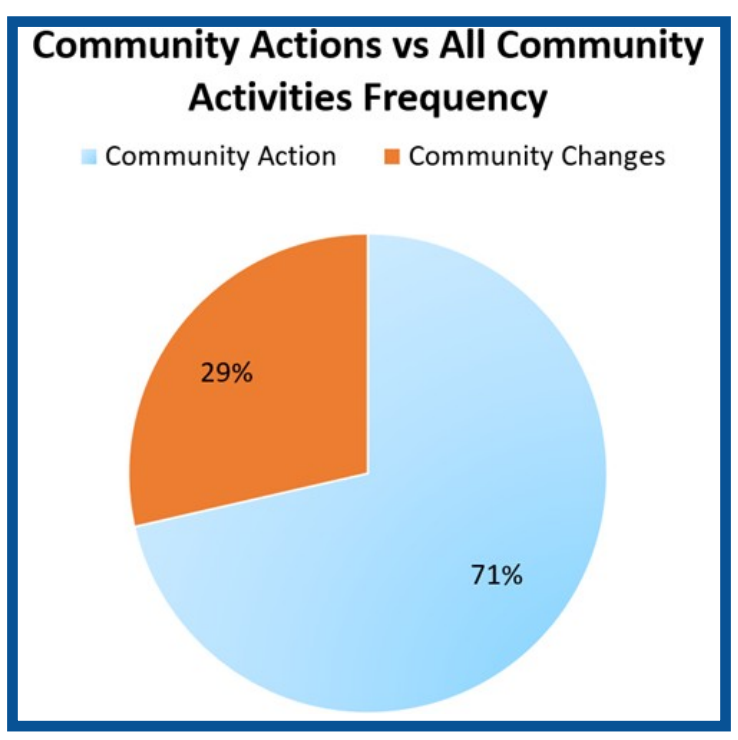

Figure 3

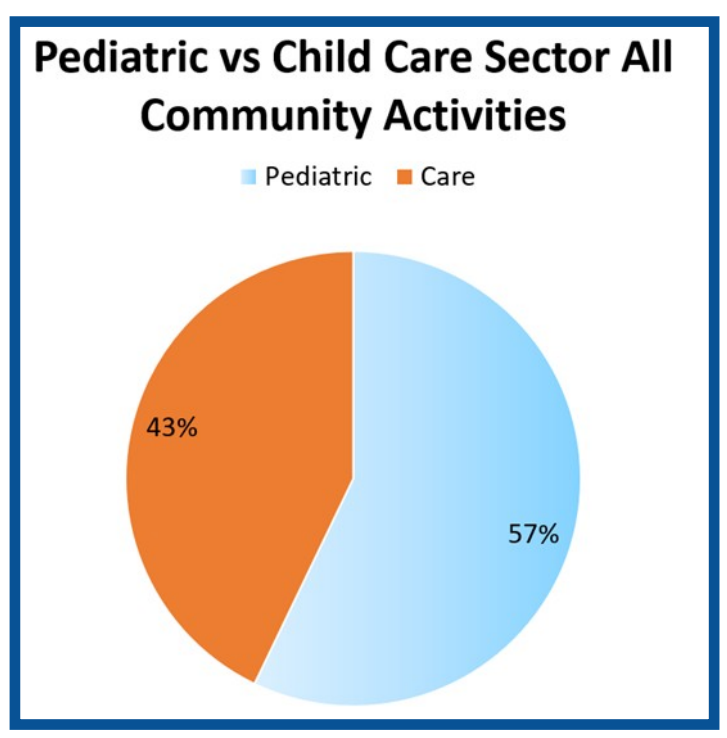

Figure 4

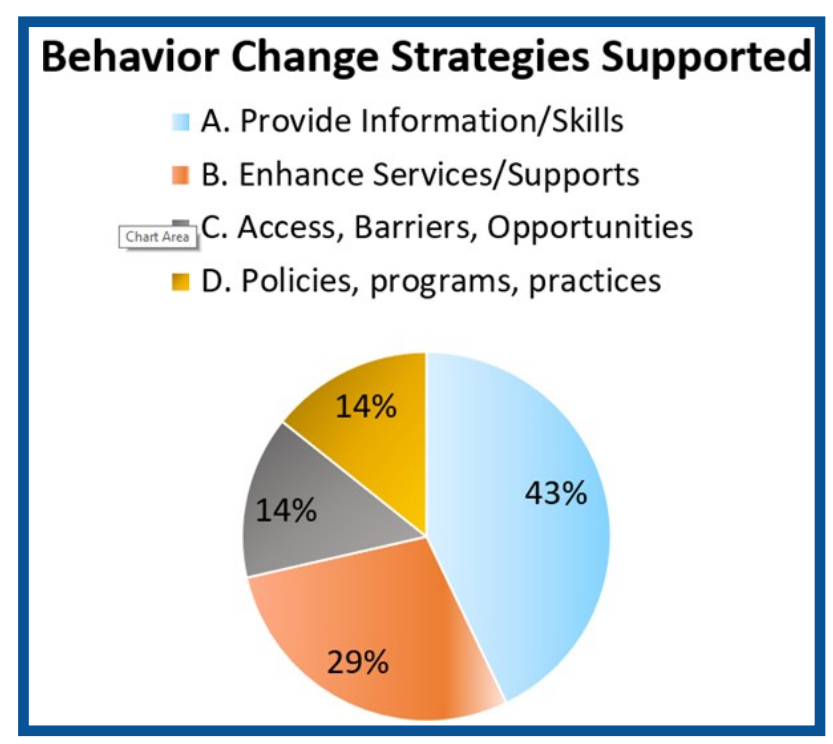

Some other ways of looking at progress are shown in the pie charts (Figures 2, 3, and 4). For example in Figure 2, we see the greater of number of Community Actions needed to produce the desired Community Changes. We also see the larger number of all activities made by the Pediatric vs. the Child Care (Figure 3) sectors. Last (see Figure 4), we can see which categories of accomplishments have changed, with change in (C.) Access, barriers and opportunities and (D.) Policies, programs, and practices with two each being the smallest number compared to (A.) Provide information and skills, and (B.) Enhancing Services and Supports, with four and six, respectively.

What factors affect the rates of community change facilitated by the coalition?

Based on experiences over more than two decades, a number of factors have emerged as key contributors to promoting success in efforts to bridge the Word Gap. Seven key factors include (https://ctb.ku.edu/en/table-of-contents/overview/model-for-community-change-and-improvement/framework-for-collaboration/main):

- A clear and shared vision and mission to guide the work
- Leadership has remained stable
- Action planning is conducted
- Community mobilizers or organizers are hired who can bring about change
- Technical assistance from a consultant or other persons external to the coalition is available, especially to support action planning and intervention.
- Documentation and feedback on rates of community change are maintained
- Organizers make outcomes matter (i.e., bonus grants for high rates of change)
- Training on the Community Tool Box Curriculum, action planning, and supportive external conditions, such as funding.

Figure 5

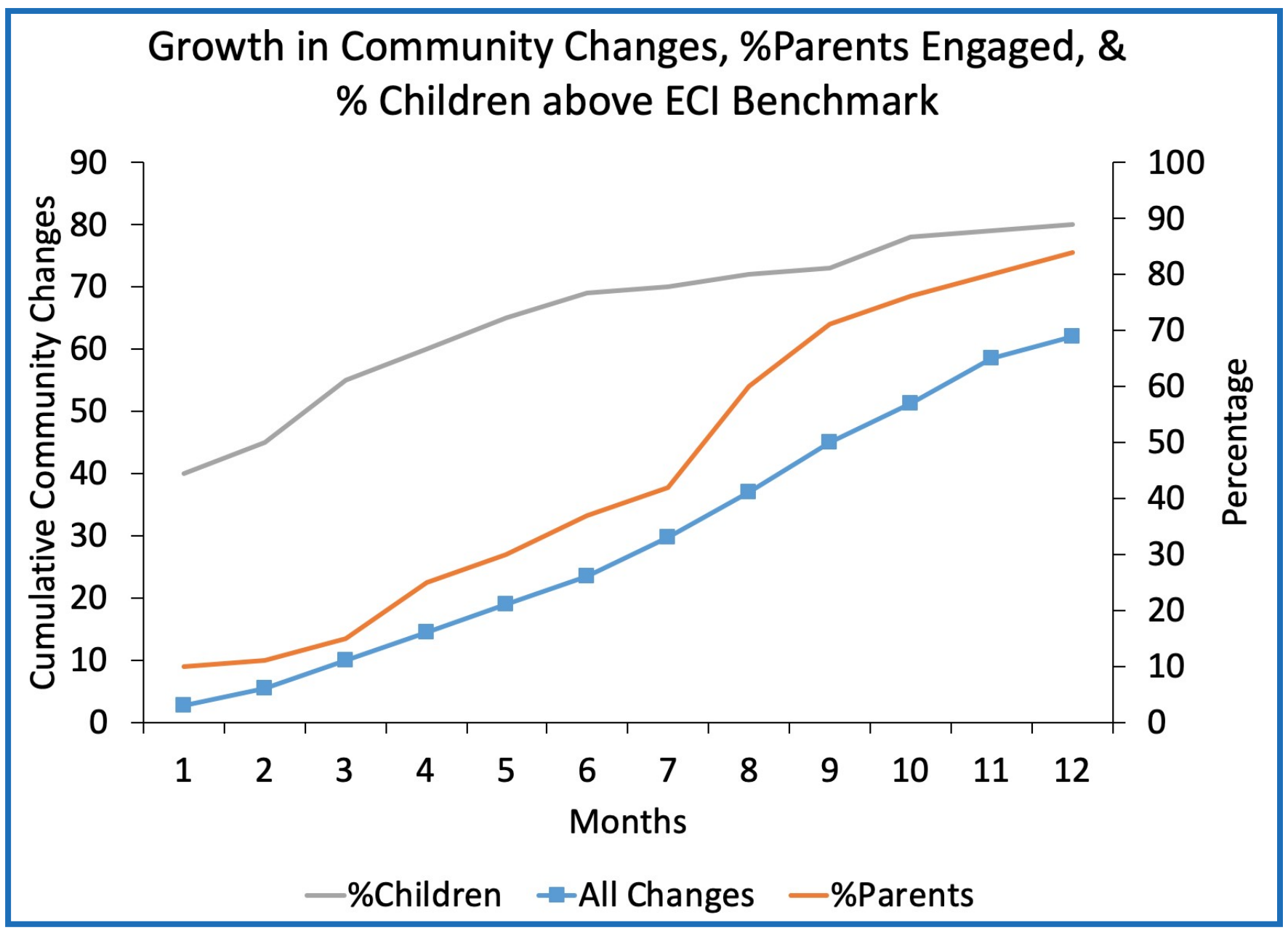

How will this coalition know that its effort have contributed to the targeted outcomes for parents, caregivers, and children in the community?

The CCB also allows tracking of the coalition's targeted outcomes (see Figure 5). To illustrate how tracking can be used, this figure shows growth in the percentage of parents (or caregivers, etc.) who have improved the parents engagement in language interactions with their children. And, we can see the percentage of children who are performing at or above benchmarks in their acquisition of language (Early Communication Indicator).

Promoting Celebration and Renewal

The coalition, groups, and members benefit from frequent feedback on their accomplishments. Thus, scheduled periodic reviews are important opportunities for discussion of progress in facilitating Community Actions and Changes. Particularly, achievement of progress on an initiative or policy deserves special celebration and recognition.

Honoring those most responsible as champions of change in ways that show recognition (e.g., public announcements, small parties) is important. It also helps to focus on small wins along the way by rewarding actions, creating celebrations, highlight successful collaborations all informed by the data on accomplishments.

The data on accomplishments also provides opportunities to identity problems and make changes going forward. Involve all relevant audiences and stakeholders for sectors, and reflect on the specific changes and scope of change likely needed, including changes in the action plan and the vision/ mission if necessary. Review the action plan at least annually and make updates and changes as appropriate and needed to account for lessons learned.

Why this Matters?

Based on lessons learned from successful community change efforts, the coalition is designed around community sectors collaborating to fulfill a grand vision of preventing children's future problems by promoting the changes need to ensure that in all infants and young children in the community are provided with language-learning opportunities across their daily interactions with adults. The plan takes advantage of engaging the participation of all sectors of the community and their members to make the changes in the policies programs, and practices in which they have influence. The multisector community coalition concept stands in contrast to a one large program or project perspective making community change a grassroots effort influenced by vision, efforts, and resources within the community.

Summary

Chapter 7 has provided a plan for documenting community changes that are part of a multisector coalition work toward a common vision of providing the opportunities needed by all children in the community to learn language, succeed in school and a career, and to become contributing citizens. Documenting accomplishments and using data to inform progress and renewal along the way were explained.

Thank you for your efforts to act collaboratively to provide children with enriched language learning environments that promotes growth in young children's language development and school readiness. We wish you much success in your endeavors!

EPILOGUE

This Action Planning Guide has posted markers on the winding road of planning for full participation in our communities. Action planning includes:

Convening a planning group in your community that consists of:

- Key officials
- Grassroots leaders
- Representatives of key sectors

Representatives of ethnic and cultural groups

- Listening to the community
- Documenting problems that affect full community participation for people, including those most impacted
- Identifying risk and protective factors
- Developing a framework for action
- Becoming aware of local resources and efforts
- Refining your group's vision, mission, objectives, and strategies
- Determining what community sectors should be involved in the solution
- Developing a tentative list of changes to be sought in each sector
- Building consensus on proposed changes
- Outlining action steps for proposed changes
- Documenting progress on bringing about community and systems changes
- Renewing your group's efforts along the way

When you complete these activities, *celebrate* (for now)! You have developed an action plan.

The work of transforming communities and systems to promote community participation for all will be made by joining with local people who want to make needed changes. As we do this important work, we realize that we walk the path of those before us. And, eventually, others will carry on this cause to ensure children in every community are able to achieve their full potential across generations.

BWG Action Planning Guide
Glossary

Action Plan: An Action Plan outlines what should happen to achieve the vision and goals of a healthy community around a specified topic. Desirable changes and proposed activities, timelines, and assignment of accountability provide a detailed road map (the Action Plan) for collaborators to follow.

Action Planning: Action Planning increases your community's ability to work together to affect conditions and outcomes that matter deeply to its residents over time and across issues of interest. Action planning involves conducting an active process leading to an action plan comprised of action steps.

Action Steps: Action steps are the specific activities and procedures to be undertaken to reach the goals that your organization/coalition has set forth within its action plan. Each step should describe specific behaviors to be taken to address the following questions: (1) What will occur? (2). How much, or to what extent will these actions occur? (3) Who will carry out these actions? (4) When will these actions will take place and for how long? (5) What resources (such as dollars and staff) are needed to carry out the proposed actions?

Agents of Change: Agents of change are the key individuals and groups who will work to address the issues that matter to your community.

Community: A community is a group of people who share a common place (i.e., a rural community or urban neighborhood) and share common experiences. Communities are often referred to as neighborhoods or affiliations (i.e., faith-based community). Community may also refer to characteristics of participants (i.e.., age group or ethnic, cultural or linguistic identity) and geography (i.e., the Northeast Community, Westlake School District).

Community Action: Community Action is the process of making positive changes through the planned actions of an organized group, coalition, sector, or community.

Community Check Box (CCB): The Community Check Box is an online web-application that a community may use to document its accomplishments toward specified goals, generate online graphs and reports that facilitate reflection about what has been (and has not been) accomplished overall and within community sectors. The CCB also has tools and links to online supports for renewing effort and making improvement toward meeting goal(s).

Community Coalition: A Community Coalition is a group of individuals and/or organizations with a common interest who agree to work together toward a common goal.

Community Leadership Team (CLT): A Community Leadership Team is leadership group of community members representing the multiple sectors of interest needed to plan and implement community and system change.

Community Systems Change: Community Systems Change refers to new or modified programs (e.g. after-school programs), policies (higher fines for selling illegal products), and procedures (e.g. improved access to healthcare services) that modify local conditions. It is believed these changes are an intermediate outcome in the long process of community health and development improvement.

Community Tool Box (CTB): The Community Tool Box is a free, online resource for those working to build healthier communities and bring about social change.

Documenting Accomplishments: Documenting Accomplishments is the active process recording the individual efforts a community undertakes to conduct its Action Plan. Such documentation makes it possible for a community to communicate its accomplishments to itself, its sectors, and other stakeholders (e.g., parent groups, funders).

Documenting Progress and Using Feedback: Documenting Progress and Using Feedback helps a group understand what they are doing, how it contributes to their goals, and areas needing adjustment of effort given lack of progress.

Healthy Environments: Healthy Environments are those in which children and families have access to the supports they need to reach developmental, health, and educational milestones, thrive, and overcome the stress of economic disadvantage and poverty.

Inequity: The Inequities referred to in this Guide are discrepancies that have been documented in peer-reviewed, data-based studies between children experiencing poverty compared to children from more advantaged backgrounds that are linked to later educational, health and other life outcomes.

Intervention Decision Making Model: Intervention Decision-Making Model refers to using data to help make decisions about interventions. In Chapter 5 of this Guide, examples of interventions are provided that include changes in policies, programs, and practices that can be implemented within specific sectors (such as those interventions used in child care programs or in home-visiting programs).

Information Bank: An Information Bank is a repository of information that can be accessed online, through the internet, helpful to this action plan. Online links direct one to the needed information. An example is the TALK repository of talking tips at https://talk.ku.edu/.

Language Nutrition: Language Nutrition is a term created to describe the use of adult language as an experiential nutrient that young children need to learn language well. The term refers to rich language-learning experiences through interactional opportunities (quantity and quality) with adults to hear and use language known to promote neurological, social, and linguistic growth (Weldon et al., 2014; Zauche et al., 2017; Zauche, Thul, Mahoney, & Stapel-Wax, 2016). This metaphor reinforces the point that just as a child needs adequate nutritious food for physical development, so too does a child need adequate quantity and quality of language input for his or her brain and language development

Low Social Economic Status (SES): Low SES is a term used to describe one's status relative to economic opportunity, disadvantage, and poverty. Socioeconomic status (SES) most often incorporates a number of characteristics correlated with low income including educational attainment, unemployment, financial insecurity, and sometimes subjective perceptions of social or occupational status and social class (e.g., blue collar worker). SES is a consistent and reliable predictor of a number of health and educational outcomes across the life span.

Mission: An organization's Mission (Statement) describes *what* the group is going to do and *why* it's going to do it. It is a concrete, action-oriented statement that refers to a problem or a goal the organization is planning to address to overcome it.

Multi-Sector Partnership: A Multi-Sectoral Partnership is a collaboration among community partners that may come from, or represent, different stakeholders or agencies (e.g. health care, schools, government) and that shared a vision or mission.

Neural Pathway: Neural Pathways are the nerve cells in the body along which information travels to the brain. The are still developing in babies and young children, stimulated to the language they hear and the experiences they have. Language Nutrition helps build the brain.

Objectives: Objectives refer to the tasks planned to occur during the project by a specific date that contribute toward attaining the project's goals, mission, and vision.

Part C – Infant/Toddlers Services: Part C (Infant/Toddler) Services are provided to infants and young children birth to age three with documented developmental delays and disabilities as part of the U.S. Department of Education Individuals with Disabilities Act. The act provides funding to local programs to provide early intervention services to children as described in their Individual Family Service Plan (IFSP) that documents the child and family supports needed based on referral and assessment data.

Rate of Change: Rate of Change refers to the speed with which desired changes are observed/documented to occur during the project. Specifically, rate of change = number of changes per unit of time (week, month, or year). Rate of change can be documented in the project's online Community Check Box application (Chapter 8).

Renewal: We use the term Renewal to refer to the process of using the project's data (both implementation data for the Community Check Box and other sources) to guide decisions about the project's progress. Renewal means the process of determining where shortfalls in implementation are occurring, and redressing them through a renewal of effort (as determined by the Community Leadership Team) going forward. Renewal is based on knowledge from project data regarding what is being accomplished and what needs to improve.

Sectors: Sectors are various partner organizations within a community (e.g. business sector, education sector, health sector) that share common social, political, economic, cultural or religious interests.

School Readiness: School readiness refers to a child's readiness to learn in preschool and kindergarten. Children are ready because they have attained the requisite skills needed to learn what will be taught. Readiness skills are determined by a program's, districts', and state's standards. There is not a single, universally accepted measure of school readiness.

Strategies: Strategies are procedures (recipes) describing what needs to be done to achieve a particular outcome or result. Most strategies in this Action Guide are supported by prior evidence based on successful use by others. Some strategies in the Guide are recommended for use by parents and caregivers with children; others are recommended for use by the different sectors helping create the specific healthy conditions within the community for young children to learn language and literacy.

Strategic Planning: Strategic Planning is the target process by which a group defines its vision, mission, objectives, strategies, and action plans.

Vision Statement: A Vision Statement is what persons associated with an organization believes are the ideal conditions for the organization or community. A vision statement consists of short phrases or sentences that convey future desired outcomes. Vision statements should inspire action and be broad enough and straightforward enough to be understood and shared by diverse members of the community.

Word Gap: The Word Gap or 30 Million Word Gap refers to the discovery of Hart and Risley (1995) of a disparity in children's spoken vocabulary by 3 years of age associated with inequity in children's language learning experiences at home.

Glossary References

Center for Community Health and Development. (n.d.). *Chapter 1, Our model for community change and improvement..* University of Kansas. Retrieved May 2, 2020, from the Community Tool Box: https://ctb.ku.edu/en/table-of-contents/overview/model-for-____community-change-and-improvement.

Hart, B., & Risley, T. R. (1995). *Meaningful differences in the everyday experience of young American children*. Baltimore: Paul H. Brookes.

Weldon, J. M., Langan, K., Miedema, F., Myers, J., Oakie, A., & Walter, E. (2014). Overcoming language barriers for pediatric surgical patients and their family members. *AORN Journal, 99*(5), 616-632. doi:10.1016/j.aorn.2014.02.005

Zauche, L. H., Mahoney, A. E. D., Thul, T. A., Zauche, M. S., Weldon, A. B., & Stapel-Wax, J. L. (2017). The power of language nutrition for children's brain development, health, and future academic achievement. *Journal of Pediatric Health Care, 31*(4), 493-503. doi:10.1016/j.pedhc.2017.01.007

Zauche, L. H., Thul, T. A., Mahoney, A. E. D., & Stapel-Wax, J. L. (2016). Influence of language nutrition on children's language and cognitive development: An integrated review. *Early Childhood Research Quarterly, 36*, 318-333. doi:https://doi.org/10.1016/j .ecresq.2016.01.015

REFERENCES

Aram, D. M., & Hall, N. E. (1989). Longitudinal follow-up of children with preschool communication disorders: Treatment implications. *School Psychology Review, 18*, 487-501.

Children's Defense Fund. (2020). The State of America's Children 2020: Child Poverty. Retrieved from https://www.childrensdefense.org/policy/resources/soac-2020-child-poverty/

Hart, B., & Risley, T. R. (1995). *Meaningful differences in the everyday experience of young American children*. Baltimore: Brookes.

Heckman, J. J. (2006). Skill formation and the economics of investing in disadvantaged children. *Science, 312*(5782), 1900-1902.

Hoff, E. (2013). Interpreting the early language trajectories of children from low SES and language minority homes: Implications for closing the achievement gap. *Developmental Psychology, 49* (4), 4-14. doi:10.1037/a0027238

Issacs, J. B. (2012). Starting School at a Disadvantage: The School Readiness of Poor Children. Retrieved from https://www.brookings.edu/wp-content/uploads/2016/06/0319_school_disadvantage_isaacs.pdf

Knudsen, E. I., Heckman, J. J., Cameron, J. L., & Shonkoff, J. P. (2006). Economic, neurobiological, and behavioral perspectives on building America's future workforce. *Proceedings of the National Academy of Sciences, 103*(27), 10155-10162. doi:10.1073/pnas.0600888103

Larson, A. L., Cycyk, L. M., Carta, J. J., Hammer, C. S., Baralt, M., Uchikoshi, Y., . . . Wood, C. (2019). A systematic review of language-focused interventions for young children from culturally and linguistically diverse backgrounds. *Early Childhood Research Quarterly*. doi:10.1016/j.ecresq.2019.06.001

Neuman, S. B., Kaefer, T., & Pinkham, A. M. (2018). A double dose of disadvantage: Language experiences for low-income children in home and school. *Journal of Educational Psychology, 110*(1), 102-118. doi:http://dx.doi.org/10.1037/edu0000201

Nores, M., & Barnett, W. S. (2010). Benefits of early childhood interventions across the world: (Under) Investing in the very young. *Economics of Education Review, 29*(2), 271-282. doi:10.1016/j.econedurev.2009.09.001

Romeo, R. R., Leonard, J. A., Robinson, S. T., West, M. R., Mackey, A. P., Rowe, M. L., & Gabrieli, J. D. E. (2018). Beyond the 30-Million-Word Gap: Children's Conversational Exposure Is Associated With Language-Related Brain Function. *Psychological Science, 29*(5), 700-710. doi:10.1177/0956797617742725

Strauss, V. (2020, April 17). Why covid-19 will 'explode" existing academic achievement gaps. *Washington Post*.

Suskind, D. L., Suskind, B., & Lewinter-Suskind, L. (2015). *Thirty million words: Building a child's brain - Tune in, Talk more, Take turns*. New York, NY: Dutton.

U S. Department of Education. (2015, April). A matter of equity: Preschool equity in America. Retrieved from https://www2.ed.gov/documents/early-learning/matter-equity-preschool-america.pdf

Walker, D., Greenwood, C. R., Hart, B., & Carta, J. J. (1994). Prediction of school outcomes based on early language production and socioeconomic factors. *Child Development, 65*, 606-621.

World Heath Organization. (n.d.-a). The determinants of health. *Health Impact Assessment (HIA)*. Retrieved from http://www.who.int/hia/evidence/doh/en/index1.html

World Heath Organization. (n.d.-b). Ten facts about early child development as a social determinant of health. *Health Impact Assessment (HIA)*. Retrieved from http://www.who.int/maternal_child_adolescent/topics/child/development/10facts/en/

Zill, N., & Resnick, G. (2006). Emergent literacy of low-income children in Head Start: Relationships with child and family characteristics, program factors, and classroom quailty. In D. K. Dickenson & S. B. Neuman (Eds.), *Handbook of early literacy research* (Vol. 2, pp. 347-371). New York: Guilford.

ADDITIONAL RESOURCES

Walker, D., & Carta, J. J. (2020). Intervention research to improve language-learning opportunities and address the inequities of the word gap. *Early Childhood Research Quarterly, 50*, (1) 1-5.

Action Steps for Identified Changes

PLANNING PAGE

Use this page to outline action steps for each identified change to be sought in each community sector.

Community Sector:

Community Change to be sought:

ACTIONS	BY WHOM	BY WHEN	RESOURCES & SUPPORT NEEDED/ AVAILABLE	POTENTIAL BARRIERS OR RESISTANCE	COMMUNICATION
What needs to be done?	Who will take action?	By what date will the action be done?	What financial, human, political and other resources are needed? What resources are available?	What are the policies or practices that are currently in place that could be barriers?	What individuals and organizations should be informed about these actions?
